# Supplementary material for: Policy addressing suicidality in children and young people: an international scoping review
Source: BMJ Open. 2019 Oct 28;9(10):e030699. doi: 10.1136/bmjopen-2019-030699 (PMC6830632; doi:10.1136/bmjopen-2019-030699)
Supplement: Supplementary data [file bmjopen-2019-030699supp001.pdf]

**Supplementary table 1: Mesh Terms & database search results.**						
Database	SEARCH TERMS	HITS after Duplicates removed	To be Read Full Text	Need to access abstract	Rejected = Wrong Topic / Sample / Published prior to 2000	Included studies after screening in FT & Accessing Abstracts
CINAHL via EBSCO	S3 S1 AND S2 Search modes - Boolean/Phrase<br>☐ S2 (MH "Policy Studies+") OR (MH "Public Policy+") OR (MH "Health Policy Studies") OR (MH "Policy Making") OR (MH "Health Policy+") OR "Policy" OR (MH "Hospital Policies+") OR (MH "Guideline Adherence") Limiters - English Language; Language: English; Age Groups: Child: 6-12 years, Adolescent: 13-18 years<br>Expanders - Apply equivalent subjects; Apply related words Search modes -	193	7	9	177	2

**Supplementary table 1: Mesh Terms & database search results.**						
Database	SEARCH TERMS	HITS after Duplicates removed	To be Read Full Text	Need to access abstract	Rejected = Wrong Topic / Sample / Published prior to 2000	Included studies after screening in FT & Accessing Abstracts
	☐ S1 (MH "Suicide+") OR (MH "Suicide, Attempted") OR (MH "Suicidal Ideation") Boolean/Phrase<br>Limiters - English Language; Language: English; Age Groups: Child: 6-12 years, Adolescent: 13-18 years<br>Expanders - Apply equivalent subjects; Apply related words<br>Search modes - Boolean/Phrase					

**Supplementary table 1: Mesh Terms & database search results.**						
Database	SEARCH TERMS	HITS after Duplicates removed	To be Read Full Text	Need to access abstract	Rejected = Wrong Topic / Sample / Published prior to 2000	Included studies after screening in FT & Accessing Abstracts
COCHRANE	1. #1<br>"suicide" or Suicidal:ti,ab,kw (Word variations have been searched)<br>2. #2<br>child* or teen* or adolesc* or young: ti,ab,kw (Word variations have been searched)<br><br>3. #3<br>Policy or Guidance or Strategy or Procedure: ti,ab,kw (Word variations have been searched)<br>4. #4<br>#1 AND #2 AND #3	49 Reviews	0	0	49	0
Medline	S4 = S1 AND S2 Limiters: English<br><br>S3 = S1 AND S2<br><br>S2 = DE "Government Policy Making" OR DE "Education Policy" OR DE "Foreign Policy	12	0	0	12	0

**Supplementary table 1: Mesh Terms & database search results.**						
Database	SEARCH TERMS	HITS after Duplicates removed	To be Read Full Text	Need to access abstract	Rejected = Wrong Topic / Sample / Published prior to 2000	Included studies after screening in FT & Accessing Abstracts
	Making" OR DE "Laws" OR DE "Legislative Processes" OR DE "Older Americans Act" OR DE "Welfare Reform" OR DE "Policy Making" OR DE "Environmental Policy" OR DE "Government Policy Making" OR DE "Health Care Policy" OR DE "Health Care Policy" OR DE "Health Care Reform<br><br>S1= DE "Suicide" OR DE "Assisted Suicide" OR DE "Suicidology					
PsychInfo	TOTAL SUM of S6 and S5 after duplicates removed<br><br>S6= S1 AND S2 Narrowed by: Age (6-12yrs); Age Adolescence (13-17); Language – English<br><br>S5 = S1 AND s2 Narrowed by Age Adolescence (13-17); Language – English.<br><br>S4 = S1 AND s2<br>S3=S1 AND S2<br><br>S2 = DE "Government Policy Making" OR DE "Education Policy" OR DE "Foreign Policy Making" OR DE "Laws" OR DE "Legislative Processes" OR DE "Older Americans Act" OR	43<br><br><br>(13)<br><br><br>(44)	2 But duplicates of studies found on CINAHL.	0	41	0

Gilmour L, *et al. BMJ Open* 2019; 9:e030699. doi: 10.1136/bmjopen-2019-030699

**Supplementary table 1: Mesh Terms & database search results.**						
Database	SEARCH TERMS	HITS after Duplicates removed	To be Read Full Text	Need to access abstract	Rejected = Wrong Topic / Sample / Published prior to 2000	Included studies after screening in FT & Accessing Abstracts
	DE "Welfare Reform" OR DE "Policy Making" OR DE "Environmental Policy" OR DE "Government Policy Making" OR DE "Health Care Policy" OR DE "Health Care Policy" OR DE "Health Care Reform"<br><br>**S1 =** DE "Suicide" OR DE "Assisted Suicide" OR DE "Suicidology"					

Gilmour L, *et al. BMJ Open* 2019; 9:e030699. doi: 10.1136/bmjopen-2019-030699
