# Supplementary material for: Policy addressing suicidality in children and young people: an international scoping review
Source: BMJ Open. 2019 Oct 28;9(10):e030699. doi: 10.1136/bmjopen-2019-030699 (PMC6830632; doi:10.1136/bmjopen-2019-030699)
Supplement: Supplementary data [file bmjopen-2019-030699supp002.pdf]

**Supplementary Table 2: Reasons for Exclusion**			
Source	Total number excluded	Reason for Exclusions	Number
International Policy Documents	4	Not Transferable	2
		Not about suicidal CYP	1
		A review of reviews of interventions	1
UK Policy Documents	3	Not about suicidal CYP	2
		Newer version of report available	1
Journal Articles from Databases	6	Not a policy document or review of policy	5
		Not about suicidal CYP	1
From Experts	2	Not about suicidal CYP	2
