# Supplementary material for: Policy addressing suicidality in children and young people: an international scoping review
Source: BMJ Open. 2019 Oct 28;9(10):e030699. doi: 10.1136/bmjopen-2019-030699 (PMC6830632; doi:10.1136/bmjopen-2019-030699)
Supplement: Supplementary data [file bmjopen-2019-030699supp003.pdf]

Gilmour et al Supplementary Table 3 bmjopen-2019-030699

**Supplementary Table 3: Data Extraction Template.**		
	Info to be extracted	Data
Document Classification	Title of Document	
	Authors	
	Year of Publication	
	Years policy covers	
	Country	
	Is this a new strategy / document or an update of a previous version?	
	Type of Policy Document. (Is it a Policy / Strategy / Guidelines / Review?	
Policy Drivers	Policy or document purpose and Aims. (Does it include any reference to CYP suicide?)	
	Who is subject to the policy, and is adherence compulsory? (Is it aimed at clinicians or services?)	
	Does the policy cover the life-span or is it targeted at a specific age-range?	
	Is this policy universal, selective, or indicated? (WHO, 2010) Or does it include all aspects of prevention activity.	
	Does the policy relate to or feed into any other policies or programs?	
OUTCOMES	Does the policy / strategy / guidance have evaluation measures built in?	
	Who is accountable for ensuring that the policy / strategy is implemented?	
Key concepts for Review Questions	How does the policy / document relate to suicidal CYP? e.g. It is about mental health and well-being for CYP OR It is about CAMHS services OR It is about Suicide	

	What does the policy / document say specifically about the treatment and care of CYP who are suicidal?	
Definitions	Are key-words clearly defined and what definitions are used for self-harm for example?	

*WHO, (2010), Towards Evidence Based Practice, World Health Organisation, Western Pacific Region.

Gilmour L, *et al. BMJ Open* 2019; 9:e030699. doi: 10.1136/bmjopen-2019-030699
