# Supplementary material for: Policy addressing suicidality in children and young people: an international scoping review
Source: BMJ Open. 2019 Oct 28;9(10):e030699. doi: 10.1136/bmjopen-2019-030699 (PMC6830632; doi:10.1136/bmjopen-2019-030699)
Supplement: Supplementary data [file bmjopen-2019-030699supp004.pdf]

Gilmour et al (TBC) Supplementary Document 4. Ref.

Supplementary Table 4: Mapping the policy documents							
Policy Classification	Country	Author	Document Name	Year	Summary of Document Purpose	How does the policy relate to CYP who are suicidal?	How does the policy address the treatment and care needs of CYP who are suicidal?
International Guidance.	Worldwide	United Nations	Transforming our world: The 2030 Agenda for sustainable development	2015	Sustainable development goals and action plan that all countries who are members of the united nations have agreed to implement. These goals and actions support working towards a world free from poverty, hunger and inequality, where everyone has equal human rights.	Goal 3.4: to reduce premature mortality from non-communicable diseases by one third.	It does not specifically mention children and young people who are suicidal.
	Worldwide	WHO	Mental Health Action Plan 2013-20	2013	An action plan to provide leadership and direction in the prevention, treatment and care of mental disorders, It aims to promote the human rights of	It recognises young people as being a priority group in relation to suicide prevention and intervention.	It states that countries should develop suicide prevention strategies, that give particular consideration to at risk groups – and specifies "youth".<br>It speaks to children with mental disorders having

					persons suffering mental ill health, and mental well-being generally.		access to psycho-social, non-medicalised treatments, but does not provide detail as to whether children who are suicidal are to be considered within this group.
	Worldwide	WHO	Preventing Suicide a Global Imperative	2014	Resource to encourage member states to develop suicide prevention strategies.	It identifies that suicide is major health issue for young people.	Provides a description of generic treatment options that may be considered helpful for all persons across the lifespan who have attempted or are thinking about suicide. (These are taken from the WHO's mhGAP Intervention Guide (2010)) It notes in relation to assessment of individuals who present as suicidal that this should be conducted in all person aged 10 years and above, but does not state why this age was selected. Otherwise there is no specific direction regarding the care and treatment of

							children and young people who are suicidal.
	Worldwide	WHO	mhGAP Intervention Guide for mental, neurological and substance use disorders in non-specialized health settings V2	2016	A model guide of effective and evidence based interventions to be adapted for local context.	It identifies children and adolescents as being a “special population”. It does not differentiate between treatment and interventions for children and young people and adults who are suicidal. It offers assessment and intervention guidance which are the same for every-one over ten years of age. It does not state why age ten is used as a marker.	There is a module dedicated to Child & Adolescent Mental & Behavioural Disorders. Within this module there are two mentions of suicide:<br>1) Under the heading “2.5 Psychoeducation for emotional problems/disorders including depression in adolescents” (WHO, 2016, p 88) it states that if the YP notices thoughts of suicide then they should talk to someone they trust, and re-attend for mental health support.<br>2) Within a list a symptoms to assess for, when assessing for moderate to severe depression in adolescents.<br>There is a module detailing the assessment, management and follow up for people who might

							be at high risk of self-harming and or suicide.
National Suicide Prevention Strategies	Australia	Department of Health and Ageing – Australian Government.	Life: A Framework for prevention of Suicide in Australia	2007	National suicide prevention strategy covering the life-span.	This is an over-arching suicide prevention strategy and it does not mention CYP who are suicidal specifically. References to young people include providing support for children who are survivors of childhood abuse, and taking a whole school approach to children's mental health and wellbeing (prevention).	Not specific to children and young people who are suicidal.
	England	Department of Health	Preventing suicide in England: A cross-government outcomes strategy to save lives	2012	Sets out the government's objectives and action plan to reduce suicide rates.	Identifies CYP as a priority group. Within a section dedicated to CYP the policy gives demographic and background information about higher at-risk groups of CYP; those who have or are being bullied, children who	Mostly refers to universal and targeted prevention approaches; whole school approaches etc. and generic mental health provision. It details government approaches to tackling underlying issues such as bullying and child abuse. It does not directly address interventions and

						are looked after and accommodated etc.	treatments specifically for CYP who are actively suicidal other than stressing that early identification and assessment of at risk children vital.
	Ireland	National Office for Suicide Prevention	Connecting For Life: Ireland's National Strategy to Reduce Suicide 2015-2020	2015	National strategy to prevent suicide. Sets out national vision and action plan.	This policy outlines an action plan for delivering universal, targeted and integrated services for individuals who are suicidal and specifies youth services as being a priority area.	Specifically in relation to interventions for CYP who are suicidal the policy states that there should be "enhanced supports" and "enhanced availability". It recommends early intervention and psychological supports being provided and available to CYP who are at risk of suicide at both the primary care level, and secondary level including CAMHS.
	Northern Ireland	Department of Health	Protect Life 2	2016	This document is an update on the previous national suicide prevention strategy and covers the period 2016-2021. Its sets out five key aims: understanding suicidal behaviour	This strategy covers the lifespan, and includes CYP. It provides demographic information on CYP and suicide and self-harm in NI.	It notes that CYP are not a priority group for suicide prevention as the overall numbers of suicide amongst this population are low. It recognises however, that there has been an increase in the numbers of CYP who die by

					in NI better; improve responses to people who are suicidal; prevent suicide; support recovery; support those bereaved by suicide.		suicide and the high numbers of CYP who self-harm. In relation to treatments and care of CYP who are suicidal the strategy states that if those working with CYP are able to better identify risk factors and there is easier access to CAMHS services this would support prevention. It also outlines the role of CHILDLINE in providing support and counselling to CYP, and Crisis Intervention and Support Teams to assess children who present at hospital. It does not provide clear direction in the treatment and care of CYP who are suicidal.
	New Zealand	Associate Minister of Health	New Zealand Suicide Prevention Strategy	2006	National Strategy for suicide prevention.	This document replaces the New Zealand Youth Suicide Prevention Strategy, and moves to a generic life-span approach, although acknowledges that there may be priority	This document does not specifically address the care and treatment needs of children and young people who are suicidal. It provides a review of the literature, the context for suicide prevention, and sets

						groups. In a section headed "Issues relating to Age" p19, it states that young people remain a focus as they still have high rates of suicide amongst this population.	seven goals which are applicable across the life span. These goals include improved care for people who are suicidal.<br><br>Within a section headed "context" it states that although moving to a universal approach, targeted interventions for youth will still be needed.
	New Zealand	Associate Minister of Health	A Strategy to Prevent Suicide in New Zealand: A draft public consultation document.	2017	The draft framework for a new National Suicide Prevention Strategy that has three main elements. It outlines a vision (everyone will have a life worth living); a purpose (to reduce the suicide rate); to provide pathways (to increase protective factors and reduce risk).	Young People aged 15-24 are recognised as a target group.	One of the key objectives of the strategy is to recognize and support people in distress and to support people after a suicide attempt or self-harm. Of the prevention activities that are described that may be helpful that pertain specifically to CYP the focus is on improving support in the school setting: supporting teachers to respond to students who are distressed or following a suicide attempt; training

							teachers to talk to students who are distressed or been affected by suicidal behaviour; partnering psychologists and counsellors with schools to improve access to psychological support for CYP.
	USA	U.S. Surgeon General and the National Action Alliance for Suicide Prevention	2012 National Strategy for Suicide Prevention: GOALS AND OBJECTIVES FOR ACTION	2012	National Suicide Prevention Strategy which aims to guide suicide prevention activity.	The policy document includes suicide prevention guidance for youth, but is generic in its approach.	The policy outlines demographic information that highlights that suicidal behaviours are more common amongst young people aged 15-24yrs than adult populations. Goal 1 – includes references to school based prevention programs, but not interventions. Goal 5 - refers to the need to develop interventions for all groups of people at risk, and that these should be sensitive to and designed around the different needs of these groups. This is not

							specific to children and young people.
	Scotland	Scottish Government	Suicide Prevention Strategy	2013-2016	An update on previous national suicide prevention strategy (The first of which was 2003). A list of commitments and recommendations.	This document sets out the government is committed to reducing suicide and sets out a target of improving responses to people in distress.	It doesn't mention children and young people specifically.
	Sweden	Public Health Agency of Sweden	NATIONAL ACTION PROGRAMME FOR Suicide prevention	2008	This is taken form a brochure of the National Strategy, as the actual strategy is not available in English.	The national strategy has nine overall objectives, and states that their overall aim is that no-one should feel suicide is the only option they have. It recognizes young people as being a high risk group".	The brochure states that the suicide rate amongst 15-24year olds has remain unchanged, despite a reduction in the overall suicide rate over the last 15years.<br>It does not differentiate between the treatment and care that should be given to CYP who are suicidal, although does mention under the heading "Improve medical, psychological and psychosocial initiatives" amongst a list of example initiatives some with a particular youth / student focus.
	Wales	Welsh Government	Talk to Me 2: Suicide and Self	2015	National Strategy to prevent suicide.	The strategy covers the life span, but	Demographic background information

			Harm Prevention Strategy for Wales 2015-2020		An update on a previous policy.	identifies that certain groups of CYP are priority: Looked after children, Care leavers, Children and young people in the Youth Justice System Bullied or victimised children and young people, Survivors of abuse or violence including sexual abuse and domestic violence and children and young people with a background in vulnerability.	is provided. Strategy focuses upon universal school based prevention programmes and targeted prevention activities such as school based counselling services. In relation to CYP who are suicidal it states that services should be accessible for those in crisis.
National Mental Health Strategies.	CANADA	Mental Health Commission	Changing Directions Changing Lives: The Mental Health Strategy for Canada.	2012	National Mental Health Strategy covering the lifespan.	This policy recognises suicide as being an important issue and addresses it directly. It also recommends CYP are a priority group.	Sets out objectives which include improved screening and assessment for suicide, and improved access to services. These are generic recommendations for all age groups. In relation to children and young people specifically it states that governments should address inequality in accessing to services psychotherapies

							and counselling. The situation is described as urgent, with families who do not have access to financial resources having to wait up to a year for treatment. This is not specific to suicidal children and young people.
	CANADA	Mental Health Commission	The Mental Health Strategy for Canada: A Youth Perspective	2015	National Mental Health Strategy written from the perspective of young people. Translates the original policy document into lay language.	Generic document – addresses mental health generally, but preventing suicide is identified as a priority. Talks about access to services and specialist help being available to people when they need it.	Not specific to the treatment and care of suicidal children and young people but states that mental health services should be more accessible to people of all ages.<br>It highlights examples of good practice in service provision and suicide prevention for children and young people. E.g. The Thunder Bay Youth Suicide Prevention Task Force (30 organisations working collaboratively to prevent suicide and provide an immediate response) & Youth advocates (Young people with lived experience).

							It addresses race and cultural issues in relation to suicide risk for YP: increased risk for Inuit and Metis young people. It refers to organic culturally sensitive initiatives such as The Inuit Tapirit Kanantami National Youth Council, and community health resources that offer programs of support to children and young people at risk of suicide.
	England	HMG/DH	No health without mental health A cross-government mental health outcomes strategy for people of all ages	2011	National Mental Health Strategy across the life span. Aims to address the disparity between how mental health and physical health are treated. Has six main objectives. Aims to mainstream mental health.	Adopts a lifespan approach but identifies CYP as a group with particular needs, and recommends age appropriate services and interventions.	Gives background demographic information on mental health issues including the numbers of children and young people who have self – harmed. States that all people who come into contact with CYP should be aware of the issues in relation to self-harm, as they do not always attend hospital. States that reducing suicide remains priority government target.

							Reduced numbers of people of all ages harming themselves is set as a measure of success of the strategy.<br><br>No specific references to CYP who are suicidal.
	Ireland	Department of Health	A vision for change	2006	Provides a vision and model for mental health service provision.	This document addresses a wide range of mental health issues and service provision. Within this there is a chapter dedicated to the provision of mental health services for CYP (Ch. 10) and a section dedicated to Suicide Prevention within Ch. 15 on Special Categories. It does not identify CYP as being a priority group.	Within Ch.10 it states that all children who present with deliberate self-harm should be assessed by the child and adolescent mental health team, and if appropriate receive treatment. Within this section it also recognises adolescence as being a period of increased risk of suicide.
	Scotland	Scottish Government	Mental Health Strategy	2017 - 2020	National Mental Health Strategy covering the life span.	The documents provides a focus on prevention and early intervention for children and young people. It includes actions relating to	It does not specifically address the treatment and care needs of CYP who are suicidal. It states that Suicide and Self-Harm continue to be

						support being available in schools, delivery of CAMHS services, and access to support and services when they are needed.	a priority area that will be addressed in a new suicide prevention strategy in 2018.
	Wales	Welsh Government	Together for Mental Health	2016	Ten year National strategy for Mental Health. Aims to improve the mental health and well-being of people of all ages in Wales adopting a life span approach to policy.	The policy addresses generic mental health needs and provision of mental health support services for CYP. It highlights the need for targeted interventions for those most at risk, and early identification and prevention.	It does not specifically address the treatment and care needs of CYP who are suicidal.
National Frameworks for Children & Adolescent Mental Health	CANADA	Mental Health Commission Canada	Evergreen: A Child and Youth Mental Health Framework for Canada	2010	Framework of values and strategic direction for the provision of mental health services for children and young people.	Policy concerned with promoting mental health and well-being amongst young people, and provision of mental health support services for CYP and families across Canada, but does not specify suicidality.	Does not mention the treatment and care of CYP who are suicidal. Lists recommendations about making services accessible (available in the evenings and weekends, accessible to YP directly, and child / youth centred), including creating "one-stop shops", however

							does not specify children and young people who are suicidal. The only reference to suicide is in a direct quote by a young person talking about how there is a lack of education about mental health issues and that many Aboriginal young people are largely unaware of mental health issues but have high rates of suicide.
	England	Department of Health	National Service Framework for Children, Young People and Maternity Services: The Mental Health and Psychological Wellbeing of Children and Young People, Department of Health and Department for Education and Skills (2004) –	2004	This is part of the national framework for standards for child health services. It is the standard for children who need to access mental health and psychological services,	This standard sets out a vision for all CYP to have access to specialist mental health support services when they need it. It only directly refers to CYP who are suicidal once in relation to out of hours support.	The standard is not specific to CYP who are suicidal, however it does state that CYP should have access to support services when they need them, that they should have time to build a therapeutic relationship, and that non-attendance of clinical appointments should not be a trigger for closing cases. The standard states that services need to be creative and flexible in how they engage with CYP.

	England	Department of Health & Department of Education	Transforming Children and Young People's Mental Health Provision: A Green Paper	2017	Green paper proposing reform to child and adolescent mental health service provision: the appointment of mental health leads in each school; creation of support teams to asses and act as a bridge between school and CAMHS services; trial a four-week waiting time for CAMHS specialist services.	Policy update to address CYP experiencing distress and or in "crisis" being able to access the right support when they need it but does not directly refer to suicide.	Recommendations about the treatment and care of CYP experiencing distress or being in crisis could be applied to CYP who attempted or are actively thinking about suicide, but it does not specifically address suicidal CYP. Under a section entitled "Improving Crisis Care" there is reference to pilot models of crisis support underway, a new government funded scheme called "Beyond Places of Safety" for people of all ages who are in crisis, and the commissioning of the development of generic and crisis mental health pathways for CYP.
	Scotland	Scottish Government	The Mental Health of Children and Young People: A Framework for Promotion, Prevention and Care	2005 (Due to be updated)	National framework to be used as a guide for local authorities and commissioners of children's services to guide service design and delivery.	Takes a holistic view of children's mental and health and well-being and recognises the role that everyone in contact with CYP have in supporting their mental health.	No specific references to CYP who are suicidal.

	Wales	Welsh Government	Together for Children and Young People: Framework for Action.	2015	National Framework for child and adolescent mental health support services.	The framework provides multi agency guidance for the provision of children and young people's mental health and well-being.	It does not specifically address the treatment and care of children and young people who are suicidal. It does state that there should be a specialist CAMHS pathway, and this includes out of hours support for CYP in crisis.
Guidance Documents	Ireland	Health Service Executive & Child and Adolescent Mental Health Service.	Child and Adolescent Mental Health Services Standard Operating Procedure	2015	Provide clear direction for the delivery of CAMHS services across Ireland, to ensure consistent and transparent approach.	This document outlines the standard operating procedures for CAMHS services in Ireland, and the treatment and services that CYP and their families should expect. This includes CYP who are suicidal.	Generic statements about CAMHS services being accessible, and response times especially for situations where there may be a risk to the young person and an immediate response is required could be applicable to CYP where there is a risk of suicide but this is not clearly stated. The treatment of CYP who are suicidal is only specifically addressed in reference to the fact that CAMHS will accept referrals for CYP with "suicidal behaviours and ideation where intent is present" p15, and in terms of risk

							management listing risk of suicide amongst potential risk behaviours within an in-patient unit.
	New Zealand	Ministry of Health & New Zealand Guidelines Group	The Assessment and Management of People at Risk of Suicide	2003	These National Guidelines are a resource and guide for clinical staff in emergency departments, and psychiatric services working with people in mental distress who are at high risk of suicide or have attempted suicide.	This document specifically states how all persons who are suicidal should be treated and has a small section referencing the treatment of children and adolescents within it.	These guidelines provide specific recommendations on how to respond to and treat anyone who is suicidal, including children and young people. It states that anyone talking about suicide should be taken seriously.<br>In particular to CYP it states:<br>“Self-harm among children is rare and should be treated very seriously…The assessment of suicidal young people should be carried out by a clinician who is skilled in interviewing and working with children and adolescents whenever possible….Risk assessments should draw on information from multiple sources, including the young

							person, their teachers/guidance counsellors, parents etc.”<br>In the section on background information following the guidance it notes that New Zealand has one of the highest rates of suicide amongst young people.
	UK	NICE	Self-harm in over 8s: short-term management and prevention of recurrence	2004	Clinical guidelines for the management of self-harm in people over 8yrs in the first 48hrs following the incident of self-harm. These guidelines apply to everyone regardless of whether there is an underlying mental health illness.	The definition of Self-Harm used includes all acts of self-harm regardless of suicidal intent. These guidelines therefore apply to CYP (8-16yrs) who have attempted suicide, although this is not specifically stated.	It recommends that CYP who have self-harmed are admitted overnight to a paediatric ward (potentially adolescent ward if preferred by those aged 14yrs+), and fully assessed before discharge. The guidelines state that they should be assessed by someone trained and with experience in assessing CYP who have self-harmed. It states that assessment should be the same as for adults but also include holistic assessment of family situation etc. The only direct reference to suicide is contained

							within the generic description of "Assessment"; it is included in a list of factors to be assessed. It goes on to specify that child and adolescent mental health practitioners assessing CYP who have self-harmed should be trained, skilled in risk assessment, supervised regularly, and have access to senior support. Family are to be recommended to remove access to means of self-harm in the immediate aftermath of the incident.
	UK	NICE	Self-harm in over 8s: long term management and prevention of recurrence	2004	Guidelines for the long-term psychological treatment and management of self-harm. It aims to improve the quality of care that they receive, and reduce repetition of self-harm.	The definition of Self-Harm used includes all acts of self-harm regardless of suicidal intent. These guidelines therefore apply to CYP (8-16yrs) who have attempted suicide, although this is not specifically stated.	Under the heading "Risk Assessment" there are two references to suicide, which it states should be assessed in relation to risk (risk of self-harm & past and present suicidal intent). However it warns against using risk assessment tools to assess this risk.

							Under the heading "Access to Services" it states that children and young people who self-harm should be able to access psychological therapies and treatments from child and adolescent mental health services."<br><br>CAMHS workers who are working with CYP who self-harm should consider safeguarding procedures, and where children are referred to CAMHS under safeguarding procedures then there should be a multi-agency approach and consideration of common sense framework.<br><br>Under the heading "Primary Care" it recommends that consideration be given to referring CYP who present with self-harm to CAMHS for specialist help. Factors to consider

							are the parent or carers' distress / anxiety.
	USA	Chun et al, American Academy of Paediatrics	Evaluation and Management of Children and Adolescents With Acute Mental Health or Behavioral Problems. Part I: Common Clinical Challenges of Patients With Mental Health and/or Behavioral Emergencies	2016	To provide clinical guidance to staff in the USA primarily working in A & E departments, but for all clinicians who work with children with emotional and behavioural problems on dealing with paediatric mental health emergencies. It is not intended to be used as a standard of care and is a recommended practice as opposed to enforced.	This document has a section dedicated to dealing with children who present to the Emergency Department in hospital following a suicide attempt.	This document provides very specific recommendations on the assessment and care of children where there has been a suicide attempt. This includes medical assessment as well as suggestions for psychological assessment. It states that although there is no recognised effective risk assessment tool for suicide, it lists criteria such as not engaging in safety planning and remaining intent upon dying that if present suggest the child should be admitted to hospital for their own safety. It suggests that otherwise the child might be referred for outpatient treatment and that if this is unavailable or waiting times are long mental home providers (Patient centred care co-ordinated by their own

							doctor) are encouraged to provide support in the interim.
	USA	Michael L. LeFevre, MD, MSPH, on behalf of the U.S. Preventive Services Task Force*	Screening for Suicide Risk in Adolescents, Adults, and Older Adults in Primary Care: U.S. Preventive Services Task Force Recommendation Statement.	2014	Recommended clinical guidance for primary care settings in relation to the assessment of suicide risk.	Specifically addressed assessing the suicide risk in adolescents.	There is limited evidence for any treatments for adolescents at risk of suicide, and not enough evidence to support the potential benefit or harm of either screening for risk of suicide, or not.
Miscellaneous reports about policy.	Northern Ireland	Dr Jennifer Betts and Dr Janice Thompson	Mental Health in Northern Ireland: Overview, Strategies, Policies, Care Pathways, CAMHS and Barriers to Accessing Services	2017	This paper provides background information on mental health and illness in Northern Ireland (NI) including demographics on Suicide and self-harm. It details the relevant strategies and policies. It describes the care pathway for treating mental health problems, with specific reference to child and adolescent mental health	Reports on recent changes in the delivery of CAMHS services and attempts to make them accessible. Early intervention is highlighted as a priority. Provides an overview of key mental health policies and refers to objectives to reduce the number of people presenting with self-harm, and suicide prevention activity.	Gives demographic information highlighting that suicide and self-harm are issues for young people in Northern Ireland. Not specific to suicide, but it states that a referral care pathway for CAMHS is currently being developed.

					services (CAMHS); discussing barriers to accessing services.		
	Scotland	Scottish Government	Responding to self-harm in Scotland: Mapping out the next steps	2011	This document is a report from the National Self Harm working group who were tasked with mapping out the next stages following publication of the Truth Hurts in 2006.	This document is a recommendation for policy. It calls for improved responses to people who self-harm across the life span, and recognizes that for most people who self-harm they do not intend to die.	Provides background information and demographic information on self-harm rates in Scotland. It states that young people are more likely to self-harm. Within the proposed objectives for future actions there are recommendations for clear referral pathways for all people who self-harm but is not specific to CYP. The only direct references to the treatment and care of CYP are recommendations on guidance on confidentiality and information sharing where a child is at risk.
	New Zealand	Prime Minister's Chief Science Advisor – Ch. 16: Keren Skegg.	Improving the Transition Reducing Social and Psychological Morbidity During Adolescence	2011	This is a report commissioned by the government to review the evidence on how to improve the	Ch. 16 is devoted to Youth Suicide. It aims to give an overview of the issue and prevention strategies.	Ch.16 of the report considers how the New Zealand Suicide prevention strategy goals apply to young people. It takes each

					outcomes for adolescents transitioning to adulthood.		goal in turn and cogitates how it focuses on youth. In conclusion it argues that the strategy involves actions that can be applied to the issues of youth suicide. It also argues that any programmes aimed at young people must be evidence based.

Gilmour L, *et al. BMJ Open* 2019; 9:e030699. doi: 10.1136/bmjopen-2019-030699
