# Supplementary material for: Policy addressing suicidality in children and young people: an international scoping review
Source: BMJ Open. 2019 Oct 28;9(10):e030699. doi: 10.1136/bmjopen-2019-030699 (PMC6830632; doi:10.1136/bmjopen-2019-030699)
Supplement: Author's manuscript [file bmjopen-2019-030699.draft_revisions.pdf]

BMJ Open is committed to open peer review. As part of this commitment we make the peer review history of every article we publish publicly available.

When an article is published we post the peer reviewers' comments and the authors' responses online. We also post the versions of the paper that were used during peer review. These are the versions that the peer review comments apply to.

The versions of the paper that follow are the versions that were submitted during the peer review process. They are not the versions of record or the final published versions. They should not be cited or distributed as the published version of this manuscript.

BMJ Open is an open access journal and the full, final, typeset and author-corrected version of record of the manuscript is available on our site with no access controls, subscription charges or pay-per-view fees (http://bmjopen.bmj.com).

If you have any questions on BMJ Open's open peer review process please email info.bmjopen@bmj.com

Policy addressing suicidality in children and young people: an international scoping review.

Journal:	*BMJ Open*
Manuscript ID	bmjopen-2019-030699
Article Type:	Research
Date Submitted by the Author:	29-Mar-2019
Complete List of Authors:	Gilmour, Lynne; University of Stirling, NMAPH RU<br>Maxwell, Margaret; University of Stirling, Nursing, Midwifery and Allied Health Professions Research Unit<br>Duncan, Edward; University of Stirling, NMAHP-RU
Keywords:	Suicide & self-harm < PSYCHIATRY, Child & adolescent psychiatry < PSYCHIATRY, Scoping Review, Health policy < HEALTH SERVICES ADMINISTRATION & MANAGEMENT, young people

SCHOLARONE™
Manuscripts

Policy addressing suicidality in children and young people: an international scoping review.

Lynne Gilmour MA (Hons), MRes

NMAPH Research Unit, University of Stirling, Unit 13 Scion House, Innovation Park, Stirling, FK9 4NF. Contact Email: Lyg1@stir.ac.uk Contact Tel: 01786 466102

Prof Margaret Maxwell

NMAPH Research Unit, University of Stirling, Unit 13 Scion House, Innovation Park, Stirling, FK9 4NF

Dr Edward Duncan

NMAPH Research Unit, University of Stirling, Unit 13 Scion House, Innovation Park, Stirling, FK9 4NF

Word Count: 6,638

Key-words: Suicide; policy; scoping-review; international; children; young people.

ABSTRACT (284 words)

Background: Suicide remains the second leading cause of death in children and young people worldwide. International and government policies establish the context for spending on the development and delivery of services. Yet little is known about how policy addresses the specific needs of this vulnerable population. **Objective:** To map key policy documents worldwide and establish how they address the treatment and care needs of children and young people who are suicidal. **Method:** This review employed scoping review methodology to systematically identify relevant key policy documents following a pre-established search strategy and published protocol. Eligible documents were screened against predefined exclusion and inclusion criteria. Data was then extracted from the included policy documents using a predetermined template. Both screening and data extraction were cross-checked by second reviewers. **Results:** Of 339 screened documents, 35 met the inclusion / exclusion criteria and were included in the review. Documents were categorised as international guidance, national policy, and national guidance, and presented in a table that provides a brief description of the policy, alongside how it specifically addresses suicidal children and

For peer review only - http://bmjopen.bmj.com/site/about/guidelines.xhtml

young people. **Conclusion:** Although many of the worldwide and national policy documents recognise children and young people as being a high risk or priority population, most do not explicitly address suicidal children and young people. In general, the national guidance documents were found to convey that if a child or young person is suicidal or has self-harmed they should be assessed by a child and adolescent mental health practitioner but offer no clear recommendations beyond this. These findings highlight a potential gap in policy that could lead to the needs of this vulnerable population being overlooked, and varying interpretations of appropriate responses and service provision.

Strengths & Limitations

- **This is the first scoping review to consider how policy addresses suicidality in children and young people and provides new knowledge.**
- **The review authors employed a rigorous and methodological approach to the identification, screening and inclusion of policy documents, and following a clearly defined protocol.**
- **There is a lack of clear guidance in relation to how to conduct a systematic review of policy documents, which added complexity to the review process and reporting.**
- **The lack of specific reference to suicidal children within policy documents made it difficult to identify relevant documents, and there may be some we were unable to identify for inclusion.**

For peer review only - http://bmjopen.bmj.com/site/about/guidelines.xhtml

BACKGROUND

Suicide is a global health policy priority, with nearly 800,000 lives lost to suicide annually. Suicide is arguably preventable. Reducing suicide rates is a target of the WHO Mental Health Action Plan 2013-2020 (1) in which member states agreed to work towards reducing suicide rates by 10% by 2020 Globally, suicide prevention strategies have been established in 28 countries to date (2).

International and government policies establish the context for the direction of resources for the development and delivery of services. Health policy provides a future vision (internationally, nationally or regionally), sets priorities, and can include an action plan to achieve specific health related objectives (3). Public policies reflect international or national commitment and ambitions to address specific issues, but can vary in whether, and how they translate or relate to practice and whether there is a mandate for action.

Suicide is a leading cause of death of children and young people (CYP) worldwide, second only to accidental death (4). It is estimated that as many as 1 in 3 children in some countries have considered suicide in the past year (5). Although globally developing countries are often identified as having the highest rates of suicide overall, more economically developed countries report some of the highest rates of suicide amongst children and young people (6,7) and in some it is the main cause of death (8).

It is widely acknowledged that children and young people have different needs to adults. A retrospective review of suicides amongst children and young people found that there were even differences between the presenting issues for children under 15 years compared with adolescents (age ranges of adolescents was unspecified by the authors) (9).

Many countries commonly address the health needs of children and young people separately to adults, with discrete policy and service provision, although definitions of what age range constitutes being a child, adolescent or young person varies greatly (10). However, with most suicide prevention

For peer review only - http://bmjopen.bmj.com/site/about/guidelines.xhtml

and mental health strategies now taking a universal approach, there is the potential for the specific needs of children and young people to become lost.

Fortune and Clarkstone (11) highlight the gulf that can often exist between suicide prevention policy and practice. They argue that although policy documents in New Zealand state that every-one who is suicidal should be assessed by a trained mental health professional, services are not adequately resourced to meet the demand. This is not unique to New Zealand, or to suicide prevention policy. The overall political context and policy agenda needs to be analysed in greater depth in order to make sense of the meaning conveyed within policy documents, and attributed to them (12).

Little is known about how policy addresses suicidality in children and young people. Preliminary searches of review databases (Cochrane, DARE, JBI, and the Campbell Collection), found that there had not been a review of worldwide policy in relation to children and young people who are suicidal. Reviews to date have focused upon the effectiveness of prevention and intervention strategies (13,14). Although generating valuable knowledge on the evidence base for interventions, they do not consider the policies behind such strategies and how this shape their focus and direction.

Mapping key policy documents world-wide and identifying how they address the treatment and care needs of suicidal children and young people will: highlight best practice for how policy can influence the resourcing of services; change practice; and identify any gaps in policy provision for this vulnerable population. This knowledge will support countries who wish to develop new policies or further develop existing policies that address suicidality in children and young people. The review question, objectives, search strategy, and inclusion criteria were specified in advance and documented in a published protocol (15).

Objective:

To map key policy documents worldwide and establish how they address the treatment and care needs of children and young people who are suicidal.

For peer review only - http://bmjopen.bmj.com/site/about/guidelines.xhtml

Inclusion Criteria:

Population

The key characteristics of the population were age and suicidality, neither of which have agreed universal definitions. This review was conducted in Scotland and informs a larger study taking place here, concerned with a school aged population. On this basis it was agreed to adopt a definition of a child as anyone under the age of 18 years, in line with: The Children (Scotland) Act 1995 (16), and the UNCRC (United Nations Convention of the Rights of the Child, 1998) (17). Policies solely about populations aged over 18 years were excluded, however policies that cover the lifespan were included.

Suicidal behaviour is defined as acts of self-harm that result in death, as well as those with a non-fatal outcome. Non-suicidal self-injury is a term used to describe self-harming behaviour where there is no intent to die. This is most commonly used in the USA and became a discrete diagnostic category in the DSM V (18). However, in the UK and some other European countries definitions of self-harm are now also often used to include all non-fatal self-harming behaviours regardless of intent, and can include behaviour that may also be described as attempted suicide (19). As this review is concerned with identifying relevant policies for children who are suicidal (had attempted to end their own life or were thinking about suicide), it was agreed not to use the term self-harm as a search term.

Concept

Suicide prevention activities can be divided into three domains: universal activities which are aimed at everybody, including public health education programmes; selected or targeted interventions that aim to reduce the risk amongst specific high-risk groups; and indicated interventions that may include treatments and are aimed solely at individuals presenting with suicidal behaviour. This

For peer review only - http://bmjopen.bmj.com/site/about/guidelines.xhtml

review is solely concerned with identifying policy in relation to indicated activities, aimed at children (under 18 years of age) who are suicidal.

Context

Identified policy documents were assessed for direct relevance to Scotland and the UK, or relevant to the context and population of the UK. Policies relating to indigenous populations such as the Sami populations in Norway, Sweden and Finland, were consequently excluded (20); but generic policies in post-industrial nations with developed economies such as Australia, and New Zealand were included (21,22).

Types of Sources

Suicide prevention, like much health care policy does not sit within clearly defined and labelled singular policy documents. As well as national suicide prevention strategies, there are more generic mental health strategies or frameworks, and national guidelines such as those published by the National Institute of Clinical Excellence (NICE) in the UK. Mapping policy requires a recognition of the variety of formats in which relevant documents may be found. Local government agencies and organisations also have their own individual policies and procedures, however these should reflect the national approach. It was agreed that for the purposes of this review, policy documents would include: policies, policy guidance, strategies, codes of conduct, national service frameworks, national practice guidance, and white and green papers (23). Reviews of policy documents centred on children who are suicidal were also eligible for inclusion as they contribute to the development of what is known in this area.

Given that the review aimed to map the present policy context, and most strategies are updated within a ten-year period, it was agreed to exclude any policy document or review published prior to 2000. Only those available as English Language versions were included.

Search Strategy

For peer review only - http://bmjopen.bmj.com/site/about/guidelines.xhtml

Key words to be used as search terms (Table 1.) were generated from the review question (15). Preliminary searches assisted in the refinement of these terms, and the identification of the most appropriate databases, platforms and websites. These terms were then amended for each of the databases and the exact terms, including any MESH terms and subject headings used recorded (Supplementary table 1.).

TABLE 1: Search Terms

Concept	Key-words
Children and Young People (5-18yrs)	Child*; "young people"; youth; adolesc*; teen*; paediatric
Suicide	Suicide; suicidal;
Policy	Policy; Procedure; Guidance; Strategy
LIMITS	English Language; Published after 2000.

Four databases (CINAHL; Medline; Psychinfo; The Cochrane Database of Systematic reviews) and the websites of the following key government, statutory and non-statutory agencies were searched, focusing on post-industrial nations with developed economies in order to identify those with most applicability to the UK: - for example, WHO; UNICEF, UK Government; Scottish Government; ScotPHO; NICE (UK National Institute of Health Care Excellence); National Office of Suicide Prevention (Ireland); Ministry of Health NZ; Australian Government Website; and the Mental Health Commission Canada. Google, and Google Scholar were also used to identify other policy documents and any relevant grey literature. Leading experts in the field were consulted via email.

All results were screened by title and abstract or executive summary by LG, with MM and ED screening a sample of 20%. Policy documents and articles were all screened in full by LG, and another sample of 20% was independently screened by MM and ED for inclusion. Disagreements were resolved by discussion, with the third reviewer acting as mediator.

For peer review only - http://bmjopen.bmj.com/site/about/guidelines.xhtml

METHOD OF THE REPORT

This review employed scoping review methodology to systematically identify relevant key policy documents following a pre-established search strategy and published protocol (15). Scoping review methodology and guidance first outlined by Arskey & O'Malley (24) and further developed by Levac,(25), and the Joanna Briggs Institute (26) were used to inform the methodological process. The scoping review method was chosen as it allows for the synthesis of different types of study design. Thus, lending itself to the incorporation of different policy document formats (policies, policy guidance, strategies, codes of conduct, national service frameworks, national practice guidance, and white and green papers (23), as well as any relevant existing published policy reviews. Suicidology of children and young people is a newly emerging, highly sensitive and complex area of research, therefore well suited to scoping review methods (24). The review is reported in line with the new PRISMA extension for Scoping Reviews (27). Patients and the public were not consulted as part of this scoping review as it was not appropriate or applicable.

Results

There were 43 records retrieved from Psych-info, 193 from CINAHL, 12 from Medline and 49 from the Cochrane database of systematic reviews. After removing duplicates there was a total of 297 records to be screened by title and abstract. Separate Excel spreadsheets were set up to catalogue the lists of references from each of the databases. After screening these results by title and abstract (completed in full by LG with a second reviewer independently screening for validity) all 8 records to be screened in full text were found on CINAHL, although 2 were also found in duplicate on Psychinfo. Reasons for rejection of records included wrong topic, not in English, and published before 2000.

After screening the 8 articles identified by searching the databases (cross validated by a second reviewer) only 2 met the inclusion criteria (28,29). Five were rejected as they were not policy documents about children and young people who were suicidal, and although one seemed relevant

For peer review only - http://bmjopen.bmj.com/site/about/guidelines.xhtml

in its references to the New Zealand suicide prevention strategy (11), it was neither a policy document, nor a review of policy.

Internet searching was an iterative process, using key-words to search worldwide government, statutory and non-statutory agencies websites, with 39 potentially relevant policy documents identified. Although it is common practice in systematic reviews to screen the references of included documents for other potentially relevant papers, this occurred intuitively throughout the identification of policy documents, with one referencing others within a country. Policy documents were only included for screening if by their title and description they seemed potentially relevant.

The World Health Organisation (WHO) Mindbank database houses links to member states National Suicide Prevention Strategies. The WHO mental health policy and services representative was contacted to request contact details of policy authors or country specific contacts to enquire about English language versions. From these enquiries an English language brochure outlining the content of the Swedish Suicide Prevention Strategy (known to be innovative for its zero suicide target) was obtained but we were unable to access the full document (30). Although it was recognised that not every worldwide policy relating to CYP who were suicidal could be sourced, it was important to try and include all Scottish and UK wide relevant policies. A request to the Scottish Government asking them to detail policies that should be included in the review, identified one further policy that had not been considered (31) , and this together with a related practice guide (32) were included for screening.

Screening of the 42 full text documents was completed in full by the first reviewer (LG), with second reviewers each reviewing 5 independently (ED; MM), meaning a total of 25% was cross – validated. A meeting was then held to discuss the policy screening process, and to agree decisions about inclusion and exclusion. A total of 32 policy documents met the inclusion criteria. Reasons for exclusion were: document did not relate to or mention child suicidality; were not transferable to the UK or Scottish setting; was a review of systematic reviews; a newer version of the document is now

For peer review only - http://bmjopen.bmj.com/site/about/guidelines.xhtml

available (Supplementary table 2.). Together with the 3 published miscellaneous reviews / reports (33–35), there was a total of 35 documents identified to be included – shown in figure 1 below.

INSERT Figure 1: Search Results – PRISMA Diagram (36)

The included policy documents, ranged from: international guidance provided by the United Nations and World Health Organisation (4,37–39); national suicide prevention strategies (22,30,40–47); mental health strategies (48–53); and frameworks (54–58); to national practice guidelines detailing how children and young people who are suicidal should be assessed and treated (19,29,59–62). The organisation and classification of these documents is illustrated in figure 2 below; providing language with which to describe the policy landscape.

INSERT FIGURE 2.

Data extraction was completed by LG using a predefined template (Supplementary document 3.) to collate key information about each of the documents including its aims and objectives, and how it related to the review question. A second reviewer independently extracted data for a sample of 25%. All three reviewers then met to discuss the process, and outcomes. There was some variation in the verbatim content extracted. This was regarded as a reflection of – (1) the size of the policy documents, and (2) because there were so few direct references to suicidal children, other content that could be interpreted as applicable but did not specifically mention suicide was also extracted from some to give context. The data extracted from each of the included policy documents were then tabulated (Supplementary document 4), categorised first by policy type, and then alphabetically by the country.

International Guidance

For peer review only - http://bmjopen.bmj.com/site/about/guidelines.xhtml

The UN 2030 Agenda for sustainable development (37) details the goals and action plan that all countries in the United Nations have agreed to deliver. Although it does not specifically mention suicidal children and young people, reducing mortality by non-communicable illness (Goal 3.4) means that reducing suicide, which as a leading cause of death, must be a priority.

The WHO mental health action plan 2013 -20 (1), set a target that all countries should work towards reducing suicide rates by 10% by 2020. It recommended that countries adopt a life-course approach to mental health, reflecting an understanding of the impact of key stages in people's lives upon health outcomes across their life span (63,64). It promotes that countries create national policies and strategies to tackle suicide prevention prioritising at risk groups including "youth". However, other than the identification of "youth" as a priority group it does not provide any other guidance on how countries should address suicidal children and young people specifically.

Two other included documents published by the WHO (4,39), although also identifying suicidal children and young people as a priority group, similarly do not go beyond this in terms of how their needs should be addressed. The WHO Mental Health Gap Action Programme (mhGAP) intervention guide provides generic guidance relating to interventions for all persons aged 10yrs and over who are suicidal, suggests suicide should be included within an assessment, and advises that if young people feel suicidal they should talk to someone they trust and return to mental health support services (39). No rationale is provided as to why 10 years of age has been selected. It does not differentiate between the assessment and treatment approach for suicidal children, and adults. Clarification on this point was sought from the WHO, but no response was received.

National policy

The World Health Organisation recommends that countries should develop suicide prevention and mental health strategies (4,38). Ten suicide prevention strategies were included in this review (22,30,40–47) , five national mental health strategies and a young person's friendly version of the Canadian mental health policy(48–53). As recommended by the WHO (38) the suicide prevention

For peer review only - http://bmjopen.bmj.com/site/about/guidelines.xhtml

strategies adopt a universal and life-course approach. They generally provide demographic background information on suicides within their country and establish why it is a priority area. The policy documents describe their government's approach to tackling suicide by detailing lists of aims, objectives and recommendations. Most strategies recognise that children and young people are a priority group for universal and targeted suicide prevention activities (For example, universal whole school-based suicide prevention programmes and generic school counselling services), however they do not differentiate between the indicated assessment and treatment offered to adults and that available to children who are suicidal. Some strategies made no reference to suicidal children and young people (22,40,46), including the Scottish Suicide Prevention Strategy 2013-2016 (46). Few mentions of therapeutic interventions specifically for children and young people who are suicidal are made in the strategies. The Irish strategy includes statements recommending that there should be early intervention, and "*enhanced support*" available (43). The New Zealand draft consultation document (44) contains suggestions that training teachers to talk to children who are suicidal, and having direct links between schools and psychologists will improve access to support for CYP who are suicidal. Notably New Zealand previously had a suicide prevention strategy directed specifically at 'youth' suicide (65), but they have since adopted a universal policy covering the life course (22). Similarly, other countries such as the USA previously had a strategy document that contained detailed objectives directly related to children and young people (youth) (66) and has moved towards a much more generic approach (45).

National mental health strategies were also found to take a life course approach and were concerned with mental health promotion, supporting positive mental health and well-being and service delivery for those who experience mental ill health. The ten national mental health strategies included in the review incorporate references to discrete service provision for children and young people. Beyond generic school-based approaches to promoting positive mental health and well-being, there was also a focus on early intervention and easier access to child and adolescent mental health services.

For peer review only - http://bmjopen.bmj.com/site/about/guidelines.xhtml

Although most strategies refer to suicide as being a priority area in mental health provision; the Irish national mental health strategy (51) is the only one that specifically mentions suicidal children and young people. Within a section dedicated to child and adolescent mental health under a heading "Suicide and Deliberate Self-Harm" (51), it has recommendations that all children who present with self-harm should be assessed by the child and adolescent mental health team, and if appropriate receive treatment. It contains statements recognising adolescence as being a period of increased risk of suicide, and notably conveys that service provision should be the same across the whole country.

The Canadian Mental Health Strategy: A Youth Perspective (49) was produced to allow the document to be more accessible and relevant to young people. Although it does not provide specific recommendations in relation to the treatment and care of children who are suicidal, it urges that mental health services should be more accessible to all and highlights the Thunder Bay Youth Suicide Prevention Task Force as an example. This task force comprised of 30 organisations working collectively to provide an immediate response

The UK government policy "No Health without mental health" (50) highlights the high incidence of self-harm amongst young people providing as an example that "10–13% of 15–16-year-olds have self-harmed in their lifetime". It includes a suggestion that all workers who are in contact with children and young people should be aware of the issues surrounding this, and sets reducing the numbers of people of all ages who harm themselves as a target. However, the document does not contain a definition of self-harm in the glossary and it is unclear if this suggestion includes those who attempt suicide. The policy contains no specific references to children and young people who are suicidal.

The Scottish Government Mental Health Strategy 2017-2027 (52) has a dedicated section to addressing the mental health needs of children and young people. However, there is no reference to CYP who are suicidal. In relation to suicide, the strategy includes a statement that suicide prevention remains a government priority that will be dealt with separately (the Scottish Government's Suicide

For peer review only - http://bmjopen.bmj.com/site/about/guidelines.xhtml

Prevention National Action Plan (67), which was published following conclusion of the scoping review search – see discussion).

Child and adolescent mental health services are delivered separately from adult services in many countries; consequently, there are distinct policies articulating a country's vision and aims for children and young people's mental health. There are five such policy documents (54,56–58,68) included in this review, entitled as frameworks. The term frameworks suggest they provide guidance for local authorities and those commissioning and delivering services; in the UK it also denotes that they define standards of care. These frameworks include an outline of goals to prioritise and promote the mental health and well-being of children and young people, and to deliver accessible services. None, however, specifically address children and young people who are suicidal. Although containing recommendations for improving access to services and crisis support which could be applicable to children and young people who are thinking about or have attempted suicide, this is not explicitly mentioned.

National Guidance

Another category of documents included in the review were national clinical guidance. These documents contain evidence-based recommendations for good practice. Although it is not compulsory to follow guidelines, organisations and clinicians must be aware of them and potentially justify their decision making should they choose to not implement them. Included clinical guidelines were: the UK NICE (Institute for Clinical Excellence) Guidelines for Self-Harm in over 8's: short term management and prevention of recurrence (19), Self-Harm in over 8's long term management and prevention of recurrence (61), the New Zealand document: The Assessment and Management of People at Risk of Suicide (60), the United States Preventative Task Force recommendations (29), and the American Academy of Paediatrics Guidance (62). The Irish National Standard Operating Procedure for CAMHS (69) was also included in this category, because it was a national document and specifically addressed the treatment and care needs of suicidal children and young people.

For peer review only - http://bmjopen.bmj.com/site/about/guidelines.xhtml

However, it was different to the other documents in this category because its implementation is compulsory.

The UK NICE guidelines for Self-Harm in over 8's: short term management and prevention of recurrence (19), apply to everyone over 8 years of age who presents following an incidence of self-harm, defining this as any act of self-harm regardless of intent. Therefore, these guidelines are applicable to anyone over 8 years who has attempted suicide; although, it does not differentiate between the behaviours (with or without suicidal intent) in relation to treatment. They cover the immediate period following a presentation of self-harm (48hrs). The guidelines contain recommendations that all children and young people who have self-harmed are admitted to hospital overnight in a paediatric ward (including adolescents aged over 14yrs of age, if this is their preference) and they should be assessed by a specialist in child and adolescent mental health. This assessment should be the same as that for adults but also include a holistic assessment of their family situation, education etc. The only direct mention of suicide is that it is listed as a factor to be assessed. In the document that follows from NICE, Self-Harm in over 8s long term management and prevention of recurrence (61), the same definition of self-harm (to include self-harming behaviours with suicidal intent) is provided, and although suicidal intent is mentioned in relation to assessing risk, it also warns against using risk assessment tools to assess suicide risk. This reflects the lack of evidence for their effectiveness (70,71). In the final recommendations section under "ACCESS TO SERVICES" (61), it states children and young people who self-harm should be able to access all therapies and treatments available from Child and Adolescent Mental Health Services.

The New Zealand guidelines (60) are explicitly in relation to managing (all) people at risk of suicide. This document includes statements that all persons who are suicidal should be taken seriously and has a section dedicated to the treatment of children and adolescents. It includes recommendations that risk assessment of suicidal children and young people should be conducted by someone trained in working with them, and that they should draw on information from the people around the child

For peer review only

such as family and teachers etc. as well as the child or young person themselves. In the background information provided in the document it is stated that New Zealand has one of the highest rates of suicide amongst young people.

The USA document "Screening for Suicide Risk in Adolescents, Adults, and Older Adults in Primary Care: U.S. Preventive Services Task Force Recommendation Statement" (29) includes a statement reflecting that there is no evidence to support any particular treatment or intervention for adolescents at risk of suicide, and not enough evidence to support assessment tools. The American Academy of Paediatrics however, publish very specific guidance for the treatment of adolescents presenting to primary healthcare following a suicide attempt or presenting with suicidal ideation (28).

The National Operating Procedure for CAMHS in Ireland (69) was unique in that it specifically included standards of expected care and treatment for children and young people who are suicidal across Ireland. It contained a statement that CAMHS would accept referrals for children and young people where there are suicidal behaviours, and intent. Similarly, to what was found within the other frameworks for child and adolescent mental health that were included in the review, this document (69) also included general statements about service provision that could be applied to children and young people who are suicidal, for example, references to children and young people who need an immediate response, however the term suicidal is not specifically used.

Beyond the policy documents included (international policies, national policies, and national frameworks), there were very few other reports or reviews that were identified as relevant to the review question. Responding to Self-Harm in Scotland (34), is the report from the national self-harm working group and recognises that most people who self-harm do not intend to die. It includes a statement that young people are more likely to self-harm. One of its key recommendations is that there are clear referral pathways developed for people who self-harm, but it does not suggest what this might be, and is not specific to, or does not differentiate, between child and adult populations. A

For peer review only - http://bmjopen.bmj.com/site/about/guidelines.xhtml

report commissioned in New Zealand to review the evidence on improving the outcomes for adolescents transitioning to adult services has a chapter dedicated to youth suicide (35) which aims to provide an overview of the issue and prevention strategies. This chapter includes a description of how its national suicide prevention strategy and each of its goals applies to young people. The author concludes that the actions from the strategy can be applied to young people, albeit they are not specific to this population.

DISCUSSION

This scoping review sought to answer the question: how does policy address the treatment and care needs of children and young people who were suicidal? A total of 33 policy documents and two reports were included in this scoping review. However, overall, they offer little in relation to specific policy guidance for addressing suicidality in children and young people. Suicide prevention strategies recognise that children and young people are a priority population. However, the focus of these strategies is primarily upon universal prevention approaches for children and young people, such as whole school-based mental health and well-being education programmes, or generic counselling services. Both national mental health strategies across the lifespan, and national frameworks for children and young people's mental health, provide a blueprint for delivering services that are accessible to children and young people who need them, when they need them. However, they do not specifically mention the population of children who are suicidal clearly enough to establish explicitly the care and treatment that they should be provided with. They also do not guarantee that the strategies or frameworks are delivered.

The national guidelines included within this review contain suggestions that CYP who are self-harming, or are suicidal, should be assessed by a child and adolescent mental health practitioner, and referred to CAMHS for treatment and therapeutic interventions. However, the included national

For peer review only - http://bmjopen.bmj.com/site/about/guidelines.xhtml

frameworks for child and adolescent mental health barely reference children and young people who are suicidal.

Recent research has found that even when there are national clinical guidelines recommending practice in relation to suicide intervention and treatment, clinical staff teams are not always aware of these, and implementation varies (72). This strengthens the case for countries adopting a model, like that in Ireland, where implementing the Child and Adolescent Mental Health Services SOP (69) is compulsory, and goes beyond guidelines for recommended practice.

One of the reasons for the identified paucity of policy direction in providing interventions and treatments for children and young people who are suicidal, is perhaps the lack of evidence for the effectiveness of any particular treatment approach (73,74).

It could also be argued that the function of policy is not to address the treatment and care needs of specific populations, but provide a future vision, and action plan to achieve this, that can be interpreted and disseminated within a local context. However, the lack of dialogue around children and young people who are suicidal within the documents reviewed highlights a gap in policy provision for this population. The review of the New Zealand Suicide Prevention Strategy (35) demonstrated that the generic goals set out in the strategy could be applicable to young people; however, this was not obviously apparent from the document itself. This may be true for other national suicide prevention strategies and national mental health strategies. However, by not being explicit about their relevance to children and young people who are suicidal, it could mean that the needs of this population are overlooked by the local government agencies charged with interpreting, implementing and resourcing them. It may also lead to large variations in terms of service design and delivery across different local authorities.

For peer review only - http://bmjopen.bmj.com/site/about/guidelines.xhtml

LIMITATIONS

This is the first scoping review to consider how policy addresses the needs of children and young people who are suicidal, and provides unique insight into this policy domain. However, the lack of methodological guidance for conducting policy reviews made this difficult. Whilst we can recognise some of our limitations, we have tried to be explicit in our methodology and conduct this with rigour, despite the lack of many previous systematic policy reviews to follow an agreed methodology. Additionally, the lack of any previous description of the policy landscape from this perspective, also made identifying and sourcing documents relevant to suicidal children complex. The systematic searching of primarily journal-based databases returned very few relevant documents. Searching government websites for terms such as: "child", and "young people", and "suicide", was also problematic because many of the key documents include little direct references to children and young people who are suicidal. The search for policy documents was more intuitive than anticipated, in part due to the paucity of research in this area. One of the key findings was that there seems to be a gap in policy specifically addressing this population, but this gap also contributed to the difficulty in finding relevant policies to be included.

The identification of Ireland's standard operating procedure for CAMHS (69) suggests that there may be clear protocols for child and adolescent mental health services, and practitioners available in other countries. However, these documents tend to vary between organisations and local authorities / states and were excluded from this review because they were not national. Further exploration of these local policies, or purposive searching for other international CAMHS protocols should be considered within any future policy research in this area.

Although not a prerequisite in a scoping review, triangulating screening and data extraction, supported identifying that there is little policy dialogue about indicated suicide prevention strategies for children. This lack of specific reference to the care needs and pathways for suicidal children

For peer review only - http://bmjopen.bmj.com/site/about/guidelines.xhtml

meant that the documents were open to subjective interpretation by the individual reviewers. For example, although parts of policies could be interpreted as being applicable to this population of children and young people, in attempting to extract verbatim the text that addressed them reviewers struggled to identify significant relevance.

As the review was limited to English language many of the suicide prevention policies had to be excluded, including those of the Nordic Nations who are known to have advanced mental health and suicide action plans, as they could not be translated. These countries may make their policies available in English in the future as they have with "PLAN FOR SUICIDE PREVENTION AMONG THE SÀMI PEOPLE IN NORWAY, SWEDEN, AND FINLAND" (20) and they could then be included in any future review.

The policy landscape is constantly changing and evolving. Two highly relevant documents were published following completion of the systematic literature search. The Scottish Government published an updated Suicide Prevention Strategy (75), which contains acknowledgments that "children and young people require a specific focus". Recommendations within the policy document itself remain largely at a universal prevention level, for example training teachers. The strategy clearly includes a recommendation that all children should have access to crisis support when they need it, and that it is the governments' intention to "transform" child and adolescent mental health services, having appointed a children and young people's mental health task force. However, it also contains a statement suggesting that suicide rates in children are falling, which is contrary to reports from other sources which suggest that they are increasing (76), and that rates in Scotland are higher than other parts of the UK (8). (Recent changes in coding of deaths in line with ICD -10 (77) (deaths with undetermined intent are now being recorded as suicide) has had implications upon recent suicide rates. Additionally, the Scottish Government does not publish annual suicide rates in populations of children aged under 15 years. They provide a statement that this statistic could be

For peer review only - http://bmjopen.bmj.com/site/about/guidelines.xhtml

misleadingly high for children in this "extreme" age group as a higher proportion of deaths are recorded as undetermined (78).

Another key document published latterly was the UK wide Self-harm and Suicide Competence Framework children and young people (79). This document is intended to outline the key competencies required of professionals working with children and young people who self-harm or are suicidal. Identifying that the knowledge and skills of those who support children who self-harm or are suicidal, requires different competencies to those who work with adults is undoubtedly a positive development. Within the document, it is emphasised that a person-centered approach should be taken towards CYP who have self-harmed or are suicidal, and they are treated with compassion and respect. It contains acknowledgement of the challenges in assessing suicide risk: scales and risk assessment tools have a low prediction value; and there remains a lack of evidence base for any effective interventions. However, it goes on to include a recommendation regarding best practice, based upon a review of effective self-harm interventions for children and adolescents (80), that identified positive effect in single trials of DBT (Dialectical Behavioral Therapy) and MBT (Mentalisation Behavioral Therapy). Overall, the report conveys the complexity involved in understanding the needs of children who self-harm and are suicidal and is a welcome guide to practitioners and service providers, concerned with the supervision and training needs of their workforce. However, it remains within the realm of recommendations - its application is not compulsory, and it highlights the paucity of evidence to support effective treatment models for this vulnerable population.

IMPLICATIONS FOR FUTURE RESEARCH

This review highlights the need for further research in several areas. Firstly, establishing a need for more robustly defined policy review methodology, and secondly a deeper exploration of the potential gap in policy provision for suicidal children and young people.

For peer review only - http://bmjopen.bmj.com/site/about/guidelines.xhtml

Although Scoping review methodology lends itself well to policy review, ensuring that the search strategy and identification of policy documents is reliable is complicated by variation in document formats, and titles that do not describe the issue in focus. This presents similar issues to those found when trying to identify qualitative literature for the purposes of review synthesis (81,82), and perhaps learning from developments in the field of qualitative evidence review could support development of more robust policy review methodology.

Application of discourse analysis, or interpretative policy analysis (Merry-Smith, 2014) may help to understand the meaning of the policy dialogue, as policy can in and of itself support the construction of, or denial of social issues (83). Consideration of how the problem of childhood suicidality is represented in policy documents could provide valuable insight (84) into the politics of addressing this highly sensitive subject, and the needs of these children.

Additionally, by widening the inclusion criteria in any future review to include more local policies would also provide further knowledge on how national policy is interpreted and applied at a local level. Exploring whether there are variations in interpretation, and if in fact the specific needs of suicidal CYP do get lost in translation.

IMPLICATIONS FOR POLICY & PRACTICE

Policy documents need to be written in such a way that they are careful not to exclude people and are therefore often very generic in their language. Taking a lifespan approach to mental health policy and suicide prevention strategies supports the holistic understanding that mental health is not just about the absence of illness, and that mental ill health, periods of distress and suicidality are all fluid concepts that can touch all our lives at different points. The aim of these strategies is for governments to explicate their commitment to addressing these issues, and to supporting people of all ages who are affected by them. However, this review suggests that by not specifically naming suicidal children and young people as a group that should have immediate access to services or

For peer review only - http://bmjopen.bmj.com/site/about/guidelines.xhtml

supports, and what this might look like, there lies a danger that generic policy statements are too open to interpretation. This could have implications for the local funding, commissioning and delivery of child and adolescent mental health services. Policy makers should clarify their ambitions for how the treatment and care needs of suicidal children and young people should be addressed in future policy documents.

This review provides practitioners with an overview of the international and national policy context within which they work, informing their practice and providing key knowledge. It may support their understanding of practice guidelines in relation to children and young people who are suicidal and equip them with a reference resource from which to draw upon.

CONCLUSION

This scoping review mapped key policy documents worldwide and established how they addressed the treatment and care needs of children and young people who are suicidal. Categorising these documents by; International Policy, National Policy and National Guidance, revealed that despite the assertion of children and young people being a priority target population within policy documents, their content mainly promotes the use of universal prevention strategies, and does not specifically address the treatment and care of children and young people who are suicidal. This highlights a potential gap in policy that could lead to the needs of this very vulnerable group being overlooked, and varying interpretations of how they should be provided for. National guidelines (in the UK, and New Zealand) (19,60), and Ireland's SOP for CAMHS (59) contain recommendation that children and young people who are considered to be at risk of suicide are assessed by a child and adolescent mental health practitioner, however, stop short of recommending treatments and interventions beyond this.

Declaration of Funding:

Lynne Gilmour is an ESRC funded PhD student. No other funding was sought for this review.

For peer review only - http://bmjopen.bmj.com/site/about/guidelines.xhtml

Competing Interests:

None of the review authors has any competing interest.

Authorship:

LG led this review and paper, ED & MM contributed to the development of the protocol. LG ran the searches and applied the selection criteria. MM and ED verified the selection of documents, independently screening all identified documents by title and abstract, and 50% in full text. LG completed data extraction, with MM and ED independently extracting data from 25% of included documents. LG mapped the included documents and wrote the first draft of the paper. This was reviewed and commented on by MM and ED. All authors read and approved the final manuscript.

For peer review only

REFERENCES

1. WHO. Mental health action plan 2013 - 2020 [Internet]. WHO. World Health Organization; 2013 [cited 2018 Oct 3]. Available from: http://www.who.int/mental_health/publications/action_plan/en/
2. WHO. WHO | Suicide prevention [Internet]. WHO. World Health Organization; 2018 [cited 2017 Dec 18]. Available from: http://www.who.int/mental_health/suicide-prevention/en/
3. WHO. WHO | Health policy [Internet]. WHO. World Health Organization; 2013 [cited 2018 Dec 12]. Available from: https://www.who.int/topics/health_policy/en/
4. WHO. Preventing suicide. 2014.
5. Jacob CM, Baird J, Barker M, Cooper C, Hanson M. The Importance of a Life Course Approach to Health: Chronic Disease Risk from Preconception through Adolescence and Adulthood [Internet]. [cited 2018 Dec 10]. Available from: https://www.who.int/life-course/publications/life-course-approach-to-health.pdf
6. UNICEF. Building the Future Children and the Sustainable Development Goals in Rich Countries Children in the Developed World [Internet]. 2018 [cited 2018 Dec 19]. Available from: www.unicef-irc.org
7. CNN. Japan's youth suicide rate highest in 30 years - CNN [Internet]. 2018 [cited 2018 Dec

For peer review only - http://bmjopen.bmj.com/site/about/guidelines.xhtml

19]. Available from: https://edition.cnn.com/2018/11/05/health/japan-youth-suicide-intl/index.html

8. Appleby L, Rodway C, Tham S-G, Raphael J, Kapur N, Shaw J, et al. Suicide by children and young people. National Confidential Inquiry into Suicide and Homicide by People with Mental Illness (NCISH). Manchester: University of Manchester, 2017. [Internet]. 2017. Available from: http://research.bmh.manchester.ac.uk/cmhs/research/centreforsuicideprevention/nci/reports/cyp_2017_report.pdf

9. Soole R, Kõlves K, De Leo D. Suicide in Children: A Systematic Review. Arch Suicide Res [Internet]. 2015 Jul 3 [cited 2018 Jan 10];19(3):285–304. Available from: http://www.tandfonline.com/doi/full/10.1080/13811118.2014.996694

10. WHO. Adolescence: a period needing special attention - recognizing-adolescence [Internet]. 2018 [cited 2018 Feb 5]. Available from: http://apps.who.int/adolescent/second-decade/section2/page1/recognizing-adolescence.html

11. Fortune S, Clarkson H. The role of child and adolescent mental health services in suicide prevention in New Zealand. Australas Psychiatry. 2006;

12. Cairney P. Understanding public policy : theories and issues. Palgrave Macmillan; 2012. 327 p.

13. Scott A, Guo B. For which strategies of suicide prevention is there evidence of effectiveness? DECISION SUPPORT TECHNIQUE EVIDENCE BASED PRACTICE HARM REDUCTION PREVENTIVE HEALTH SERVICES RISK REDUCTION BEHAVIOR SUICIDE – PREVENTION AND CONTROL SUICIDE, ATTEMPTED – PREVENTION AND CONTROL. 2012 [cited 2018 Mar 12]; Available from: http://www.euro.who.int/__data/assets/pdf_file/0003/168843/HEN-Suicide-Prevention-synthesis-report.pdf

14. Bennett K, Rhodes AE, Duda S, Cheung AH, Manassis K, Links P, et al. A Youth Suicide Prevention Plan for Canada: A Systematic Review of Reviews. Can J Psychiatry CanJPsychiatry [Internet]. 2015 [cited 2018 Mar 12];6060(66):245–57. Available from: www.TheCJP.ca

15. Gilmour L, Duncan E, Maxwell M. Policy addressing suicidality in children and young people: a scoping review protocol. BMJ Open [Internet]. 2018 Sep 17 [cited 2018 Oct 2];8(9):e023153. Available from: http://www.ncbi.nlm.nih.gov/pubmed/30224396

16. Scottish Government. Children (Scotland) Act 1995 [Internet]. Statute Law Database; 1995. Available from: https://www.legislation.gov.uk/ukpga/1995/36/contents

17. UN General Assembly (UNGA). United Nations Convention on the Rights of the Child (UNCRC). United Nations, Treaty Series, vol. 1577, p.3 1991.

18. American Psychiatric Association. Diagnostic and Statistical Manual of Mental Disorders [Internet]. American Psychiatric Association; 2013 [cited 2018 Dec 12]. Available from: https://psychiatryonline.org/doi/book/10.1176/appi.books.9780890425596

19. NICE. Self-harm in over 8s: short-term management and prevention of recurrence | Guidance and guidelines | NICE [Internet]. NICE; 2004 [cited 2018 Oct 3]. Available from: https://www.nice.org.uk/guidance/cg16

20. SÀMI NORWEGIAN NATIONAL ADVISORY UNIT ON MENTAL HEALTH AND SUBSTANCE ABUSE & THE SAAMI COUNCIL. PLAN FOR SUICIDE PREVENTION AMONG THE SÀMI PEOPLE IN NORWAY, SWEDEN, AND FINLAND [Internet]. NORWAY; 2017 [cited 2017 Dec 18]. Available from: http://www.saamicouncil.net/fileadmin/user_upload/Documents/Eara_dokumeanttat/Suicide_plan_EN.pdf

For peer review only

21. Australian Government Department of Health and Ageing. Living Is For Everyone (LIFE) Framework (2007). 2008. 44 p.

22. New Zealand Associate Minister of Health. The New Zealand Suicide Prevention Strategy 2006 - 2016 [Internet]. 2006. Available from: http://www.moh.govt.nz/suicideprevention

23. Stanley N, Cox P, Ashcroft J, Bailey S, Carter B, Carter Y, et al. Parental mental health and child welfare: reviews of policy and professional education [Internet]. 2009 [cited 2018 Mar 12]. Available from: https://www.scie.org.uk/publications/guides/guide30/files/FullResearchReview.pdf?res=true

24. Arksey H, O'Malley L. Scoping studies: towards a methodological framework. Int J Soc Res Methodol [Internet]. 2005 Feb [cited 2018 Oct 2];8(1):19–32. Available from: http://www.tandfonline.com/doi/abs/10.1080/1364557032000119616

25. Colquhoun HL, Levac D, O'Brien KK, Straus S, Tricco AC, Perrier L, et al. Scoping reviews: Time for clarity in definition, methods, and reporting. Vol. 67, Journal of Clinical Epidemiology. 2014.

26. Micah D.J. Peters BHSc, MA(Q), PhD,1 Christina M. Godfrey RN PhD,2 Hanan Khalil BPharm, MPharm, PhD 3, Patricia McInerney PhD,4 Deborah Parker5 and Cassia Baldini Soares RN, MPH P. Guidance for conducting systematic scoping review. Int J Evidence-Based Healthc [Internet]. 2015 [cited 2018 Feb 9]; Available from: http://ovidsp.tx.ovid.com/sp-3.27.2b/ovidweb.cgi?WebLinkFrameset=1&S=KGFGFPBINIDDCICANCFKGHIBKKPDAA00&returnUrl=ovidweb.cgi%3F%26Full%2BText%3DL%257cS.sh.22.23%257c0%257c01787381-201509000-00005%26S%3DKGFGFPBINIDDCICANCFKGHIBKKPDAA00&directlink=http%3A%2F

27. Tricco AC, Lillie E, Zarin W, O'Brien KK, Colquhoun H, Levac D, et al. PRISMA Extension for Scoping Reviews (PRISMA-ScR): Checklist and Explanation. Ann Intern Med [Internet]. 2018 Oct 2 [cited 2018 Dec 13];169(7):467. Available from: http://annals.org/article.aspx?doi=10.7326/M18-0850

28. Chun TH, Mace SE, Katz ER. FROM THE AMERICAN ACADEMY OF PEDIATRICS Evaluation and Management of Children and Adolescents With Acute Mental Health or Behavioral Problems. Part I: Common Clinical Challenges of Patients With Mental Health and/or Behavioral Emergencies ACADEMY OF PEDIATRICS, COMMITTEE ON PEDIATRIC EMERGENCY MEDICINE, AND AMERICAN COLLEGE OF EMERGENCY PHYSICIANS, PEDIATRIC EMERGENCY MEDICINE COMMITTEE CLINICAL REPORT Guidance for the Clinician in Rendering Pediatric Care. Pediatrics [Internet]. 2016 [cited 2018 Jan 29];138(3). Available from: http://pediatrics.aappublications.org/content/pediatrics/early/2016/08/18/peds.2016-1570.full.pdf

29. LeFevre ML, U.S. Preventive Services Task Force. Screening for Suicide Risk in Adolescents, Adults, and Older Adults in Primary Care: U.S. Preventive Services Task Force Recommendation Statement. Ann Intern Med [Internet]. 2014 May 20 [cited 2018 Oct 3];160(10):719. Available from: http://www.ncbi.nlm.nih.gov/pubmed/24842417

30. Public Health Agency of Sweden. National action programme for suicide prevention [Internet]. 2008 [cited 2018 Feb 7]. Available from: https://www.folkhalsomyndigheten.se/contentassets/55a1a12d413344b099f363c252a480bb/nation-action-programme-suicide-prevention-16128.pdf

31. Scottish Government. curriculum for excellence building the curriculum 3 a framework for learning and teaching. 2008.

For peer review only - http://bmjopen.bmj.com/site/about/guidelines.xhtml

32. Scottish Government. Health and wellbeing Principles and practice What are the main purposes of learning in health and wellbeing? What does the health and wellbeing framework mean for practitioners? [Internet]. 2009 [cited 2018 Dec 12]. Available from: http://www.scotland.gov.uk/Topics/Education/Schools/HLivi/foodnutrition

33. Betts J, Thompson J. Mental Health in Northern Ireland: Overview, Strategies, Policies, Care Pathways, CAMHS and Barriers to Accessing Services [Internet]. 2017 [cited 2018 Oct 3]. Available from: http://www.niassembly.gov.uk/globalassets/documents/raise/publications/2016-2021/2017/health/0817.pdf

34. Scottish Government. Responding to Self-Harm in Scotland Final Report: Mapping Out the Next Stage of Activity in Developing Services and Health Improvement Approaches [Internet]. 2011 [cited 2018 Oct 3]. Available from: https://www.gov.scot/Publications/2011/03/17153551/1

35. Skegg K. Youth Suicide. In: The Prime Minister's Chief Science Advisor, editor. Improving the Transition Reducing Social and Psychological Morbidity During Adolescence A report from the Prime Minister's Chief Science Advisor [Internet]. Office of the Prime Minister's Science Advisory Committee; 2011 [cited 2018 Oct 3]. Available from: https://www.pmcsa.org.nz/wp-content/uploads/Improving-the-Transition-report.pdf

36. Moher D, Liberati A, Tetzlaff J, Altman DG, Group TP. Preferred Reporting Items for Systematic Reviews and Meta-Analyses: The PRISMA Statement. PLoS Med [Internet]. 2009 Jul 21 [cited 2017 Dec 12];6(7):e1000097. Available from: http://dx.plos.org/10.1371/journal.pmed.1000097

37. United Nations. Transforming our World: The 2030 Agenda for Sustainable Development .:. Sustainable Development Knowledge Platform [Internet]. 2015 [cited 2018 Oct 3]. Available from: https://sustainabledevelopment.un.org/post2015/transformingourworld/publication

38. WHO. Mental Health Action Plan 2013 - 2020 [Internet]. 2013 [cited 2018 Mar 12]. Available from: http://apps.who.int/iris/bitstream/10665/89966/1/9789241506021_eng.pdf?ua=1

39. WHO. WHO | mhGAP Intervention Guide for mental, neurological and substance use disorders in non-specialized health settings [Internet]. WHO. World Health Organization; 2016 [cited 2018 Oct 3]. Available from: http://www.who.int/mental_health/publications/mhGAP_intervention_guide/en/

40. Australian Government Department of Health. National Suicide Prevention Strategy [Internet]. 2007 [cited 2018 Oct 3]. Available from: http://www.health.gov.au/internet/publications/publishing.nsf/Content/suicide-prevention-activities-evaluation~background~national-suicide-prevention-strategy

41. HM Government Department of Health. Preventing suicide in England: Two years on. 2015;(February). Available from: www.gov.uk/dh

42. National Office for Suicide Prevention. ￼￼￼￼￼￼￼￼￼ Connecting for Life, Ireland's National Strategy to Reduce Suicide 2015-2020 [Internet]. 2015. Available from: https://www.hse.ie/eng/services/list/4/mental-health-services/connecting-for-life/national-strategy-to-reduce-suicide/

43. Department of Health. Protect Life 2: a draft strategy for suicide prevention in the north of Ireland [Internet]. 2016 [cited 2018 Dec 12]. Available from: https://www.health-ni.gov.uk/sites/default/files/consultations/health/protect-life-2-consultation.pdf

For peer review only

44. New Zealand Associate Minister of Health. A Strategy to Prevent Suicide in New Zealand: Draft for public consultation [Internet]. 2017. Available from: https://www.health.govt.nz/publication/strategy-prevent-suicide-new-zealand-draft-public-consultation

45. Surgeon General U, National Action Alliance for Suicide Prevention T. 2012 National Strategy for Suicide Prevention: GOALS AND OBJECTIVES FOR ACTION A report of the U.S. Surgeon General and of the National Action Alliance for Suicide Prevention [Internet]. 2012 [cited 2018 Oct 3]. Available from: www.actionallianceforsuicideprevention.org/NSSP

46. Scotland. Scottish Government. Suicide prevention strategy 2013-2016. [Internet]. 2013 [cited 2018 Oct 3]. 20 p. Available from: https://www.gov.scot/Publications/2013/12/7616

47. Welsh Government. Talk to me Suicide and Self Harm Prevention Strategy for Wales 2015-2020 [Internet]. 2015 [cited 2018 Dec 12]. Available from: https://gov.wales/docs/dhss/publications/150716strategyen.pdf

48. Mental Health Commission of Canada. Changing directions, changing lives: The mental health strategy for Canada [Internet]. Calgary; 2012. Available from: https://www.mentalhealthcommission.ca/sites/default/files/MHStrategy_Strategy_ENG.pdf

49. Mental Health Commission of Canada. MHS: A Youth Perspective [Internet]. 2015 [cited 2018 Oct 3]. Available from: https://www.mentalhealthcommission.ca/English/initiatives/11849/mhs-youth-perspective

50. HM Government. No Health Without Mental Health: A cross-government mental health outcomes strategy for people of all ages. Policy. 2011.

51. DOH. A Vision for Change [Internet]. 2006 [cited 2018 Oct 3]. Available from: https://www.hse.ie/eng/services/publications/mentalhealth/visionforchange.html

52. Scottish Government. Mental health strategy : 2017-2027. [Internet]. 2017 [cited 2018 Oct 3]. 37 p. Available from: https://www.gov.scot/Publications/2017/03/1750

53. Welsh Government. Together for Mental Health Delivery Plan 2016-19 [Internet]. 2016 [cited 2018 Oct 3]. Available from: http://gov.wales/topics/people-and-communities/people/future-generations-bill/

54. Mental Health Commission Canada. Evergreen: A Child and Youth Mental Health Framework for Canada [Internet]. 2010 [cited 2018 Oct 3]. Available from: https://www.mentalhealthcommission.ca/sites/default/files/C%252526Y_Evergreen_Framework_ENG_1.pdf

55. Department of Health. Core Standards Change for Children-Every Child Matters National Service Framework for Children, Young People and Maternity Services [Internet]. 2004 [cited 2018 Oct 3]. Available from: http://www.dh.gov.uk/PolicyAndGuidance/HealthAndSocialCareTopics/ChildrenServices/fs/en

56. Department of Health & Department of Education. Transforming Children and Young People's Mental Health Provision: a Green Paper [Internet]. 2017 [cited 2018 Oct 3]. Available from: http://www.nationalarchives.gov.uk/doc/open-government-licence/version/3/

57. Scottish Government. The mental health of children and young people : a framework for promotion, prevention and cure. Scottish Executive; 2005.

58. Welsh Government. TOGETHER FOR CHILDREN AND YOUNG PEOPLE [Internet]. 2015 [cited

For peer review only - http://bmjopen.bmj.com/site/about/guidelines.xhtml

2018 Dec 12]. Available from: http://www.wales.nhs.uk/documents/Framework For Action.pdf

59. Department of Health. Child and Adolescent Mental Health Services Standard Operating Procedure [Internet]. 2015 [cited 2018 Oct 3]. Available from: https://www.hse.ie/eng/services/list/4/mental-health-services/camhs/camhssop.pdf

60. Ministry of Health & New Zealand Guidelines Group. Assessment and management of people at risk of suicide. 2003.

61. NICE. Self-harm in over 8s: long-term management | Guidance and guidelines | NICE. 2011 [cited 2018 Oct 3]; Available from: https://www.nice.org.uk/guidance/cg133

62. Chun TH, Mace SE, Katz ER. Evaluation and Management of Children and Adolescents With Acute Mental Health or Behavioral Problems. Part I: Common Clinical Challenges of Patients With Mental Health and/or Behavioral Emergencies CLINICAL REPORT Guidance for the Clinician in Rendering Pediatric Care. FROM Am Acad Pediatr Pediatr [Internet]. 2016 [cited 2018 Oct 3];138(3). Available from: www.aappublications.org/news

63. Jacob CM, Baird J, Barker M, Cooper C, Hanson M. The Importance of a Life Course Approach to Health: Chronic Disease Risk from Preconception through Adolescence and Adulthood [Internet]. [cited 2018 Dec 3]. Available from: http://www.who.int/life-course/publications/life-course-approach-to-health.pdf

64. WHO. Health at key stages of life-the life-course approach to public health [Internet]. 2011 [cited 2018 Dec 14]. Available from: http://www.euro.who.int/__data/assets/pdf_file/0019/140671/CorpBrochure_lifecourse_approach.pdf

65. Ministry of Health. In our hands : New Zealand Youth Suicide Prevention Strategy. 1998.

66. United States Government. Garret Lee Smith Memorial Act [Internet]. 2004. Available from: https://www.congress.gov/108/plaws/publ355/PLAW-108publ355.pdf

67. Scottish Government. Every life matters : Scotland's Suicide Prevention Action Plan. [Internet]. 2018 [cited 2018 Oct 3]. 22 p. Available from: https://www.gov.scot/Publications/2018/08/8874

68. Department for education and skills & Department of Health. Core Standards Change for Children-Every Child Matters National Service Framework for Children, Young People and Maternity Services [Internet]. 2004 [cited 2018 Dec 12]. Available from: http://www.dh.gov.uk/PolicyAndGuidance/HealthAndSocialCareTopics/ChildrenServices/fs/en

69. (HSE) HSE, Group CIP. Child and Adolescent Mental Health Services standard operating procedure. 2015 [cited 2018 Dec 17]; Available from: https://www.lenus.ie/handle/10147/558480

70. Carter G, Milner A, Mcgill K, Pirkis J, Kapur N, Spittal MJ. Predicting suicidal behaviours using clinical instruments: systematic review and meta-analysis of positive predictive values for risk scales. 2017 [cited 2017 Dec 13]; Available from: http://bjp.rcpsych.org/content/bjprcpsych/early/2017/03/02/bjp.bp.116.182717.full.pdf

71. Randall JR, Colman I, Rowe BH. A systematic review of psychometric assessment of self-harm risk in the emergency department. J Affect Disord [Internet]. 2011 [cited 2018 Mar 12];134:348–55. Available from: https://ac-els-cdn-com.ezproxy.stir.ac.uk/S0165032711002783/1-s2.0-S0165032711002783-

For peer review only - http://bmjopen.bmj.com/site/about/guidelines.xhtml

main.pdf?_tid=9515cb0b-4118-4a6b-ac7d-c83f74f8cdc7&acdnat=1520865293_a794864e011afd0ffc6cf8d379e1393f

72. Mokkenstorm J, Franx G, Gilissen R, Kerkhof A, Smit JH. Suicide Prevention Guideline Implementation in Specialist Mental Healthcare Institutions in The Netherlands. Int J Environ Res Public Health [Internet]. 2018 [cited 2018 Dec 17];15(5). Available from: http://www.ncbi.nlm.nih.gov/pubmed/29751572

73. Robinson J, Bailey E, Witt K, Stefanac N, Milner A, Currier D, et al. What Works in Youth Suicide Prevention? A Systematic Review and Meta-Analysis-NC-ND license (http://creativecommons.org/licenses/by-nc-nd/4.0/). EClinicalMedicine [Internet]. 2018 [cited 2018 Dec 19];4–5:52–91. Available from: https://doi.org/10.1016/j.eclinm.2018.10.004

74. Surgeon General U, National Action Alliance for Suicide Prevention T. 2012 National Strategy for Suicide Prevention: GOALS AND OBJECTIVES FOR ACTION A report of the U.S. Surgeon General and of the National Action Alliance for Suicide Prevention [Internet]. 2012 [cited 2018 Dec 14]. Available from: www.actionallianceforsuicideprevention.org/NSSP

75. Scottish Governmment. Scotland's Suicide Prevention Action Plan: Every Life Matters. 2018.

76. NCISH, University of Manchester. Suicide by children and young people in England [Internet]. 2016 [cited 2018 Jan 8]. Available from: https://www.hqip.org.uk/public/cms/253/625/19/559/National investigation into suicide in children and young people FINAL.pdf?realName=UxgbaD.pdf&v=0

77. National Records of Scotland Web Team . National Records of Scotland [Internet]. National Records of Scotland. National Records of Scotland; [cited 2018 Dec 19]. Available from: https://www.nrscotland.gov.uk/statistics-and-data/statistics/statistics-by-theme/vital-events/deaths/suicides/the-definition-of-the-statistics

78. Information Services Division (ISD) on behalf of ScotPHO . Suicide Scotland Overview [Internet]. 2018. Available from: https://www.scotpho.org.uk/health-wellbeing-and-disease/suicide/data/data-introduction/

79. National Collaborating Centre for Mental Health. SELF-HARM AND SUICIDE PREVENTION COMPETENCE FRAMEWORK Children and young people [Internet]. 2018 [cited 2018 Dec 19]. Available from: https://www.ucl.ac.uk/pals/sites/pals/files/self-harm_and_suicide_prevention_competence_framework_-_public_health_8th_oct_18.pdf

80. Hawton K, Witt KG, Taylor Salisbury TL, Arensman E, Gunnell D, Townsend E, et al. Interventions for self-harm in children and adolescents. Cochrane Database Syst Rev [Internet]. 2015 Dec 21 [cited 2018 Mar 12];(12):CD012013. Available from: http://www.ncbi.nlm.nih.gov/pubmed/26688129

81. Evans D. Database searches for qualitative research. J Med Libr Assoc [Internet]. 2002 Jul [cited 2017 Dec 13];90(3):290–3. Available from: http://www.ncbi.nlm.nih.gov/pubmed/12113512

82. Jones ML. Application of systematic review methods to qualitative research: practical issues. J Adv Nurs [Internet]. 2004 Nov 1 [cited 2019 Jan 28];48(3):271–8. Available from: http://doi.wiley.com/10.1111/j.1365-2648.2004.03196.x

83. Cairney P. Standing on the Shoulders of Giants: How Do We Combine the Insights of Multiple Theories in Public Policy Studies? Policy Stud J [Internet]. 2013 Feb 1 [cited 2018 Dec 12];41(1):1–21. Available from: http://doi.wiley.com/10.1111/psj.12000

84. Bacchi C. Why Study Problematizations? Making Politics Visible. Open J Polit Sci [Internet].

For peer review only - http://bmjopen.bmj.com/site/about/guidelines.xhtml

2012 Apr 26 [cited 2019 Jan 28];2(1):1–8. Available from: http://www.scirp.org/journal/doi.aspx?DOI=10.4236/ojps.2012.21001

Appendix

For peer review only

For peer review only - http://bmjopen.bmj.com/site/about/guidelines.xhtml

Figure 1: **Search Results – PRISMA Diagram (36)**

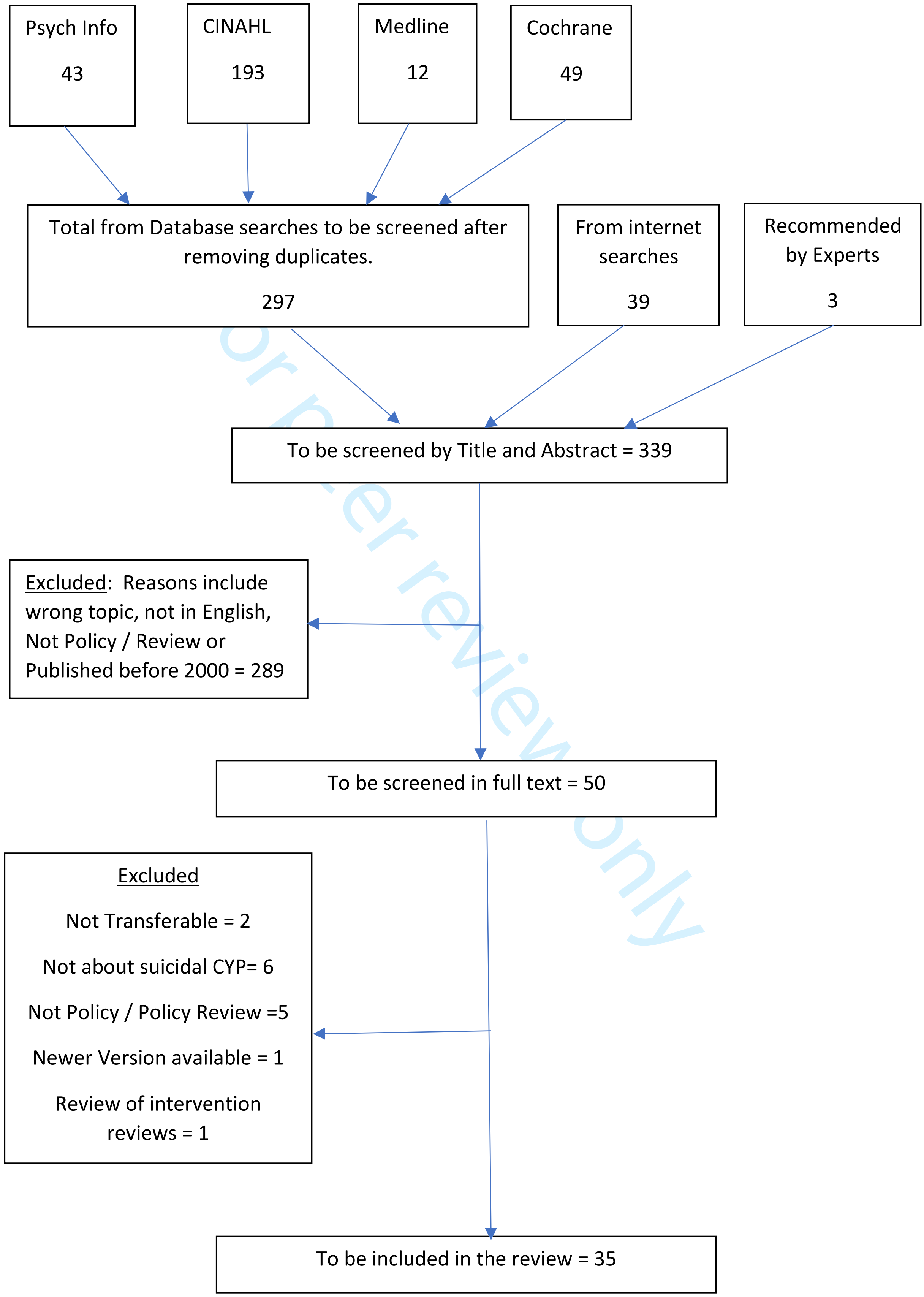

For peer review only

Figure 2.

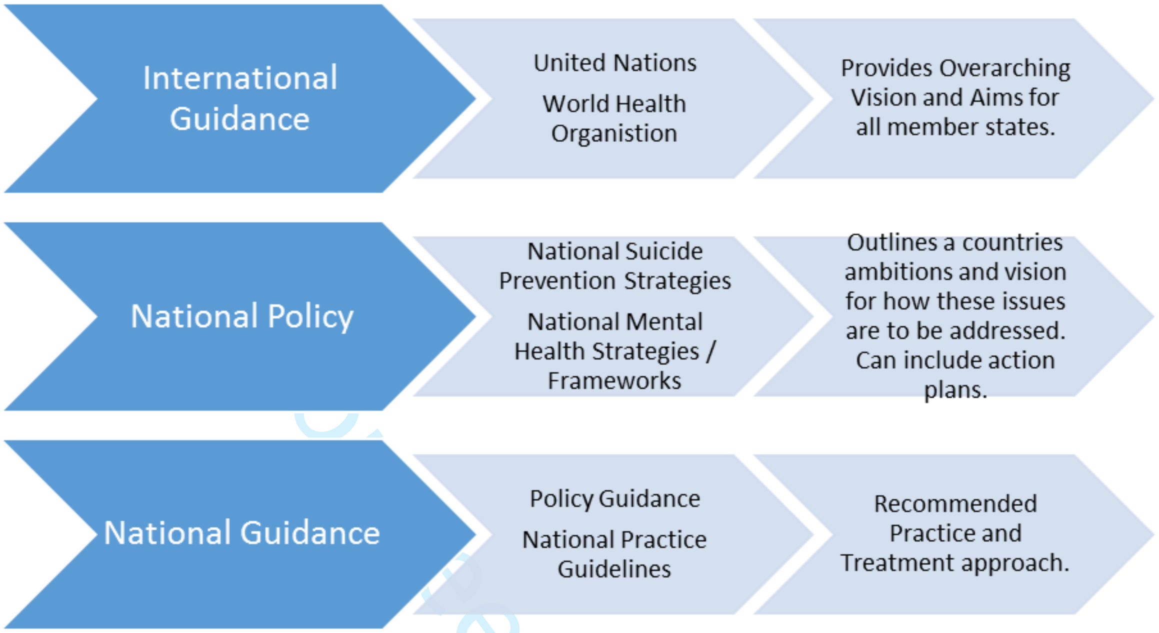
International Guidance
United Nations
World Health Organistion
Provides Overarching Vision and Aims for all member states.
National Policy
National Suicide Prevention Strategies
National Mental Health Strategies / Frameworks
Outlines a countries ambitions and vision for how these issues are to be addressed. Can include action plans.
National Guidance
Policy Guidance
National Practice Guidelines
Recommended Practice and Treatment approach.

Supplementary table 1: Mesh Terms & database search results.						
Database	SEARCH TERMS	HITS after Duplicates removed	To be Read Full Text	Need to access abstract	Rejected = Wrong Topic / Sample / Published prior to 2000	Included studies after screening in FT & Accessing Abstracts
CINAHL via EBSCO	S3 S1 AND S2 Search modes - Boolean/Phrase<br>S2 (MH "Policy Studies+") OR (MH "Public Policy+") OR (MH "Health Policy Studies") OR (MH "Policy Making") OR (MH "Health Policy+") OR "Policy" OR (MH "Hospital Policies+") OR (MH "Guideline Adherence") Limiters - English Language; Language: English; Age Groups: Child: 6-12 years, Adolescent: 13-18 years Expanders - Apply equivalent subjects; Apply related words Search modes -	193	7	9	177	2

For peer review only

Supplementary table 1: Mesh Terms & database search results.						
Database	SEARCH TERMS	HITS after Duplicates removed	To be Read Full Text	Need to access abstract	Rejected = Wrong Topic / Sample / Published prior to 2000	Included studies after screening in FT & Accessing Abstracts
	S1 (MH "Suicide+") OR (MH "Suicide, Attempted") OR (MH "Suicidal Ideation") Boolean/Phrase Limiters - English Language; Language: English; Age Groups: Child: 6-12 years, Adolescent: 13-18 years Expanders - Apply equivalent subjects; Apply related words Search modes - Boolean/Phrase					

For peer review only - http://bmjopen.bmj.com/site/about/guidelines.xhtml

Supplementary table 1: Mesh Terms & database search results.						
Database	SEARCH TERMS	HITS after Duplicates removed	To be Read Full Text	Need to access abstract	Rejected = Wrong Topic / Sample / Published prior to 2000	Included studies after screening in FT & Accessing Abstracts
COCHRANE	1. #1 "suicide" or Suicidal:ti,ab,kw (Word variations have been searched) 2. #2 child* or teen* or adolesc* or young: ti,ab,kw (Word variations have been searched) 3. #3 Policy or Guidance or Strategy or Procedure: ti,ab,kw (Word variations have been searched) 4. #4 #1 AND #2 AND #3	49 Reviews	0	0	49	0
Medline	S4 = S1 AND S2 Limiters: English<br><br>S3 = S1 AND S2<br><br>S2 = DE "Government Policy Making" OR DE "Education Policy" OR DE "Foreign Policy	12	0	0	12	0

For peer review only - http://bmjopen.bmj.com/site/about/guidelines.xhtml

Supplementary table 1: Mesh Terms & database search results.						
Database	SEARCH TERMS	HITS after Duplicates removed	To be Read Full Text	Need to access abstract	Rejected = Wrong Topic / Sample / Published prior to 2000	Included studies after screening in FT & Accessing Abstracts
	Making" OR DE "Laws" OR DE "Legislative Processes" OR DE "Older Americans Act" OR DE "Welfare Reform" OR DE "Policy Making" OR DE "Environmental Policy" OR DE "Government Policy Making" OR DE "Health Care Policy" OR DE "Health Care Policy" OR DE "Health Care Reform<br><br>S1= DE "Suicide" OR DE "Assisted Suicide" OR DE "Suicidology					
PsychInfo	TOTAL SUM of S6 and S5 after duplicates removed<br><br>S6= S1 AND S2 Narrowed by: Age (6-12yrs); Age Adolescence (13-17); Language – English<br><br>S5 = S1 AND s2 Narrowed by Age Adolescence (13-17); Language – English.<br><br>S4 = S1 AND s2<br>S3=S1 AND S2<br><br>S2 = DE "Government Policy Making" OR DE "Education Policy" OR DE "Foreign Policy Making" OR DE "Laws" OR DE "Legislative Processes" OR DE "Older Americans Act" OR	43<br><br>(13)<br><br>(44)	2 But duplicates of studies found on CINAHL.	0	41	0

4

Supplementary table 1: Mesh Terms & database search results.						
Database	SEARCH TERMS	HITS after Duplicates removed	To be Read Full Text	Need to access abstract	Rejected = Wrong Topic / Sample / Published prior to 2000	Included studies after screening in FT & Accessing Abstracts
	DE "Welfare Reform" OR DE "Policy Making" OR DE "Environmental Policy" OR DE "Government Policy Making" OR DE "Health Care Policy" OR DE "Health Care Policy" OR DE "Health Care Reform"<br><br>**S1** = DE "Suicide" OR DE "Assisted Suicide" OR DE "Suicidology"					

For peer review only - http://bmjopen.bmj.com/site/about/guidelines.xhtml

Supplementary Table 2: Reasons for Exclusion			
Source	Total number excluded	Reason for Exclusions	Number
International Policy Documents	4	Not Transferable	2
		Not about suicidal CYP	1
		A review of reviews of interventions	1
UK Policy Documents	3	Not about suicidal CYP	2
		Newer version of report available	1
Journal Articles from Databases	6	Not a policy document or review of policy	5
		Not about suicidal CYP	1
From Experts	2	Not about suicidal CYP	2

For peer review only

Supplementary Document 3: Data Extraction Template.		
	Info to be extracted	Data
Document Classification	Title of Document	
	Authors	
	Year of Publication	
	Years policy covers	
	Country	
	Is this a new strategy / document or an update of a previous version?	
	Type of Policy Document. (Is it a Policy / Strategy / Guidelines / Review?	
Policy Drivers	Policy or document purpose and Aims. (Does it include any reference to CYP suicide?)	
	Who is subject to the policy, and is adherence compulsory? (Is it aimed at clinicians or services?)	
	Does the policy cover the life-span or is it targeted at a specific age-range?	
	Is this policy universal, selective, or indicated? (WHO, 2010) Or does it include all aspects of prevention activity.	
	Does the policy relate to or feed into any other policies or programs?	
OUTCOMES	Does the policy / strategy / guidance have evaluation measures built in?	
	Who is accountable for ensuring that the policy / strategy is implemented?	
Key concepts for Review Questions	How does the policy / document relate to suicidal CYP? e.g. It is about mental health and well-being for CYP OR It is about CAMHS services OR It is about Suicide	

For peer review only

	What does the policy / document say specifically about the treatment and care of CYP who are suicidal?	
Definitions	Are key-words clearly defined and what definitions are used for self-harm for example?	

*WHO, (2010), Towards Evidence Based Practice, World Health Organisation, Western Pacific Region.

For peer review only

For peer review only - http://bmjopen.bmj.com/site/about/guidelines.xhtml

Supplementary Table 4: Mapping the policy documents							
Policy Classification	Country	Author	Document Name	Year	Summary of Document Purpose	How does the policy relate to CYP who are suicidal?	How does the policy address the treatment and care needs of CYP who are suicidal?
International Guidance.	Worldwide	United Nations	Transforming our world: The 2030 Agenda for sustainable development	2015	Sustainable development goals and action plan that all countries who are members of the united nations have agreed to implement. These goals and actions support working towards a world free from poverty, hunger and inequality, where everyone has equal human rights.	Goal 3.4: to reduce premature mortality from non-communicable diseases by one third.	It does not specifically mention children and young people who are suicidal.
	Worldwide	WHO	Mental Health Action Plan 2013-20	2013	An action plan to provide leadership and direction in the prevention, treatment and care of mental disorders, It aims to promote the human rights of	It recognises young people as being a priority group in relation to suicide prevention and intervention.	It states that countries should develop suicide prevention strategies, that give particular consideration to at risk groups – and specifies "youth".<br>It speaks to children with mental disorders having

For peer review only - http://bmjopen.bmj.com/site/about/guidelines.xhtml

					persons suffering mental ill health, and mental well-being generally.		access to psycho-social, non-medicalised treatments, but does not provide detail as to whether children who are suicidal are to be considered within this group.
	Worldwide	WHO	Preventing Suicide a Global Imperative	2014	Resource to encourage member states to develop suicide prevention strategies.	It identifies that suicide is major health issue for young people.	Provides a description of generic treatment options that may be considered helpful for all persons across the lifespan who have attempted or are thinking about suicide. (These are taken from the WHO's mhGAP Intervention Guide (2010)) It notes in relation to assessment of individuals who present as suicidal that this should be conducted in all person aged 10 years and above, but does not state why this age was selected. Otherwise there is no specific direction regarding the care and treatment of

For peer review only - http://bmjopen.bmj.com/site/about/guidelines.xhtml

							children and young people who are suicidal.
	Worldwide	WHO	mhGAP Intervention Guide for mental, neurological and substance use disorders in non-specialized health settings V2	2016	A model guide of effective and evidence based interventions to be adapted for local context.	It identifies children and adolescents as being a "special population". It does not differentiate between treatment and interventions for children and young people and adults who are suicidal. It offers assessment and intervention guidance which are the same for every-one over ten years of age. It does not state why age ten is used as a marker.	There is a module dedicated to Child & Adolescent Mental & Behavioural Disorders. Within this module there are two mentions of suicide:<br>1) Under the heading "2.5 Psychoeducation for emotional problems/disorders including depression in adolescents" (WHO, 2016, p 88) it states that if the YP notices thoughts of suicide then they should talk to someone they trust, and re-attend for mental health support.<br>2) Within a list a symptoms to assess for, when assessing for moderate to severe depression in adolescents.<br>There is a module detailing the assessment, management and follow up for people who might

For peer review only - http://bmjopen.bmj.com/site/about/guidelines.xhtml

							be at high risk of self-harming and or suicide.
National Suicide Prevention Strategies	Australia	Department of Health and Ageing – Australian Government.	Life: A Framework for prevention of Suicide in Australia	2007	National suicide prevention strategy covering the life-span.	This is an over-arching suicide prevention strategy and it does not mention CYP who are suicidal specifically. References to young people include providing support for children who are survivors of childhood abuse, and taking a whole school approach to children's mental health and wellbeing (prevention).	Not specific to children and young people who are suicidal.
	England	Department of Health	Preventing suicide in England: A cross-government outcomes strategy to save lives	2012	Sets out the government's objectives and action plan to reduce suicide rates.	Identifies CYP as a priority group. Within a section dedicated to CYP the policy gives demographic and background information about higher at-risk groups of CYP; those who have or are being bullied, children who	Mostly refers to universal and targeted prevention approaches; whole school approaches etc. and generic mental health provision. It details government approaches to tackling underlying issues such as bullying and child abuse. It does not directly address interventions and

For peer review only - http://bmjopen.bmj.com/site/about/guidelines.xhtml

						are looked after and accommodated etc.	treatments specifically for CYP who are actively suicidal other than stressing that early identification and assessment of at risk children vital.
	Ireland	National Office for Suicide Prevention	Connecting For Life: Ireland's National Strategy to Reduce Suicide 2015-2020	2015	National strategy to prevent suicide. Sets out national vision and action plan.	This policy outlines an action plan for delivering universal, targeted and integrated services for individuals who are suicidal and specifies youth services as being a priority area.	Specifically in relation to interventions for CYP who are suicidal the policy states that there should be "enhanced supports" and "enhanced availability". It recommends early intervention and psychological supports being provided and available to CYP who are at risk of suicide at both the primary care level, and secondary level including CAMHS.
	Northern Ireland	Department of Health	Protect Life 2	2016	This document is an update on the previous national suicide prevention strategy and covers the period 2016-2021. Its sets out five key aims: understanding suicidal behaviour	This strategy covers the lifespan, and includes CYP. It provides demographic information on CYP and suicide and self-harm in NI.	It notes that CYP are not a priority group for suicide prevention as the overall numbers of suicide amongst this population are low. It recognises however, that there has been an increase in the numbers of CYP who die by

For peer review only - http://bmjopen.bmj.com/site/about/guidelines.xhtml

					in NI better; improve responses to people who are suicidal; prevent suicide; support recovery; support those bereaved by suicide.		suicide and the high numbers of CYP who self-harm. In relation to treatments and care of CYP who are suicidal the strategy states that if those working with CYP are able to better identify risk factors and there is easier access to CAMHS services this would support prevention. It also outlines the role of CHILDLINE in providing support and counselling to CYP, and Crisis Intervention and Support Teams to assess children who present at hospital. It does not provide clear direction in the treatment and care of CYP who are suicidal.
	New Zealand	Associate Minister of Health	New Zealand Suicide Prevention Strategy	2006	National Strategy for suicide prevention.	This document replaces the New Zealand Youth Suicide Prevention Strategy, and moves to a generic life-span approach, although acknowledges that there may be priority	This document does not specifically address the care and treatment needs of children and young people who are suicidal. It provides a review of the literature, the context for suicide prevention, and sets

For peer review only - http://bmjopen.bmj.com/site/about/guidelines.xhtml

						groups. In a section headed "Issues relating to Age" p19, it states that young people remain a focus as they still have high rates of suicide amongst this population.	seven goals which are applicable across the life span. These goals include improved care for people who are suicidal.<br><br>Within a section headed "context" it states that although moving to a universal approach, targeted interventions for youth will still be needed.
	New Zealand	Associate Minister of Health	A Strategy to Prevent Suicide in New Zealand: A draft public consultation document.	2017	The draft framework for a new National Suicide Prevention Strategy that has three main elements. It outlines a vision (everyone will have a life worth living); a purpose (to reduce the suicide rate); to provide pathways (to increase protective factors and reduce risk).	Young People aged 15-24 are recognised as a target group.	One of the key objectives of the strategy is to recognize and support people in distress and to support people after a suicide attempt or self-harm. Of the prevention activities that are described that may be helpful that pertain specifically to CYP the focus is on improving support in the school setting: supporting teachers to respond to students who are distressed or following a suicide attempt; training

For peer review only - http://bmjopen.bmj.com/site/about/guidelines.xhtml

							teachers to talk to students who are distressed or been affected by suicidal behaviour; partnering psychologists and counsellors with schools to improve access to psychological support for CYP.
	USA	U.S. Surgeon General and the National Action Alliance for Suicide Prevention	2012 National Strategy for Suicide Prevention: GOALS AND OBJECTIVES FOR ACTION	2012	National Suicide Prevention Strategy which aims to guide suicide prevention activity.	The policy document includes suicide prevention guidance for youth, but is generic in its approach.	The policy outlines demographic information that highlights that suicidal behaviours are more common amongst young people aged 15-24yrs than adult populations. Goal 1 – includes references to school based prevention programs, but not interventions. Goal 5 - refers to the need to develop interventions for all groups of people at risk, and that these should be sensitive to and designed around the different needs of these groups. This is not

For peer review only - http://bmjopen.bmj.com/site/about/guidelines.xhtml

							specific to children and young people.
	Scotland	Scottish Government	Suicide Prevention Strategy	2013-2016	An update on previous national suicide prevention strategy (The first of which was 2003). A list of commitments and recommendations.	This document sets out the government is committed to reducing suicide and sets out a target of improving responses to people in distress.	It doesn't mention children and young people specifically.
	Sweden	Public Health Agency of Sweden	NATIONAL ACTION PROGRAMME FOR Suicide prevention	2008	This is taken form a brochure of the National Strategy, as the actual strategy is not available in English.	The national strategy has nine overall objectives, and states that their overall aim is that no-one should feel suicide is the only option they have. It recognizes young people as being a high risk group".	The brochure states that the suicide rate amongst 15-24year olds has remain unchanged, despite a reduction in the overall suicide rate over the last 15years. It does not differentiate between the treatment and care that should be given to CYP who are suicidal, although does mention under the heading "Improve medical, psychological and psychosocial initiatives" amongst a list of example initiatives some with a particular youth / student focus.
	Wales	Welsh Government	Talk to Me 2: Suicide and Self	2015	National Strategy to prevent suicide.	The strategy covers the life span, but	Demographic background information

			Harm Prevention Strategy for Wales 2015-2020		An update on a previous policy.	identifies that certain groups of CYP are priority: Looked after children, Care leavers, Children and young people in the Youth Justice System Bullied or victimised children and young people, Survivors of abuse or violence including sexual abuse and domestic violence and children and young people with a background in vulnerability.	is provided. Strategy focuses upon universal school based prevention programmes and targeted prevention activities such as school based counselling services. In relation to CYP who are suicidal it states that services should be accessible for those in crisis.
National Mental Health Strategies.	CANADA	Mental Health Commission	Changing Directions Changing Lives: The Mental Health Strategy for Canada.	2012	National Mental Health Strategy covering the lifespan.	This policy recognises suicide as being an important issue and addresses it directly. It also recommends CYP are a priority group.	Sets out objectives which include improved screening and assessment for suicide, and improved access to services. These are generic recommendations for all age groups. In relation to children and young people specifically it states that governments should address inequality in accessing to services psychotherapies

For peer review only - http://bmjopen.bmj.com/site/about/guidelines.xhtml

							and counselling. The situation is described as urgent, with families who do not have access to financial resources having to wait up to a year for treatment. This is not specific to suicidal children and young people.
	CANADA	Mental Health Commission	The Mental Health Strategy for Canada: A Youth Perspective	2015	National Mental Health Strategy written from the perspective of young people. Translates the original policy document into lay language.	Generic document – addresses mental health generally, but preventing suicide is identified as a priority. Talks about access to services and specialist help being available to people when they need it.	Not specific to the treatment and care of suicidal children and young people but states that mental health services should be more accessible to people of all ages.<br>It highlights examples of good practice in service provision and suicide prevention for children and young people. E.g. The Thunder Bay Youth Suicide Prevention Task Force (30 organisations working collaboratively to prevent suicide and provide an immediate response) & Youth advocates (Young people with lived experience).

For peer review only

							It addresses race and cultural issues in relation to suicide risk for YP: increased risk for Inuit and Metis young people. It refers to organic culturally sensitive initiatives such as The Inuit Tapirit Kanantami National Youth Council, and community health resources that offer programs of support to children and young people at risk of suicide.
	England	HMG/DH	No health without mental health A cross-government mental health outcomes strategy for people of all ages	2011	National Mental Health Strategy across the life span. Aims to address the disparity between how mental health and physical health are treated. Has six main objectives. Aims to mainstream mental health.	Adopts a lifespan approach but identifies CYP as a group with particular needs, and recommends age appropriate services and interventions.	Gives background demographic information on mental health issues including the numbers of children and young people who have self – harmed. States that all people who come into contact with CYP should be aware of the issues in relation to self-harm, as they do not always attend hospital. States that reducing suicide remains priority government target.

For peer review only - http://bmjopen.bmj.com/site/about/guidelines.xhtml

							Reduced numbers of people of all ages harming themselves is set as a measure of success of the strategy.<br><br>No specific references to CYP who are suicidal.
	Ireland	Department of Health	A vision for change	2006	Provides a vision and model for mental health service provision.	This document addresses a wide range of mental health issues and service provision. Within this there is a chapter dedicated to the provision of mental health services for CYP (Ch. 10) and a section dedicated to Suicide Prevention within Ch. 15 on Special Categories. It does not identify CYP as being a priority group.	Within Ch.10 it states that all children who present with deliberate self-harm should be assessed by the child and adolescent mental health team, and if appropriate receive treatment. Within this section it also recognises adolescence as being a period of increased risk of suicide.
	Scotland	Scottish Government	Mental Health Strategy	2017 - 2020	National Mental Health Strategy covering the life span.	The documents provides a focus on prevention and early intervention for children and young people. It includes actions relating to	It does not specifically address the treatment and care needs of CYP who are suicidal. It states that Suicide and Self-Harm continue to be

						support being available in schools, delivery of CAMHS services, and access to support and services when they are needed.	a priority area that will be addressed in a new suicide prevention strategy in 2018.
	Wales	Welsh Government	Together for Mental Health	2016	Ten year National strategy for Mental Health. Aims to improve the mental health and well-being of people of all ages in Wales adopting a life span approach to policy.	The policy addresses generic mental health needs and provision of mental health support services for CYP. It highlights the need for targeted interventions for those most at risk, and early identification and prevention.	It does not specifically address the treatment and care needs of CYP who are suicidal.
National Frameworks for Children & Adolescent Mental Health	CANADA	Mental Health Commission Canada	Evergreen: A Child and Youth Mental Health Framework for Canada	2010	Framework of values and strategic direction for the provision of mental health services for children and young people.	Policy concerned with promoting mental health and well-being amongst young people, and provision of mental health support services for CYP and families across Canada, but does not specify suicidality.	Does not mention the treatment and care of CYP who are suicidal. Lists recommendations about making services accessible (available in the evenings and weekends, accessible to YP directly, and child / youth centred), including creating “one-stop shops”, however

For peer review only - http://bmjopen.bmj.com/site/about/guidelines.xhtml

							does not specify children and young people who are suicidal. The only reference to suicide is in a direct quote by a young person talking about how there is a lack of education about mental health issues and that many Aboriginal young people are largely unaware of mental health issues but have high rates of suicide.
	England	Department of Health	National Service Framework for Children, Young People and Maternity Services: The Mental Health and Psychological Wellbeing of Children and Young People, Department of Health and Department for Education and Skills (2004) –	2004	This is part of the national framework for standards for child health services. It is the standard for children who need to access mental health and psychological services,	This standard sets out a vision for all CYP to have access to specialist mental health support services when they need it. It only directly refers to CYP who are suicidal once in relation to out of hours support.	The standard is not specific to CYP who are suicidal, however it does state that CYP should have access to support services when they need them, that they should have time to build a therapeutic relationship, and that non-attendance of clinical appointments should not be a trigger for closing cases. The standard states that services need to be creative and flexible in how they engage with CYP.

For peer review only - http://bmjopen.bmj.com/site/about/guidelines.xhtml

	England	Department of Health & Department of Education	Transforming Children and Young People's Mental Health Provision: A Green Paper	2017	Green paper proposing reform to child and adolescent mental health service provision: the appointment of mental health leads in each school; creation of support teams to asses and act as a bridge between school and CAMHS services; trial a four-week waiting time for CAMHS specialist services.	Policy update to address CYP experiencing distress and or in "crisis" being able to access the right support when they need it but does not directly refer to suicide.	Recommendations about the treatment and care of CYP experiencing distress or being in crisis could be applied to CYP who attempted or are actively thinking about suicide, but it does not specifically address suicidal CYP. Under a section entitled "Improving Crisis Care" there is reference to pilot models of crisis support underway, a new government funded scheme called "Beyond Places of Safety" for people of all ages who are in crisis, and the commissioning of the development of generic and crisis mental health pathways for CYP.
	Scotland	Scottish Government	The Mental Health of Children and Young People: A Framework for Promotion, Prevention and Care	2005 (Due to be updated)	National framework to be used as a guide for local authorities and commissioners of children's services to guide service design and delivery.	Takes a holistic view of children's mental and health and well-being and recognises the role that everyone in contact with CYP have in supporting their mental health.	No specific references to CYP who are suicidal.

For peer review only - http://bmjopen.bmj.com/site/about/guidelines.xhtml

	Wales	Welsh Government	Together for Children and Young People: Framework for Action.	2015	National Framework for child and adolescent mental health support services.	The framework provides multi agency guidance for the provision of children and young people's mental health and well-being.	It does not specifically address the treatment and care of children and young people who are suicidal. It does state that there should be a specialist CAMHS pathway, and this includes out of hours support for CYP in crisis.
Guidance Documents	Ireland	Health Service Executive & Child and Adolescent Mental Health Service.	Child and Adolescent Mental Health Services Standard Operating Procedure	2015	Provide clear direction for the delivery of CAMHS services across Ireland, to ensure consistent and transparent approach.	This document outlines the standard operating procedures for CAMHS services in Ireland, and the treatment and services that CYP and their families should expect. This includes CYP who are suicidal.	Generic statements about CAMHS services being accessible, and response times especially for situations where there may be a risk to the young person and an immediate response is required could be applicable to CYP where there is a risk of suicide but this is not clearly stated. The treatment of CYP who are suicidal is only specifically addressed in reference to the fact that CAMHS will accept referrals for CYP with "suicidal behaviours and ideation where intent is present" p15, and in terms of risk

For peer review only - http://bmjopen.bmj.com/site/about/guidelines.xhtml

							management listing risk of suicide amongst potential risk behaviours within an in-patient unit.
	New Zealand	Ministry of Health & New Zealand Guidelines Group	The Assessment and Management of People at Risk of Suicide	2003	These National Guidelines are a resource and guide for clinical staff in emergency departments, and psychiatric services working with people in mental distress who are at high risk of suicide or have attempted suicide.	This document specifically states how all persons who are suicidal should be treated and has a small section referencing the treatment of children and adolescents within it.	These guidelines provide specific recommendations on how to respond to and treat anyone who is suicidal, including children and young people. It states that anyone talking about suicide should be taken seriously. In particular to CYP it states: "Self-harm among children is rare and should be treated very seriously…The assessment of suicidal young people should be carried out by a clinician who is skilled in interviewing and working with children and adolescents whenever possible.…Risk assessments should draw on information from multiple sources, including the young

For peer review only

							person, their teachers/guidance counsellors, parents etc.” In the section on background information following the guidance it notes that New Zealand has one of the highest rates of suicide amongst young people.
	UK	NICE	Self-harm in over 8s: short-term management and prevention of recurrence	2004	Clinical guidelines for the management of self-harm in people over 8yrs in the first 48hrs following the incident of self-harm. These guidelines apply to everyone regardless of whether there is an underlying mental health illness.	The definition of Self-Harm used includes all acts of self-harm regardless of suicidal intent. These guidelines therefore apply to CYP (8-16yrs) who have attempted suicide, although this is not specifically stated.	It recommends that CYP who have self-harmed are admitted overnight to a paediatric ward (potentially adolescent ward if preferred by those aged 14yrs+), and fully assessed before discharge. The guidelines state that they should be assessed by someone trained and with experience in assessing CYP who have self-harmed. It states that assessment should be the same as for adults but also include holistic assessment of family situation etc. The only direct reference to suicide is contained

For peer review only - http://bmjopen.bmj.com/site/about/guidelines.xhtml

							within the generic description of "Assessment"; it is included in a list of factors to be assessed. It goes on to specify that child and adolescent mental health practitioners assessing CYP who have self-harmed should be trained, skilled in risk assessment, supervised regularly, and have access to senior support. Family are to be recommended to remove access to means of self-harm in the immediate aftermath of the incident.
	UK	NICE	Self-harm in over 8s: long term management and prevention of recurrence	2004	Guidelines for the long-term psychological treatment and management of self-harm. It aims to improve the quality of care that they receive, and reduce repetition of self-harm.	The definition of Self-Harm used includes all acts of self-harm regardless of suicidal intent. These guidelines therefore apply to CYP (8-16yrs) who have attempted suicide, although this is not specifically stated.	Under the heading "Risk Assessment" there are two references to suicide, which it states should be assessed in relation to risk (risk of self-harm & past and present suicidal intent). However it warns against using risk assessment tools to assess this risk.

For peer review only - http://bmjopen.bmj.com/site/about/guidelines.xhtml

							Under the heading "Access to Services" it states that children and young people who self-harm should be able to access psychological therapies and treatments from child and adolescent mental health services."<br><br>CAMHS workers who are working with CYP who self-harm should consider safeguarding procedures, and where children are referred to CAMHS under safeguarding procedures then there should be a multi-agency approach and consideration of common sense framework.<br><br>Under the heading "Primary Care" it recommends that consideration be given to referring CYP who present with self-harm to CAMHS for specialist help. Factors to consider

For peer review only

For peer review only - http://bmjopen.bmj.com/site/about/guidelines.xhtml

							are the parent or carers' distress / anxiety.
	USA	Chun et al, American Academy of Paediatrics	Evaluation and Management of Children and Adolescents With Acute Mental Health or Behavioral Problems. Part I: Common Clinical Challenges of Patients With Mental Health and/or Behavioral Emergencies	2016	To provide clinical guidance to staff in the USA primarily working in A & E departments, but for all clinicians who work with children with emotional and behavioural problems on dealing with paediatric mental health emergencies. It is not intended to be used as a standard of care and is a recommended practice as opposed to enforced.	This document has a section dedicated to dealing with children who present to the Emergency Department in hospital following a suicide attempt.	This document provides very specific recommendations on the assessment and care of children where there has been a suicide attempt. This includes medical assessment as well as suggestions for psychological assessment. It states that although there is no recognised effective risk assessment tool for suicide, it lists criteria such as not engaging in safety planning and remaining intent upon dying that if present suggest the child should be admitted to hospital for their own safety. It suggests that otherwise the child might be referred for outpatient treatment and that if this is unavailable or waiting times are long mental home providers (Patient centred care co-ordinated by their own

For peer review only - http://bmjopen.bmj.com/site/about/guidelines.xhtml

							doctor) are encouraged to provide support in the interim.
	USA	Michael L. LeFevre, MD, MSPH, on behalf of the U.S. Preventive Services Task Force*	Screening for Suicide Risk in Adolescents, Adults, and Older Adults in Primary Care: U.S. Preventive Services Task Force Recommendation Statement.	2014	Recommended clinical guidance for primary care settings in relation to the assessment of suicide risk.	Specifically addressed assessing the suicide risk in adolescents.	There is limited evidence for any treatments for adolescents at risk of suicide, and not enough evidence to support the potential benefit or harm of either screening for risk of suicide, or not.
Miscellaneous reports about policy.	Northern Ireland	Dr Jennifer Betts and Dr Janice Thompson	Mental Health in Northern Ireland: Overview, Strategies, Policies, Care Pathways, CAMHS and Barriers to Accessing Services	2017	This paper provides background information on mental health and illness in Northern Ireland (NI) including demographics on Suicide and self-harm. It details the relevant strategies and policies. It describes the care pathway for treating mental health problems, with specific reference to child and adolescent mental health	Reports on recent changes in the delivery of CAMHS services and attempts to make them accessible. Early intervention is highlighted as a priority. Provides an overview of key mental health policies and refers to objectives to reduce the number of people presenting with self-harm, and suicide prevention activity.	Gives demographic information highlighting that suicide and self-harm are issues for young people in Northern Ireland. Not specific to suicide, but it states that a referral care pathway for CAMHS is currently being developed.

For peer review only - http://bmjopen.bmj.com/site/about/guidelines.xhtml

					services (CAMHS); discussing barriers to accessing services.		
	Scotland	Scottish Government	Responding to self-harm in Scotland: Mapping out the next steps	2011	This document is a report from the National Self Harm working group who were tasked with mapping out the next stages following publication of the Truth Hurts in 2006.	This document is a recommendation for policy. It calls for improved responses to people who self-harm across the life span, and recognizes that for most people who self-harm they do not intend to die.	Provides background information and demographic information on self-harm rates in Scotland. It states that young people are more likely to self-harm. Within the proposed objectives for future actions there are recommendations for clear referral pathways for all people who self-harm but is not specific to CYP. The only direct references to the treatment and care of CYP are recommendations on guidance on confidentiality and information sharing where a child is at risk.
	New Zealand	Prime Minister's Chief Science Advisor – Ch. 16: Keren Skegg.	Improving the Transition Reducing Social and Psychological Morbidity During Adolescence	2011	This is a report commissioned by the government to review the evidence on how to improve the	Ch. 16 is devoted to Youth Suicide. It aims to give an overview of the issue and prevention strategies.	Ch.16 of the report considers how the New Zealand Suicide prevention strategy goals apply to young people. It takes each

For peer review only - http://bmjopen.bmj.com/site/about/guidelines.xhtml

					outcomes for adolescents transitioning to adulthood.		goal in turn and cogitates how it focuses on youth. In conclusion it argues that the strategy involves actions that can be applied to the issues of youth suicide. It also argues that any programmes aimed at young people must be evidence based.

For peer review only

Preferred Reporting Items for Systematic reviews and Meta-Analyses extension for Scoping Reviews (PRISMA-ScR) Checklist

SECTION	ITEM	PRISMA-ScR CHECKLIST ITEM	REPORTED ON PAGE #
**TITLE**			
Title	1	Identify the report as a scoping review.	
**ABSTRACT**			
Structured summary	2	Provide a structured summary that includes (as applicable): background, objectives, eligibility criteria, sources of evidence, charting methods, results, and conclusions that relate to the review questions and objectives.	
**INTRODUCTION**			
Rationale	3	Describe the rationale for the review in the context of what is already known. Explain why the review questions/objectives lend themselves to a scoping review approach.	
Objectives	4	Provide an explicit statement of the questions and objectives being addressed with reference to their key elements (e.g., population or participants, concepts, and context) or other relevant key elements used to conceptualize the review questions and/or objectives.	
**METHODS**			
Protocol and registration	5	Indicate whether a review protocol exists; state if and where it can be accessed (e.g., a Web address); and if available, provide registration information, including the registration number.	
Eligibility criteria	6	Specify characteristics of the sources of evidence used as eligibility criteria (e.g., years considered, language, and publication status), and provide a rationale.	
Information sources*	7	Describe all information sources in the search (e.g., databases with dates of coverage and contact with authors to identify additional sources), as well as the date the most recent search was executed.	
Search	8	Present the full electronic search strategy for at least 1 database, including any limits used, such that it could be repeated.	
Selection of sources of evidence†	9	State the process for selecting sources of evidence (i.e., screening and eligibility) included in the scoping review.	
Data charting process‡	10	Describe the methods of charting data from the included sources of evidence (e.g., calibrated forms or forms that have been tested by the team before their use, and whether data charting was done independently or in duplicate) and any processes for obtaining and confirming data from investigators.	
Data items	11	List and define all variables for which data were sought and any assumptions and simplifications made.	
Critical appraisal of individual sources of evidence§	12	If done, provide a rationale for conducting a critical appraisal of included sources of evidence; describe the methods used and how this information was used in any data synthesis (if appropriate).	
Synthesis of results	13	Describe the methods of handling and summarizing the data that were charted.	

St. Michael's
Inspired Care. Inspiring Science.

For peer review only - http://bmjopen.bmj.com/site/about/guidelines.xhtml

SECTION	ITEM	PRISMA-ScR CHECKLIST ITEM	REPORTED ON PAGE #
**RESULTS**			
Selection of sources of evidence	14	Give numbers of sources of evidence screened, assessed for eligibility, and included in the review, with reasons for exclusions at each stage, ideally using a flow diagram.	
Characteristics of sources of evidence	15	For each source of evidence, present characteristics for which data were charted and provide the citations.	
Critical appraisal within sources of evidence	16	If done, present data on critical appraisal of included sources of evidence (see item 12).	
Results of individual sources of evidence	17	For each included source of evidence, present the relevant data that were charted that relate to the review questions and objectives.	
Synthesis of results	18	Summarize and/or present the charting results as they relate to the review questions and objectives.	
**DISCUSSION**			
Summary of evidence	19	Summarize the main results (including an overview of concepts, themes, and types of evidence available), link to the review questions and objectives, and consider the relevance to key groups.	
Limitations	20	Discuss the limitations of the scoping review process.	
Conclusions	21	Provide a general interpretation of the results with respect to the review questions and objectives, as well as potential implications and/or next steps.	
**FUNDING**			
Funding	22	Describe sources of funding for the included sources of evidence, as well as sources of funding for the scoping review. Describe the role of the funders of the scoping review.	

JBI = Joanna Briggs Institute; PRISMA-ScR = Preferred Reporting Items for Systematic reviews and Meta-Analyses extension for Scoping Reviews.
* Where *sources of evidence* (see second footnote) are compiled from, such as bibliographic databases, social media platforms, and Web sites.
† A more inclusive/heterogeneous term used to account for the different types of evidence or data sources (e.g., quantitative and/or qualitative research, expert opinion, and policy documents) that may be eligible in a scoping review as opposed to only studies. This is not to be confused with *information sources* (see first footnote).
‡ The frameworks by Arksey and O'Malley (6) and Levac and colleagues (7) and the JBI guidance (4, 5) refer to the process of data extraction in a scoping review as data charting.
§ The process of systematically examining research evidence to assess its validity, results, and relevance before using it to inform a decision. This term is used for items 12 and 19 instead of "risk of bias" (which is more applicable to systematic reviews of interventions) to include and acknowledge the various sources of evidence that may be used in a scoping review (e.g., quantitative and/or qualitative research, expert opinion, and policy document).

From: Tricco AC, Lillie E, Zarin W, O'Brien KK, Colquhoun H, Levac D, et al. PRISMA Extension for Scoping Reviews (PRISMA-ScR): Checklist and Explanation. Ann Intern Med. ;169:467–473. doi: 10.7326/M18-0850

For peer review only - http://bmjopen.bmj.com/site/about/guidelines.xhtml

BMJ Open

Policy addressing suicidality in children and young people: an international scoping review.

Journal:	*BMJ Open*
Manuscript ID	bmjopen-2019-030699.R1
Article Type:	Original research
Date Submitted by the Author:	30-Aug-2019
Complete List of Authors:	Gilmour, Lynne; University of Stirling, NMAPH RU<br>Maxwell, Margaret; University of Stirling, Nursing, Midwifery and Allied Health Professions Research Unit<br>Duncan, Edward; University of Stirling, NMAHP-RU
**Primary Subject Heading**:	Health policy
Secondary Subject Heading:	Mental health, Global health, Paediatrics
Keywords:	Suicide & self-harm < PSYCHIATRY, Child & adolescent psychiatry < PSYCHIATRY, Scoping Review, Health policy < HEALTH SERVICES ADMINISTRATION & MANAGEMENT, young people

SCHOLARONE™
Manuscripts

Policy addressing suicidality in children and young people: an international scoping review.

Lynne Gilmour[1], Margaret Maxwell[1], Edward Duncan[1].

1. NMAPH Research Unit, University of Stirling, Unit 13 Scion House, Innovation Park, Stirling, FK9 4NF.

Correspondence to:

Lynne Gilmour; Email: Lyg1@stir.ac.uk ; Tel: 01786 466102.

Key-words: Suicide; policy; scoping-review; international; children; young people.

For peer review only

ABSTRACT

Objective: To map key policy documents worldwide and establish how they address the treatment and care needs of children and young people (CYP) who are suicidal.

Design: We conducted a scoping review to systematically identify relevant key policy documents following a pre-established published protocol.

Data Sources: Four databases (CINAHL; Medline; Psychinfo; The Cochrane Database of Systematic reviews) and the websites of key government, statutory and non-statutory agencies were searched. Google, and Google Scholar were used to identify other policy documents and relevant grey literature. Leading experts were consulted by email.

Eligibility criteria for selected studies: Policies, policy guidance, strategies, codes of conduct, national service frameworks, national practice guidance, white and green papers, and reviews of policy – concerned with indicated suicide prevention approaches for children up to 18yrs old. Limited by English language and published after 2000.

Data Extraction & Synthesis: Data was extracted using a predetermined template. Second reviewers independently extracted 25%. Documents were categorised as international guidance, national policy, and national guidance, and presented in a table providing a brief description of the policy, alongside how it specifically addresses suicidal CYP. Findings were further expressed using narrative synthesis.

Results: 35 policy documents were included in the review. Although many recognise children and young people as being a high risk or priority population, most don't explicitly address suicidal children and young people. In general, national guidance documents were found to convey that suicidal children should be assessed by a child and adolescent mental health practitioner but offer no clear recommendations beyond this.

Conclusion: The lack of specific reference within policy documents to the treatment and care of needs of children who are suicidal highlights a potential gap in policy that could lead to the needs of suicidal children being overlooked, and varying interpretations of appropriate responses and service provision.

For peer review only - http://bmjopen.bmj.com/site/about/guidelines.xhtml

Strengths & Limitations

- **This is the first scoping review to investigate how policy addresses suicidality in children and young people and provides new knowledge.**
- **The review authors employed a rigorous and methodological approach to the identification, screening and inclusion of policy documents, and following a clearly defined protocol.**
- **There is a lack of clear guidance in relation to how to conduct a systematic review of policy documents, which added complexity to the review process and reporting.**
- **The lack of specific reference to suicidal children within policy documents made it difficult to identify relevant documents, and there may be some we were unable to identify for inclusion.**

For peer review only

For peer review only - http://bmjopen.bmj.com/site/about/guidelines.xhtml

BACKGROUND

Suicide is a global health policy priority, with nearly 800,000 lives lost to suicide annually. Suicide is arguably preventable. Reducing suicide rates is a target of the WHO Mental Health Action Plan 2013-2020 (1) in which member states agreed to work towards reducing suicide rates by 10% by 2020 Globally, suicide prevention strategies have been established in 28 countries to date (2).

International and government policies establish the context for the direction of resources for the development and delivery of services. Health policy provides a future vision (internationally, nationally or regionally), sets priorities, and can include an action plan to achieve specific health related objectives (3). Public policies reflect international or national commitment and ambitions to address specific issues, but can vary in whether, and how they translate or relate to practice and whether there is a mandate for action.

Suicide is a leading cause of death of children and young people (CYP) worldwide, second only to accidental death (4). It is estimated that as many as 1 in 3 children in some countries have considered suicide in the past year (5). Although globally developing countries are often identified as having the highest rates of suicide overall, more economically developed countries report some of the highest rates of suicide amongst children and young people (6,7) and in some it is the main cause of death (8).

It is widely acknowledged that children and young people have different needs to adults. A retrospective review of suicides amongst children and young people found that there were even differences between the presenting issues for children under 15 years compared with adolescents (age ranges of adolescents was unspecified by the authors) (9).

Many countries commonly address the health needs of children and young people separately to adults, with discrete policy and service provision, although definitions of what age range constitutes being a child, adolescent or young person varies greatly (10). However, with most suicide prevention

For peer review only - http://bmjopen.bmj.com/site/about/guidelines.xhtml

and mental health strategies now taking a universal approach, there is the potential for the specific needs of children and young people to become lost.

Fortune and Clarkstone (11) highlight the gulf that can often exist between suicide prevention policy and practice. They argue that although policy documents in New Zealand state that every-one who is suicidal should be assessed by a trained mental health professional, services are not adequately resourced to meet the demand. This is not unique to New Zealand, or to suicide prevention policy. The overall political context and policy agenda needs to be analysed in greater depth in order to make sense of the meaning conveyed within policy documents, and attributed to them (12).

Little is known about how policy addresses suicidality in children and young people. Preliminary searches of review databases (Cochrane, DARE, JBI, and the Campbell Collection), found that there had not been a review of worldwide policy in relation to children and young people who are suicidal. Reviews to date have focused upon the effectiveness of prevention and intervention strategies (13,14). Although generating valuable knowledge on the evidence base for interventions, they do not consider the policies behind such strategies and how this shape their focus and direction.

Mapping key policy documents world-wide and identifying how they address the treatment and care needs of suicidal children and young people will: highlight best practice for how policy can influence the resourcing of services; change practice; and identify any gaps in policy provision for this vulnerable population. This knowledge will support countries who wish to develop new policies or further develop existing policies that address suicidality in children and young people. The review question, objectives, search strategy, and inclusion criteria were specified in advance and documented in a published protocol (15).

Objective:

To map key policy documents worldwide and establish how they address the treatment and care needs of children and young people who are suicidal.

For peer review only - http://bmjopen.bmj.com/site/about/guidelines.xhtml

Inclusion Criteria:

Population

The key characteristics of the population were age and suicidality, neither of which have agreed universal definitions. As mentioned in the introduction the authors recognise the disparity between the needs of children and young adults. Youth suicide research publications often tend to focus on older adolescents and young adults. Whilst appreciating the complex challenges, including transition from child to adult services faced by 16-25 year olds, this review informs a larger study, concerned specifically with a Scottish school aged population. On this basis it was agreed to adopt a definition of a child as anyone under the age of 18 years, in line with: The Children (Scotland) Act 1995 (16), and the UNCRC (United Nations Convention of the Rights of the Child, 1998) (17). Policies solely about populations aged over 18 years were excluded, however policies that cover the lifespan were included.

Suicidal behaviour is defined as acts of self-harm that result in death, as well as those with a non-fatal outcome. Non-suicidal self-injury is a term used to describe self-harming behaviour where there is no intent to die. This is most commonly used in the USA and became a discrete diagnostic category in the DSM V (18). However, in the UK and some other European countries definitions of self-harm are now also often used to include all non-fatal self-harming behaviours regardless of intent, and can include behaviour that may also be described as attempted suicide (19). As this review is concerned with identifying relevant policies for children who are suicidal (had attempted to end their own life or were thinking about suicide), it was agreed not to use the term self-harm as a search term.

Concept

Suicide prevention activities can be divided into three domains: universal activities which are aimed at everybody, including public health education programmes; selected or targeted interventions that

For peer review only - http://bmjopen.bmj.com/site/about/guidelines.xhtml

For peer review only

aim to reduce the risk amongst specific high-risk groups; and indicated interventions that may include treatments and are aimed solely at individuals presenting with suicidal behaviour. This review is solely concerned with identifying policy in relation to indicated activities, aimed at children (under 18 years of age) who are suicidal.

Context

Identified policy documents were assessed for direct relevance to Scotland and the UK, or relevant to the context and population of the UK. Policies relating to indigenous populations such as the Sami populations in Norway, Sweden and Finland, were consequently excluded (20); but generic policies in post-industrial nations with developed economies such as Australia, and New Zealand were included (21,22).

Types of Sources

Suicide prevention, like much health care policy, does not sit within clearly defined and labelled singular policy documents. As well as national suicide prevention strategies, there are more generic mental health strategies or frameworks, and national guidelines such as those published by the National Institute of Clinical Excellence (NICE) in the UK, which may contain specific references to indicated intervention approaches. Mapping policy requires a recognition of the variety of formats in which relevant documents may be found. Local government agencies and organisations also have their own individual policies and procedures, however these should reflect the national approach. It was agreed that for the purposes of this review, policy documents would include: policies, policy guidance, strategies, codes of conduct, national service frameworks, national practice guidance, and white and green papers (23). Reviews of policy documents centred on children who are suicidal were also eligible for inclusion as they contribute to the development of what is known in this area.

Given that the review aimed to map the present policy context, and most strategies are updated within a ten-year period, it was agreed to exclude any policy document or review published prior to 2000. Only those available as English Language versions were included.

For peer review only - http://bmjopen.bmj.com/site/about/guidelines.xhtml

Search Strategy

Key words to be used as search terms (Table 1.) were generated from the review question (15). Preliminary searches assisted in the refinement of these terms, and the identification of the most appropriate databases, platforms and websites. These terms were then amended for each of the databases and the exact terms, including any MESH terms and subject headings used recorded (Supplementary document 1.).

TABLE 1: Search Terms

Concept	Key-words
Children and Young People (5-18yrs)	Child*; "young people"; youth; adolesc*; teen*; paediatric
Suicide	Suicide; suicidal;
Policy	Policy; Procedure; Guidance; Strategy
LIMITS	English Language; Published after 2000.

Four databases (CINAHL; Medline; Psychinfo; The Cochrane Database of Systematic reviews) and the websites of the following key government, statutory and non-statutory agencies were searched, focusing on post-industrial nations with developed economies in order to identify those with most applicability to the UK: - for example, WHO; UNICEF, UK Government; Scottish Government; ScotPHO; NICE (UK National Institute of Health Care Excellence); National Office of Suicide Prevention (Ireland); Ministry of Health NZ; Australian Government Website; and the Mental Health Commission Canada. Google, and Google Scholar were also used to identify other policy documents and any relevant grey literature. Leading experts in the field were consulted via email.

All results were screened by title and abstract or executive summary by LG, with MM and ED screening a sample of 20%. Policy documents and articles were all screened in full by LG, and another sample of 20% was independently screened by MM and ED for inclusion. Disagreements were resolved by discussion, with the third reviewer acting as mediator.

For peer review only - http://bmjopen.bmj.com/site/about/guidelines.xhtml

METHOD OF THE REPORT

This review employed scoping review methodology to systematically identify relevant key policy documents following a pre-established search strategy and published protocol (15). Scoping review methodology and guidance first outlined by Arskey & O'Malley (24) and further developed by Levac,(25), and the Joanna Briggs Institute (26) were used to inform the methodological process. The scoping review method was chosen as it allows for the synthesis of different types of study design. Thus, lending itself to the incorporation of different policy document formats (policies, policy guidance, strategies, codes of conduct, national service frameworks, national practice guidance, and white and green papers (23), as well as any relevant existing published policy reviews. Suicidology of children and young people is a newly emerging, highly sensitive and complex area of research, therefore well suited to scoping review methods (24). The review is reported in line with the new PRISMA extension for Scoping Reviews (27). Patients and the public were not consulted as part of this scoping review as it was not appropriate or applicable.

There were 43 records retrieved from Psych-info, 193 from CINAHL, 12 from Medline and 49 from the Cochrane database of systematic reviews. After removing duplicates there was a total of 297 records to be screened by title and abstract. Separate Excel spreadsheets were set up to catalogue the lists of references from each of the databases. After screening these results by title and abstract (completed in full by LG with a second reviewer independently screening for validity) all 8 records to be screened in full text were found on CINAHL, although 2 were also found in duplicate on Psychinfo. Reasons for rejection of records included wrong topic, not in English, and published before 2000.

After screening the 8 articles identified by searching the databases (cross validated by a second reviewer) only 2 met the inclusion criteria (28,29). Five were rejected as they were not policy documents about children and young people who were suicidal, and although one seemed relevant

For peer review only - http://bmjopen.bmj.com/site/about/guidelines.xhtml

in its references to the New Zealand suicide prevention strategy (11), it was neither a policy document, nor a review of policy.

Internet searching was an iterative process, using key-words to search worldwide government, statutory and non-statutory agencies websites, with 39 potentially relevant policy documents identified. Although it is common practice in systematic reviews to screen the references of included documents for other potentially relevant papers, this occurred intuitively throughout the identification of policy documents, with one referencing others within a country. Policy documents were only included for screening if by their title and description they seemed potentially relevant.

The World Health Organisation (WHO) Mindbank database houses links to member states National Suicide Prevention Strategies, however, many were unavailable in English. The WHO mental health policy and services representative was contacted to request contact details of policy authors or country specific contacts to enquire about English language versions. From these enquiries an English language brochure outlining the content of the Swedish Suicide Prevention Strategy (known to be innovative for its zero suicide target) was obtained but we were unable to access the full document (30). Although it was recognised that not every worldwide policy relating to CYP who were suicidal could be sourced, it was important to try and include all Scottish and UK wide relevant policies. A request to the Scottish Government asking them to detail policies that should be included in the review, identified one further policy that had not been considered (31) , and this together with a related practice guide (32) were included for screening.

Screening of the 42 full text documents was completed in full by the first reviewer (LG), with second reviewers each reviewing 5 independently (ED; MM), meaning a total of 25% was cross – validated. A meeting was then held to discuss the policy screening process, and to agree decisions about inclusion and exclusion. A total of 32 policy documents met the inclusion criteria. Reasons for exclusion were: document did not relate to or mention child suicidality; were not transferable to the UK or Scottish setting; was a review of systematic reviews; a newer version of the document is now

For peer review only - http://bmjopen.bmj.com/site/about/guidelines.xhtml

For peer review only

available (Supplementary document 2.). Together with the 3 published miscellaneous reviews / reports (33–35), there was a total of 35 documents identified to be included – shown in the PRISMA diagram below (36) below (figure 1).

Patient and Public Involvement

No public were involved in this review.

RESULTS

INSERT Figure 1.

The 35 included policy documents, ranged from: international guidance provided by the United Nations and World Health Organisation (4,37–39); national suicide prevention strategies (22,30,40–47); mental health strategies (48–53); and frameworks (54–58); to national practice guidelines detailing how children and young people who are suicidal should be assessed and treated (19,29,59–62). The organisation and classification of these documents is illustrated in figure 2 below; providing language with which to describe the policy landscape.

INSERT FIGURE 2.

Data extraction was completed by LG using a predefined template (Supplementary document 3.) to collate key information about each of the documents including its aims and objectives, and how it related to the review question. A second reviewer independently extracted data for a sample of 25%. All three reviewers then met to discuss the process, and outcomes. There was some variation in the verbatim content extracted. This was regarded as a reflection of – (1) the size of the policy documents, and (2) because there were so few direct references to suicidal children, other content that could be interpreted as applicable but did not specifically mention suicide was also extracted from some to give context. The data extracted from each of the included policy documents were

For peer review only

then tabulated (Supplementary document 4), categorised first by policy type, and then alphabetically by the country.

International Guidance

The UN 2030 Agenda for sustainable development (37) details the goals and action plan that all countries in the United Nations have agreed to deliver. Although it does not specifically mention suicidal children and young people, reducing mortality by non-communicable illness (Goal 3.4) means that reducing suicide, which as a leading cause of death, must be a priority.

The WHO mental health action plan 2013 -20 (1), set a target that all countries should work towards reducing suicide rates by 10% by 2020. It recommended that countries adopt a life-course approach to mental health, reflecting an understanding of the impact of key stages in people's lives upon health outcomes across their life span (63,64). It promotes that countries create national policies and strategies to tackle suicide prevention prioritising at risk groups including "youth". However, other than the identification of "youth" as a priority group it does not provide any other guidance on how countries should address suicidal children and young people specifically.

Two other included documents published by the WHO (4,39), although also identifying suicidal children and young people as a priority group, similarly do not go beyond this in terms of how their needs should be addressed. The WHO Mental Health Gap Action Programme (mhGAP) intervention guide provides generic guidance relating to interventions for all persons aged 10yrs and over who are suicidal, suggests suicide should be included within an assessment, and advises that if young people feel suicidal they should talk to someone they trust and return to mental health support services (39). No rationale is provided as to why 10 years of age has been selected. It does not differentiate between the assessment and treatment approach for suicidal children, and adults. Clarification on this point was sought from the WHO, but no response was received.

For peer review only - http://bmjopen.bmj.com/site/about/guidelines.xhtml

National policy

The World Health Organisation recommends that countries should develop suicide prevention and mental health strategies (4,38). Ten suicide prevention strategies were included in this review (22,30,40–47) , five national mental health strategies and a young person's friendly version of the Canadian mental health policy(48–53). As recommended by the WHO (38) the suicide prevention strategies adopt a universal and life-course approach. They generally provide demographic background information on suicides within their country and establish why it is a priority area. The policy documents describe their government's approach to tackling suicide by detailing lists of aims, objectives and recommendations. Most strategies recognise that children and young people are a priority group for universal and targeted suicide prevention activities (For example, universal whole school-based suicide prevention programmes and generic school counselling services), however they do not differentiate between the indicated assessment and treatment offered to adults and that available to children who are suicidal. Some strategies made no reference to suicidal children and young people (22,40,46), including the Scottish Suicide Prevention Strategy 2013-2016 (46). Few mentions of therapeutic interventions specifically for children and young people who are suicidal are made in the strategies. The Irish strategy includes statements recommending that there should be early intervention, and "*enhanced support*" available (43). The New Zealand draft consultation document (44) contains suggestions that training teachers to talk to children who are suicidal, and having direct links between schools and psychologists will improve access to support for CYP who are suicidal. Notably New Zealand previously had a suicide prevention strategy directed specifically at 'youth' suicide (65), but they have since adopted a universal policy covering the life course (22). Similarly, other countries such as the USA previously had a strategy document that contained detailed objectives directly related to children and young people (youth) (66) and has moved towards a much more generic approach (45).

For peer review only - http://bmjopen.bmj.com/site/about/guidelines.xhtml

National mental health strategies were also found to take a life course approach and were concerned with mental health promotion, supporting positive mental health and well-being and service delivery for those who experience mental ill health. The ten national mental health strategies included in the review incorporate references to discrete service provision for children and young people. Beyond generic school-based approaches to promoting positive mental health and well-being, there was also a focus on early intervention and easier access to child and adolescent mental health services.

Although most strategies refer to suicide as being a priority area in mental health provision; the Irish national mental health strategy (51) is the only one that specifically mentions suicidal children and young people. Within a section dedicated to child and adolescent mental health under a heading "Suicide and Deliberate Self-Harm" (51), it has recommendations that all children who present with self-harm should be assessed by the child and adolescent mental health team, and if appropriate receive treatment. It contains statements recognising adolescence as being a period of increased risk of suicide, and notably conveys that service provision should be the same across the whole country.

The Canadian Mental Health Strategy: A Youth Perspective (49) was produced to allow the document to be more accessible and relevant to young people. Although it does not provide specific recommendations in relation to the treatment and care of children who are suicidal, it urges that mental health services should be more accessible to all and highlights the Thunder Bay Youth Suicide Prevention Task Force as an example. This task force comprised of 30 organisations working collectively to provide an immediate response.

The UK government policy "No Health without mental health" (50) highlights the high incidence of self-harm amongst young people providing as an example that "10–13% of 15–16-year-olds have self-harmed in their lifetime". It includes a suggestion that all workers who are in contact with children and young people should be aware of the issues surrounding this, and sets reducing the numbers of people of all ages who harm themselves as a target. However, the document does not

For peer review only - http://bmjopen.bmj.com/site/about/guidelines.xhtml

For peer review only

contain a definition of self-harm in the glossary and it is unclear if this suggestion includes those who attempt suicide. The policy contains no specific references to children and young people who are suicidal.

The Scottish Government Mental Health Strategy 2017-2027 (52) has a dedicated section to addressing the mental health needs of children and young people. However, there is no reference to children and young people who are suicidal. In relation to suicide, the strategy includes a statement that suicide prevention remains a government priority that will be dealt with separately (the Scottish Government's Suicide Prevention National Action Plan (67), which was published following conclusion of the scoping review search – see discussion).

Child and adolescent mental health services are delivered separately from adult services in many countries; consequently, there are distinct policies articulating a country's vision and aims for children and young people's mental health. There are five such policy documents (54,56–58,68) included in this review, entitled as frameworks. The term 'frameworks' suggest they provide guidance for local authorities and those commissioning and delivering services; in the UK it also denotes that they define standards of care. These frameworks include an outline of goals to prioritise and promote the mental health and well-being of children and young people, and to deliver accessible services. None, however, specifically address children and young people who are suicidal. Although containing recommendations for improving access to services and crisis support which could be applicable to children and young people who are thinking about or have attempted suicide, this is not explicitly mentioned.

National Guidance

Another category of documents included in the review were national clinical guidance. These documents contain evidence-based recommendations for good practice. Although it is not compulsory to follow guidelines, organisations and clinicians must be aware of them and potentially justify their decision making should they choose to not implement them. Included clinical guidelines

For peer review only - http://bmjopen.bmj.com/site/about/guidelines.xhtml

were: the UK NICE (Institute for Clinical Excellence) Guidelines for Self-Harm in over 8's: short term management and prevention of recurrence (19), Self-Harm in over 8's long term management and prevention of recurrence (61), the New Zealand document: The Assessment and Management of People at Risk of Suicide (60), the United States Preventative Task Force recommendations (29), and the American Academy of Paediatrics Guidance (62). The Irish National Standard Operating Procedure for CAMHS (69) was also included in this category, because it was a national document and specifically addressed the treatment and care needs of suicidal children and young people. However, it was different to the other documents in this category because its implementation is compulsory.

The UK NICE guidelines for Self-Harm in over 8's: short term management and prevention of recurrence (19), apply to everyone over 8 years of age who presents following an incidence of self-harm, defining this as any act of self-harm regardless of intent. Therefore, these guidelines are applicable to anyone over 8 years who has attempted suicide; although, it does not differentiate between the behaviours (with or without suicidal intent) in relation to treatment. They cover the immediate period following a presentation of self-harm (48hrs). The guidelines contain recommendations that all children and young people who have self-harmed are admitted to hospital overnight in a paediatric ward (including adolescents aged over 14yrs of age, if this is their preference) and they should be assessed by a specialist in child and adolescent mental health. This assessment should be the same as that for adults but also include a holistic assessment of their family situation, education etc. The only direct mention of suicide is that it is listed as a factor to be assessed. In the document that follows from NICE, Self-Harm in over 8s long term management and prevention of recurrence (61), the same definition of self-harm (to include self-harming behaviours with suicidal intent) is provided, and although suicidal intent is mentioned in relation to assessing risk, it also warns against using risk assessment tools to assess suicide risk. This reflects the lack of evidence for their effectiveness (70,71). In the final recommendations section under "Access to

For peer review only

Services" (61), it states children and young people who self-harm should be able to access all therapies and treatments available from Child and Adolescent Mental Health Services.

The New Zealand guidelines (60) are explicitly in relation to managing (all) people at risk of suicide. This document includes statements that all persons who are suicidal should be taken seriously and has a section dedicated to the treatment of children and adolescents. It includes recommendations that risk assessment of suicidal children and young people should be conducted by someone trained in working with them, and that they should draw on information from the people around the child such as family and teachers etc. as well as the child or young person themselves. In the background information provided in the document it is stated that New Zealand has one of the highest rates of suicide amongst young people.

The USA document "Screening for Suicide Risk in Adolescents, Adults, and Older Adults in Primary Care: U.S. Preventive Services Task Force Recommendation Statement" (29) includes a statement reflecting that there is no evidence to support any particular treatment or intervention for adolescents at risk of suicide, and not enough evidence to support assessment tools. The American Academy of Paediatrics however, publish very specific guidance for the treatment of adolescents presenting to primary healthcare following a suicide attempt or presenting with suicidal ideation (28).

The National Operating Procedure for CAMHS in Ireland (69) was unique in that it specifically included standards of expected care and treatment for children and young people who are suicidal across Ireland. It contained a statement that CAMHS would accept referrals for children and young people where there are suicidal behaviours, and intent. Similarly, to what was found within the other frameworks for child and adolescent mental health that were included in the review, this document (69) also included general statements about service provision that could be applied to children and young people who are suicidal, for example, references to children and young people who need an immediate response, however the term suicidal is not specifically used.

For peer review only

Beyond the policy documents included (international policies, national policies, and national frameworks), there were very few other reports or reviews that were identified as relevant to the review question. Responding to Self-Harm in Scotland (34), is the report from the national self-harm working group and recognises that most people who self-harm do not intend to die. It includes a statement that young people are more likely to self-harm. One of its key recommendations is that there are clear referral pathways developed for people who self-harm, but it does not suggest what this might be, and is not specific to, or does not differentiate, between child and adult populations. A report commissioned in New Zealand to review the evidence on improving the outcomes for adolescents transitioning to adult services has a chapter dedicated to youth suicide (35) which aims to provide an overview of the issue and prevention strategies. This chapter includes a description of how its national suicide prevention strategy and each of its goals applies to young people. The author concludes that the actions from the strategy can be applied to young people, albeit they are not specific to this population.

DISCUSSION

This scoping review sought to answer the question: how does policy address the treatment and care needs of children and young people who were suicidal? A total of 33 policy documents and two reports were included. However, overall, they offer little in relation to specific policy guidance for addressing suicidality in children and young people. Suicide prevention strategies recognise that children and young people are a priority population. However, the focus of these strategies is primarily upon universal prevention approaches for children and young people, such as whole school-based mental health and well-being education programmes, or generic counselling services. Both national mental health strategies across the lifespan, and national frameworks for children and young people's mental health, provide a blueprint for delivering services that are accessible to children and young people who need them, when they need them. However, they do not specifically mention the population of children who are suicidal clearly enough to establish explicitly the care

For peer review only - http://bmjopen.bmj.com/site/about/guidelines.xhtml

and treatment that they should be provided with. They also do not guarantee that the strategies or frameworks are delivered.

The national guidelines included within this review contain suggestions that CYP who are self-harming, or are suicidal, should be assessed by a child and adolescent mental health practitioner, and referred to CAMHS for treatment and therapeutic interventions. However, the included national frameworks for child and adolescent mental health barely reference children and young people who are suicidal.

Recent research has found that even when there are national clinical guidelines recommending practice in relation to suicide intervention and treatment, clinical staff teams are not always aware of these, and implementation varies (72). This strengthens the case for countries adopting a model, like that in Ireland, where implementing the Child and Adolescent Mental Health Services SOP (69) is compulsory, and goes beyond guidelines for recommended practice.

One of the reasons for the identified paucity of policy direction in providing interventions and treatments for children and young people who are suicidal, is perhaps the lack of evidence for the effectiveness of any particular treatment approach (73,74).

It could also be argued that the function of policy is not to address the treatment and care needs of specific populations, but provide a future vision and action plan to achieve this, that can be interpreted and disseminated within a local context. However, the lack of dialogue around children and young people who are suicidal within the documents reviewed highlights a gap in policy provision for this population. The review of the New Zealand Suicide Prevention Strategy (35) demonstrated that the generic goals set out in the strategy could be applicable to young people; however, this was not obviously apparent from the document itself. This may be true for other national suicide prevention strategies and national mental health strategies. However, by not being explicit about their relevance to children and young people who are suicidal, it could mean that the needs of this population are overlooked by the local government agencies charged with interpreting,

For peer review only - http://bmjopen.bmj.com/site/about/guidelines.xhtml

For peer review only

implementing and resourcing them. It may also lead to large variations in terms of service design and delivery across different local authorities.

LIMITATIONS

This is the first scoping review to consider how policy addresses the needs of children and young people who are suicidal, and provides unique insight into this policy domain. However, the lack of methodological guidance for conducting policy reviews made this challenging. While we recognise some of this study's limitations, we have tried to be explicit in our methodology and conduct the review with rigour. Additionally, the lack of any previous description of the suicidality policy landscape for children and young people, made identifying and sourcing relevant documents complex. The systematic searching of primarily journal-based databases returned very few relevant documents. Searching government websites for terms such as: "child", and "young people", and "suicide", was also problematic because many of the key documents include little direct references to children and young people who are suicidal. The search for policy documents was more intuitive than anticipated, in part due to the paucity of research in this area. One of the key findings was that there is a gap in policy specifically addressing this population, but this gap also contributed to the difficulty in finding relevant policies to be included.

The identification of Ireland's standard operating procedure for CAMHS (69) suggests that there may be clear protocols for child and adolescent mental health services, and practitioners available in other countries. However, these documents tend to vary between organisations and local authorities / states and were excluded from this review because they were not national. Further exploration of these local policies, or purposive searching for other international CAMHS protocols should be considered within any future policy research in this area.

Although not a prerequisite in a scoping review, triangulating screening and data extraction, helped to identify that there is little policy dialogue about indicated suicide prevention strategies for

For peer review only - http://bmjopen.bmj.com/site/about/guidelines.xhtml

children. This lack of specific reference to the care needs and pathways for suicidal children meant that the documents were open to subjective interpretation. For example, although parts of policies could be interpreted as being applicable to this population of children and young people, in attempting to extract verbatim the text that addressed them reviewers struggled to identify significant relevance.

As the review was limited to English language many of the suicide prevention policies had to be excluded, including those of the Nordic Nations who are known to have advanced mental health and suicide action plans, as they could not be translated. These countries may make their policies available in English in the future as they have with "Plan for suicide prevention among the sàmi people in Norway, Sweden, and Finland" (20) and they could then be included in a future review.

The policy landscape is constantly changing and evolving. Two highly relevant documents were published following completion of the systematic literature search. The Scottish Government published an updated Suicide Prevention Strategy (75), which contains acknowledgments that "children and young people require a specific focus". Recommendations within the policy document itself remain largely at a universal prevention level, for example training teachers. The strategy clearly includes a recommendation that all children should have access to crisis support when they need it, and that it is the governments' intention to "transform" child and adolescent mental health services, having appointed a children and young people's mental health task force. However, it also contains a statement suggesting that suicide rates in children are falling, which is contrary to reports from other sources which suggest that they are increasing (76), and that rates in Scotland are higher than other parts of the UK (8). (Recent changes in coding of deaths in line with ICD -10 (77) (deaths with undetermined intent are now being recorded as suicide) has had implications upon recent suicide rates. Additionally, the Scottish Government does not publish annual suicide rates in populations of children aged under 15 years. They provide a statement that this statistic could be

For peer review only - http://bmjopen.bmj.com/site/about/guidelines.xhtml

misleadingly high for children in this “extreme” age group as a higher proportion of deaths are recorded as undetermined (78).

Another key document published latterly was the UK wide Self-harm and Suicide Competence Framework children and young people (79). This document is intended to outline the key competencies required of professionals working with children and young people who self-harm or are suicidal. Identifying that the knowledge and skills of those who support children who self-harm or are suicidal requires different competencies to those who work with adults is undoubtedly a positive development. Within the document, it is emphasised that a person-centered approach should be taken towards CYP who have self-harmed or are suicidal, and they are treated with compassion and respect. It contains acknowledgement of the challenges in assessing suicide risk: scales and risk assessment tools have a low prediction value; and there remains a lack of evidence base for any effective interventions. However, it goes on to promote the use of DBT (Dialectical Behavioral Therapy) and MBT (Mentalisation Behavioral Therapy) as specific interventions for use by mental health professionals based on the identification of positive effect in single trials of DBT (Dialectical Behavioral Therapy) and MBT (Mentalisation Behavioral Therapy) (79: 27).This is then followed by a statement warning that the generalisability of these approaches is unknown.

Overall, the report conveys the complexity involved in understanding the needs of children who self-harm and are suicidal and is a welcome guide to practitioners and service providers, concerned with the supervision and training needs of their workforce. However, it remains within the realm of recommendations, its application is not compulsory, and it highlights the paucity of evidence to support effective treatment models for this vulnerable population.

IMPLICATIONS FOR FUTURE RESEARCH

For peer review only - http://bmjopen.bmj.com/site/about/guidelines.xhtml

This review highlights the need for further research in several areas. It establishes a need for more robustly defined policy review methodology, as well as a deeper exploration of the potential gap in policy provision for suicidal children and young people.

Although scoping review methodology lends itself well to policy review, ensuring that the search strategy and identification of policy documents is reliable is complicated by variation in document formats, and titles that do not describe the issue in focus. This presents similar issues to those found when trying to identify qualitative literature for the purposes of review synthesis (80,81), and learning from developments in the field of qualitative evidence review could support development of more robust policy review methodology.

Application of discourse analysis or interpretative policy analysis (82) may help to understand the meaning of the policy dialogue, as policy can in and of itself support the construction of, or denial of social issues (83). Consideration of how the problem of childhood suicidality is represented in policy documents could provide valuable insight (84) into the politics of addressing this highly sensitive subject, and the needs of these children.

Additionally, widening the inclusion criteria in future reviews to include more local policies would provide further knowledge on how national policy is interpreted and applied at a local level. Exploring whether there are variations in interpretation locally, and if in fact the specific needs of suicidal children and young people do get lost in translation is a knowledge gap that needs addressed.

IMPLICATIONS FOR POLICY & PRACTICE

Policy documents need to be written in such a way that they are careful not to exclude people and are therefore often very generic. Taking a lifespan approach to mental health policy and suicide prevention strategies supports the holistic understanding that mental health is not just about the absence of illness. Mental ill health, periods of distress and suicidality are all fluid concepts that can

For peer review only - http://bmjopen.bmj.com/site/about/guidelines.xhtml

touch all our lives at different points. The aim of these strategies is for governments to explicate their commitment to addressing these issues, and to supporting people of all ages who are affected by them. However, this review suggests that by not specifically naming suicidal children and young people as a group that should have immediate access to services or supports, and what this might look like, there lies a danger that generic policy statements are too open to interpretation. This could have implications for the local funding, commissioning and delivery of child and adolescent mental health services. Policy makers should clarify their ambitions for how the treatment and care needs of suicidal children and young people should be addressed in future policy documents.

This review provides practitioners with an overview of the international and national policy context within which they work, informing their practice and providing key knowledge. It may support their understanding of practice guidelines in relation to children and young people who are suicidal and equip them with a reference resource from which to draw upon.

CONCLUSION

This scoping review mapped key policy documents worldwide and established how they addressed the treatment and care needs of children and young people who are suicidal. Categorising these documents by; International Policy, National Policy and National Guidance, revealed that despite the assertion that children and young people are a priority target population within policy documents, their content mainly promotes the use of universal prevention strategies, and does not specifically address the treatment and care of children and young people who are suicidal. This highlights a potential gap in policy that could lead to the needs of this very vulnerable group being overlooked, and varying interpretations of how they should be provided for. National guidelines (in the UK, and New Zealand) (19,60), and Ireland's SOP for CAMHS (59) contain recommendations that children and young people who are considered to be at risk of suicide are assessed by a child and adolescent

For peer review only - http://bmjopen.bmj.com/site/about/guidelines.xhtml

mental health practitioner, however, stop short of recommending treatments and interventions beyond this.

Declaration of Funding:

Lynne Gilmour is an ESRC funded PhD student. No other funding was sought for this review.

Competing Interests:

None of the review authors has any competing interest.

Authorship:

LG led this review and paper, ED & MM contributed to the development of the protocol. LG ran the searches and applied the selection criteria. MM and ED verified the selection of documents, independently screening all identified documents by title and abstract, and 50% in full text. LG completed data extraction, with MM and ED independently extracting data from 25% of included documents. LG mapped the included documents and wrote the first draft of the paper. This was reviewed and commented on by MM and ED. All authors read and approved the final manuscript.

Data Sharing:

Supplementary tables are provided detailing mesh terms, reasons for exclusion, the data extraction template used, and the table of included policies.

REFERENCES

1. WHO. Mental health action plan 2013 - 2020 [Internet]. WHO. World Health Organization; 2013 [cited 2018 Oct 3]. Available from: http://www.who.int/mental_health/publications/action_plan/en/
2. WHO. WHO | Suicide prevention [Internet]. WHO. World Health Organization; 2018 [cited

For peer review only - http://bmjopen.bmj.com/site/about/guidelines.xhtml

2017 Dec 18]. Available from: http://www.who.int/mental_health/suicide-prevention/en/

3. WHO. WHO | Health policy [Internet]. WHO. World Health Organization; 2013 [cited 2018 Dec 12]. Available from: https://www.who.int/topics/health_policy/en/

4. WHO. Preventing suicide. 2014.

5. Jacob CM, Baird J, Barker M, Cooper C, Hanson M. The Importance of a Life Course Approach to Health: Chronic Disease Risk from Preconception through Adolescence and Adulthood [Internet]. [cited 2018 Dec 10]. Available from: https://www.who.int/life-course/publications/life-course-approach-to-health.pdf

6. UNICEF. Building the Future Children and the Sustainable Development Goals in Rich Countries Children in the Developed World [Internet]. 2018 [cited 2018 Dec 19]. Available from: www.unicef-irc.org

7. CNN. Japan's youth suicide rate highest in 30 years - CNN [Internet]. 2018 [cited 2018 Dec 19]. Available from: https://edition.cnn.com/2018/11/05/health/japan-youth-suicide-intl/index.html

8. Appleby L, Rodway C, Tham S-G, Raphael J, Kapur N, Shaw J, et al. Suicide by children and young people. National Confidential Inquiry into Suicide and Homicide by People with Mental Illness (NCISH). Manchester: University of Manchester, 2017. [Internet]. 2017. Available from: http://research.bmh.manchester.ac.uk/cmhs/research/centreforsuicideprevention/nci/reports/cyp_2017_report.pdf

9. Soole R, Kõlves K, De Leo D. Suicide in Children: A Systematic Review. Arch Suicide Res [Internet]. 2015 Jul 3 [cited 2018 Jan 10];19(3):285–304. Available from: http://www.tandfonline.com/doi/full/10.1080/13811118.2014.996694

10. WHO. Adolescence: a period needing special attention - recognizing-adolescence [Internet]. 2018 [cited 2018 Feb 5]. Available from: http://apps.who.int/adolescent/second-decade/section2/page1/recognizing-adolescence.html

11. Fortune S, Clarkson H. The role of child and adolescent mental health services in suicide prevention in New Zealand. Australas Psychiatry. 2006;

12. Cairney P. Understanding public policy : theories and issues. Palgrave Macmillan; 2012. 327 p.

13. Scott A, Guo B. For which strategies of suicide prevention is there evidence of effectiveness? DECISION SUPPORT TECHNIQUE EVIDENCE BASED PRACTICE HARM REDUCTION PREVENTIVE HEALTH SERVICES RISK REDUCTION BEHAVIOR SUICIDE – PREVENTION AND CONTROL SUICIDE, ATTEMPTED – PREVENTION AND CONTROL. 2012 [cited 2018 Mar 12]; Available from: http://www.euro.who.int/__data/assets/pdf_file/0003/168843/HEN-Suicide-Prevention-synthesis-report.pdf

14. Bennett K, Rhodes AE, Duda S, Cheung AH, Manassis K, Links P, et al. A Youth Suicide Prevention Plan for Canada: A Systematic Review of Reviews. Can J Psychiatry CanJPsychiatry [Internet]. 2015 [cited 2018 Mar 12];6060(66):245–57. Available from: www.TheCJP.ca

15. Gilmour L, Duncan E, Maxwell M. Policy addressing suicidality in children and young people: a scoping review protocol. BMJ Open [Internet]. 2018 Sep 17 [cited 2018 Oct 2];8(9):e023153. Available from: http://www.ncbi.nlm.nih.gov/pubmed/30224396

16. Scottish Government. Children (Scotland) Act 1995 [Internet]. Statute Law Database; 1995. Available from: https://www.legislation.gov.uk/ukpga/1995/36/contents

17. UN General Assembly (UNGA). United Nations Convention on the Rights of the Child (UNCRC).

For peer review only - http://bmjopen.bmj.com/site/about/guidelines.xhtml

United Nations, Treaty Series, vol. 1577, p.3 1991.

18. American Psychiatric Association. Diagnostic and Statistical Manual of Mental Disorders [Internet]. American Psychiatric Association; 2013 [cited 2018 Dec 12]. Available from: https://psychiatryonline.org/doi/book/10.1176/appi.books.9780890425596

19. NICE. Self-harm in over 8s: short-term management and prevention of recurrence | Guidance and guidelines | NICE [Internet]. NICE; 2004 [cited 2018 Oct 3]. Available from: https://www.nice.org.uk/guidance/cg16

20. SÀMI NORWEGIAN NATIONAL ADVISORY UNIT ON MENTAL HEALTH AND SUBSTANCE ABUSE & THE SAAMI COUNCIL. PLAN FOR SUICIDE PREVENTION AMONG THE SÀMI PEOPLE IN NORWAY, SWEDEN, AND FINLAND [Internet]. NORWAY; 2017 [cited 2017 Dec 18]. Available from: http://www.saamicouncil.net/fileadmin/user_upload/Documents/Eara_dokumeanttat/Suicide_plan_EN.pdf

21. Australian Government Department of Health and Ageing. Living Is For Everyone (LIFE) Framework (2007). 2008. 44 p.

22. New Zealand Associate Minister of Health. The New Zealand Suicide Prevention Strategy 2006 - 2016 [Internet]. 2006. Available from: http://www.moh.govt.nz/suicideprevention

23. Stanley N, Cox P, Ashcroft J, Bailey S, Carter B, Carter Y, et al. Parental mental health and child welfare: reviews of policy and professional education [Internet]. 2009 [cited 2018 Mar 12]. Available from: https://www.scie.org.uk/publications/guides/guide30/files/FullResearchReview.pdf?res=true

24. Arksey H, O'Malley L. Scoping studies: towards a methodological framework. Int J Soc Res Methodol [Internet]. 2005 Feb [cited 2018 Oct 2];8(1):19–32. Available from: http://www.tandfonline.com/doi/abs/10.1080/1364557032000119616

25. Colquhoun HL, Levac D, O'Brien KK, Straus S, Tricco AC, Perrier L, et al. Scoping reviews: Time for clarity in definition, methods, and reporting. Vol. 67, Journal of Clinical Epidemiology. 2014.

26. Micah D.J. Peters BHSc, MA(Q), PhD,1 Christina M. Godfrey RN PhD,2 Hanan Khalil BPharm, MPharm, PhD 3, Patricia McInerney PhD,4 Deborah Parker5 and Cassia Baldini Soares RN, MPH P. Guidance for conducting systematic scoping review. Int J Evidence-Based Healthc [Internet]. 2015 [cited 2018 Feb 9]; Available from: http://ovidsp.tx.ovid.com/sp-3.27.2b/ovidweb.cgi?WebLinkFrameset=1&S=KGFGFPBINIDDCICANCFKGHIBKKPDAA00&returnUrl=ovidweb.cgi%3F%26Full%2BText%3DL%257cS.sh.22.23%257c0%257c01787381-201509000-00005%26S%3DKGFGFPBINIDDCICANCFKGHIBKKPDAA00&directlink=http%3A%2F

27. Tricco AC, Lillie E, Zarin W, O'Brien KK, Colquhoun H, Levac D, et al. PRISMA Extension for Scoping Reviews (PRISMA-ScR): Checklist and Explanation. Ann Intern Med [Internet]. 2018 Oct 2 [cited 2018 Dec 13];169(7):467. Available from: http://annals.org/article.aspx?doi=10.7326/M18-0850

28. Chun TH, Mace SE, Katz ER. FROM THE AMERICAN ACADEMY OF PEDIATRICS Evaluation and Management of Children and Adolescents With Acute Mental Health or Behavioral Problems. Part I: Common Clinical Challenges of Patients With Mental Health and/or Behavioral Emergencies ACADEMY OF PEDIATRICS, COMMITTEE ON PEDIATRIC EMERGENCY MEDICINE, AND AMERICAN COLLEGE OF EMERGENCY PHYSICIANS, PEDIATRIC EMERGENCY MEDICINE COMMITTEE CLINICAL REPORT Guidance for the Clinician in Rendering Pediatric Care.

For peer review only - http://bmjopen.bmj.com/site/about/guidelines.xhtml

Pediatrics [Internet]. 2016 [cited 2018 Jan 29];138(3). Available from: http://pediatrics.aappublications.org/content/pediatrics/early/2016/08/18/peds.2016-1570.full.pdf

29. LeFevre ML, U.S. Preventive Services Task Force. Screening for Suicide Risk in Adolescents, Adults, and Older Adults in Primary Care: U.S. Preventive Services Task Force Recommendation Statement. Ann Intern Med [Internet]. 2014 May 20 [cited 2018 Oct 3];160(10):719. Available from: http://www.ncbi.nlm.nih.gov/pubmed/24842417

30. Public Health Agency of Sweden. National action programme for suicide prevention [Internet]. 2008 [cited 2018 Feb 7]. Available from: https://www.folkhalsomyndigheten.se/contentassets/55a1a12d413344b099f363c252a480bb/nation-action-programme-suicide-prevention-16128.pdf

31. Scottish Government. curriculum for excellence building the curriculum 3 a framework for learning and teaching. 2008.

32. Scottish Government. Health and wellbeing Principles and practice What are the main purposes of learning in health and wellbeing? What does the health and wellbeing framework mean for practitioners? [Internet]. 2009 [cited 2018 Dec 12]. Available from: http://www.scotland.gov.uk/Topics/Education/Schools/HLivi/foodnutrition

33. Betts J, Thompson J. Mental Health in Northern Ireland: Overview, Strategies, Policies, Care Pathways, CAMHS and Barriers to Accessing Services [Internet]. 2017 [cited 2018 Oct 3]. Available from: http://www.niassembly.gov.uk/globalassets/documents/raise/publications/2016-2021/2017/health/0817.pdf

34. Scottish Government. Responding to Self-Harm in Scotland Final Report: Mapping Out the Next Stage of Activity in Developing Services and Health Improvement Approaches [Internet]. 2011 [cited 2018 Oct 3]. Available from: https://www.gov.scot/Publications/2011/03/17153551/1

35. Skegg K. Youth Suicide. In: The Prime Minister's Chief Science Advisor, editor. Improving the Transition Reducing Social and Psychological Morbidity During Adolescence A report from the Prime Minister's Chief Science Advisor [Internet]. Office of the Prime Minister's Science Advisory Committee; 2011 [cited 2018 Oct 3]. Available from: https://www.pmcsa.org.nz/wp-content/uploads/Improving-the-Transition-report.pdf

36. Moher D, Liberati A, Tetzlaff J, Altman DG, Group TP. Preferred Reporting Items for Systematic Reviews and Meta-Analyses: The PRISMA Statement. PLoS Med [Internet]. 2009 Jul 21 [cited 2017 Dec 12];6(7):e1000097. Available from: http://dx.plos.org/10.1371/journal.pmed.1000097

37. United Nations. Transforming our World: The 2030 Agenda for Sustainable Development .:. Sustainable Development Knowledge Platform [Internet]. 2015 [cited 2018 Oct 3]. Available from: https://sustainabledevelopment.un.org/post2015/transformingourworld/publication

38. WHO. Mental Health Action Plan 2013 - 2020 [Internet]. 2013 [cited 2018 Mar 12]. Available from: http://apps.who.int/iris/bitstream/10665/89966/1/9789241506021_eng.pdf?ua=1

39. WHO. WHO | mhGAP Intervention Guide for mental, neurological and substance use disorders in non-specialized health settings [Internet]. WHO. World Health Organization; 2016 [cited 2018 Oct 3]. Available from: http://www.who.int/mental_health/publications/mhGAP_intervention_guide/en/

For peer review only

40. Australian Government Department of Health. National Suicide Prevention Strategy [Internet]. 2007 [cited 2018 Oct 3]. Available from: http://www.health.gov.au/internet/publications/publishing.nsf/Content/suicide-prevention-activities-evaluation~background~national-suicide-prevention-strategy

41. HM Government Department of Health. Preventing suicide in England: Two years on. 2015;(February). Available from: www.gov.uk/dh

42. National Office for Suicide Prevention. ￼￼￼￼￼￼￼￼￼￼ Connecting for Life, Ireland’s National Strategy to Reduce Suicide 2015-2020 [Internet]. 2015. Available from: https://www.hse.ie/eng/services/list/4/mental-health-services/connecting-for-life/national-strategy-to-reduce-suicide/

43. Department of Health. Protect Life 2: a draft strategy for suicide prevention in the north of Ireland [Internet]. 2016 [cited 2018 Dec 12]. Available from: https://www.health-ni.gov.uk/sites/default/files/consultations/health/protect-life-2-consultation.pdf

44. New Zealand Associate Minister of Health. A Strategy to Prevent Suicide in New Zealand: Draft for public consultation [Internet]. 2017. Available from: https://www.health.govt.nz/publication/strategy-prevent-suicide-new-zealand-draft-public-consultation

45. Surgeon General U, National Action Alliance for Suicide Prevention T. 2012 National Strategy for Suicide Prevention: GOALS AND OBJECTIVES FOR ACTION A report of the U.S. Surgeon General and of the National Action Alliance for Suicide Prevention [Internet]. 2012 [cited 2018 Oct 3]. Available from: www.actionallianceforsuicideprevention.org/NSSP

46. Scotland. Scottish Government. Suicide prevention strategy 2013-2016. [Internet]. 2013 [cited 2018 Oct 3]. 20 p. Available from: https://www.gov.scot/Publications/2013/12/7616

47. Welsh Government. Talk to me Suicide and Self Harm Prevention Strategy for Wales 2015-2020 [Internet]. 2015 [cited 2018 Dec 12]. Available from: https://gov.wales/docs/dhss/publications/150716strategyen.pdf

48. Mental Health Commission of Canada. Changing directions, changing lives: The mental health strategy for Canada [Internet]. Calgary; 2012. Available from: https://www.mentalhealthcommission.ca/sites/default/files/MHStrategy_Strategy_ENG.pdf

49. Mental Health Commission of Canada. MHS: A Youth Perspective [Internet]. 2015 [cited 2018 Oct 3]. Available from: https://www.mentalhealthcommission.ca/English/initiatives/11849/mhs-youth-perspective

50. HM Government. No Health Without Mental Health: A cross-government mental health outcomes strategy for people of all ages. Policy. 2011.

51. DOH. A Vision for Change [Internet]. 2006 [cited 2018 Oct 3]. Available from: https://www.hse.ie/eng/services/publications/mentalhealth/visionforchange.html

52. Scottish Government. Mental health strategy : 2017-2027. [Internet]. 2017 [cited 2018 Oct 3]. 37 p. Available from: https://www.gov.scot/Publications/2017/03/1750

53. Welsh Government. Together for Mental Health Delivery Plan 2016-19 [Internet]. 2016 [cited 2018 Oct 3]. Available from: http://gov.wales/topics/people-and-communities/people/future-generations-bill/

54. Mental Health Commission Canada. Evergreen: A Child and Youth Mental Health Framework for Canada [Internet]. 2010 [cited 2018 Oct 3]. Available from:

For peer review only

For peer review only - http://bmjopen.bmj.com/site/about/guidelines.xhtml

https://www.mentalhealthcommission.ca/sites/default/files/C%252526Y_Evergreen_Framework_ENG_1.pdf

55. Department of Health. Core Standards Change for Children-Every Child Matters National Service Framework for Children, Young People and Maternity Services [Internet]. 2004 [cited 2018 Oct 3]. Available from: http://www.dh.gov.uk/PolicyAndGuidance/HealthAndSocialCareTopics/ChildrenServices/fs/en

56. Department of Health & Department of Education. Transforming Children and Young People's Mental Health Provision: a Green Paper [Internet]. 2017 [cited 2018 Oct 3]. Available from: http://www.nationalarchives.gov.uk/doc/open-government-licence/version/3/

57. Scottish Government. The mental health of children and young people : a framework for promotion, prevention and cure. Scottish Executive; 2005.

58. Welsh Government. TOGETHER FOR CHILDREN AND YOUNG PEOPLE [Internet]. 2015 [cited 2018 Dec 12]. Available from: http://www.wales.nhs.uk/documents/Framework For Action.pdf

59. Department of Health. Child and Adolescent Mental Health Services Standard Operating Procedure [Internet]. 2015 [cited 2018 Oct 3]. Available from: https://www.hse.ie/eng/services/list/4/mental-health-services/camhs/camhssop.pdf

60. Ministry of Health & New Zealand Guidelines Group. Assessment and management of people at risk of suicide. 2003.

61. NICE. Self-harm in over 8s: long-term management | Guidance and guidelines | NICE. 2011 [cited 2018 Oct 3]; Available from: https://www.nice.org.uk/guidance/cg133

62. Chun TH, Mace SE, Katz ER. Evaluation and Management of Children and Adolescents With Acute Mental Health or Behavioral Problems. Part I: Common Clinical Challenges of Patients With Mental Health and/or Behavioral Emergencies CLINICAL REPORT Guidance for the Clinician in Rendering Pediatric Care. FROM Am Acad Pediatr Pediatr [Internet]. 2016 [cited 2018 Oct 3];138(3). Available from: www.aappublications.org/news

63. Jacob CM, Baird J, Barker M, Cooper C, Hanson M. The Importance of a Life Course Approach to Health: Chronic Disease Risk from Preconception through Adolescence and Adulthood [Internet]. [cited 2018 Dec 3]. Available from: http://www.who.int/life-course/publications/life-course-approach-to-health.pdf

64. WHO. Health at key stages of life-the life-course approach to public health [Internet]. 2011 [cited 2018 Dec 14]. Available from: http://www.euro.who.int/__data/assets/pdf_file/0019/140671/CorpBrochure_lifecourse_approach.pdf

65. Ministry of Health. In our hands : New Zealand Youth Suicide Prevention Strategy. 1998.

66. United States Government. Garret Lee Smith Memorial Act [Internet]. 2004. Available from: https://www.congress.gov/108/plaws/publ355/PLAW-108publ355.pdf

67. Scottish Government. Every life matters : Scotland's Suicide Prevention Action Plan. [Internet]. 2018 [cited 2018 Oct 3]. 22 p. Available from: https://www.gov.scot/Publications/2018/08/8874

68. Department for education and skills & Department of Health. Core Standards Change for Children-Every Child Matters National Service Framework for Children, Young People and

For peer review only

For peer review only - http://bmjopen.bmj.com/site/about/guidelines.xhtml

Maternity Services [Internet]. 2004 [cited 2018 Dec 12]. Available from: http://www.dh.gov.uk/PolicyAndGuidance/HealthAndSocialCareTopics/ChildrenServices/fs/en

69. (HSE) HSE, Group CIP. Child and Adolescent Mental Health Services standard operating procedure. 2015 [cited 2018 Dec 17]; Available from: https://www.lenus.ie/handle/10147/558480

70. Carter G, Milner A, Mcgill K, Pirkis J, Kapur N, Spittal MJ. Predicting suicidal behaviours using clinical instruments: systematic review and meta-analysis of positive predictive values for risk scales. 2017 [cited 2017 Dec 13]; Available from: http://bjp.rcpsych.org/content/bjprcpsych/early/2017/03/02/bjp.bp.116.182717.full.pdf

71. Randall JR, Colman I, Rowe BH. A systematic review of psychometric assessment of self-harm risk in the emergency department. J Affect Disord [Internet]. 2011 [cited 2018 Mar 12];134:348–55. Available from: https://ac-els-cdn-com.ezproxy.stir.ac.uk/S0165032711002783/1-s2.0-S0165032711002783-main.pdf?_tid=9515cb0b-4118-4a6b-ac7d-c83f74f8cdc7&acdnat=1520865293_a794864e011afd0ffc6cf8d379e1393f

72. Mokkenstorm J, Franx G, Gilissen R, Kerkhof A, Smit JH. Suicide Prevention Guideline Implementation in Specialist Mental Healthcare Institutions in The Netherlands. Int J Environ Res Public Health [Internet]. 2018 [cited 2018 Dec 17];15(5). Available from: http://www.ncbi.nlm.nih.gov/pubmed/29751572

73. Robinson J, Bailey E, Witt K, Stefanac N, Milner A, Currier D, et al. What Works in Youth Suicide Prevention? A Systematic Review and Meta-Analysis-NC-ND license (http://creativecommons.org/licenses/by-nc-nd/4.0/). EClinicalMedicine [Internet]. 2018 [cited 2018 Dec 19];4–5:52–91. Available from: https://doi.org/10.1016/j.eclinm.2018.10.004

74. Surgeon General U, National Action Alliance for Suicide Prevention T. 2012 National Strategy for Suicide Prevention: GOALS AND OBJECTIVES FOR ACTION A report of the U.S. Surgeon General and of the National Action Alliance for Suicide Prevention [Internet]. 2012 [cited 2018 Dec 14]. Available from: www.actionallianceforsuicideprevention.org/NSSP

75. Scottish Governmment. Scotland's Suicide Prevention Action Plan: Every Life Matters. 2018.

76. NCISH, University of Manchester. Suicide by children and young people in England [Internet]. 2016 [cited 2018 Jan 8]. Available from: https://www.hqip.org.uk/public/cms/253/625/19/559/National investigation into suicide in children and young people FINAL.pdf?realName=UxgbaD.pdf&v=0

77. National Records of Scotland Web Team . National Records of Scotland [Internet]. National Records of Scotland. National Records of Scotland; [cited 2018 Dec 19]. Available from: https://www.nrscotland.gov.uk/statistics-and-data/statistics/statistics-by-theme/vital-events/deaths/suicides/the-definition-of-the-statistics

78. Information Services Division (ISD) on behalf of ScotPHO . Suicide Scotland Overview [Internet]. 2018. Available from: https://www.scotpho.org.uk/health-wellbeing-and-disease/suicide/data/data-introduction/

79. National Collaborating Centre for Mental Health. SELF-HARM AND SUICIDE PREVENTION COMPETENCE FRAMEWORK Children and young people [Internet]. 2018 [cited 2018 Dec 19]. Available from: https://www.ucl.ac.uk/pals/sites/pals/files/self-harm_and_suicide_prevention_competence_framework_-_public_health_8th_oct_18.pdf

For peer review only

80. Evans D. Database searches for qualitative research. J Med Libr Assoc [Internet]. 2002 Jul [cited 2017 Dec 13];90(3):290–3. Available from: http://www.ncbi.nlm.nih.gov/pubmed/12113512

81. Jones ML. Application of systematic review methods to qualitative research: practical issues. J Adv Nurs [Internet]. 2004 Nov 1 [cited 2019 Jan 28];48(3):271–8. Available from: http://doi.wiley.com/10.1111/j.1365-2648.2004.03196.x

82. Smith-Merry J. Understanding talk and texts: Discourse analysis for nursing research. In: De Chesnay M, editor. Nursing Research Using Data Analysis: Qualitative Designs and Methods in Nursing. New York: Springer Publishing Company; 2014. p. 71–86.

83. Cairney P. Standing on the Shoulders of Giants: How Do We Combine the Insights of Multiple Theories in Public Policy Studies? Policy Stud J [Internet]. 2013 Feb 1 [cited 2018 Dec 12];41(1):1–21. Available from: http://doi.wiley.com/10.1111/psj.12000

84. Bacchi C. Why Study Problematizations? Making Politics Visible. Open J Polit Sci [Internet]. 2012 Apr 26 [cited 2019 Jan 28];2(1):1–8. Available from: http://www.scirp.org/journal/doi.aspx?DOI=10.4236/ojps.2012.21001

Figure Legends

Figure 1: Search Results – PRISMA Diagram

Figure 2: Policy Categories

For peer review only

Figure 1: **Search Results – PRISMA Diagram (36)**

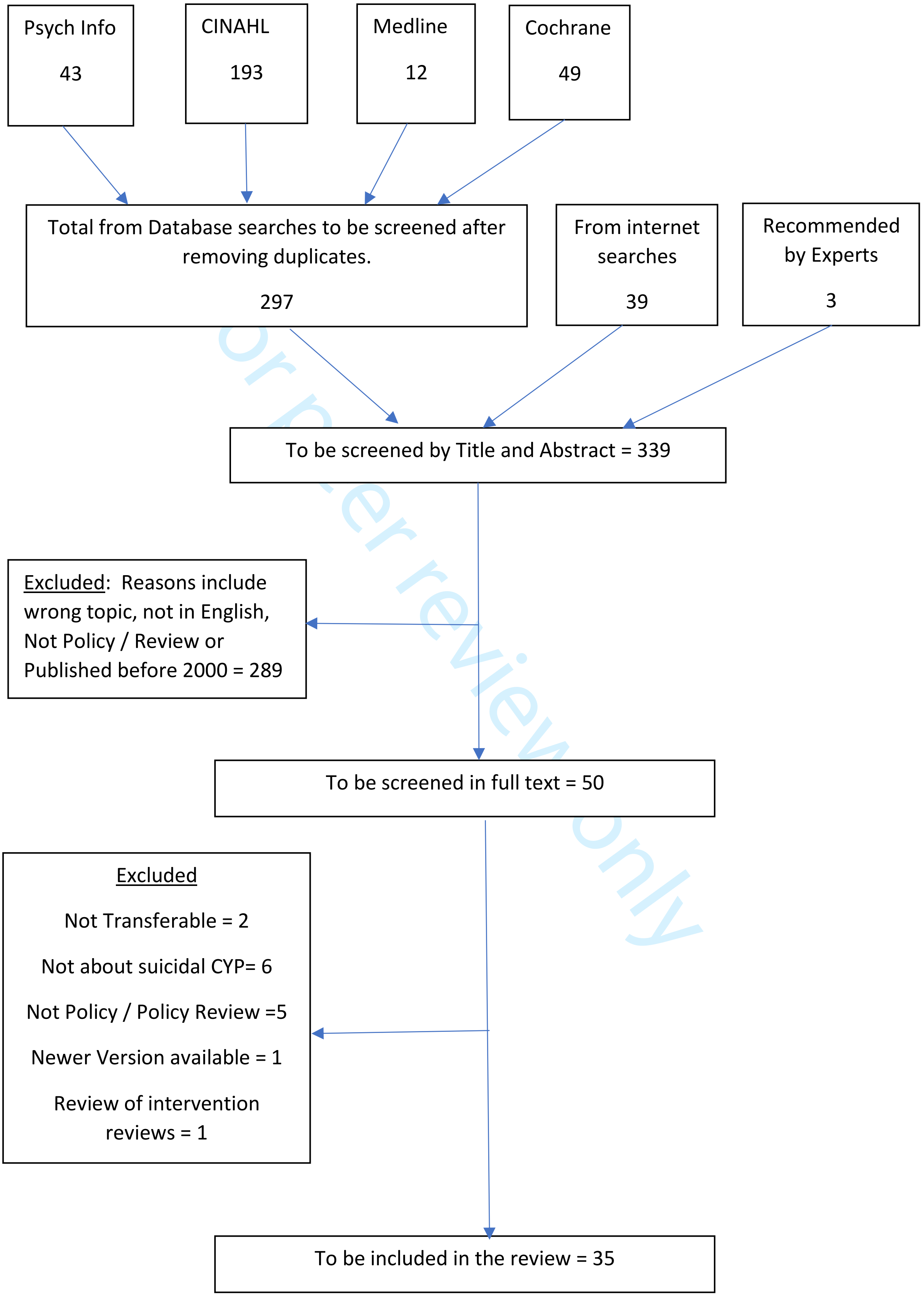

For peer review only

bmjopen-2019-030699.R1 Figure 2

Figure 2: Policy categories.

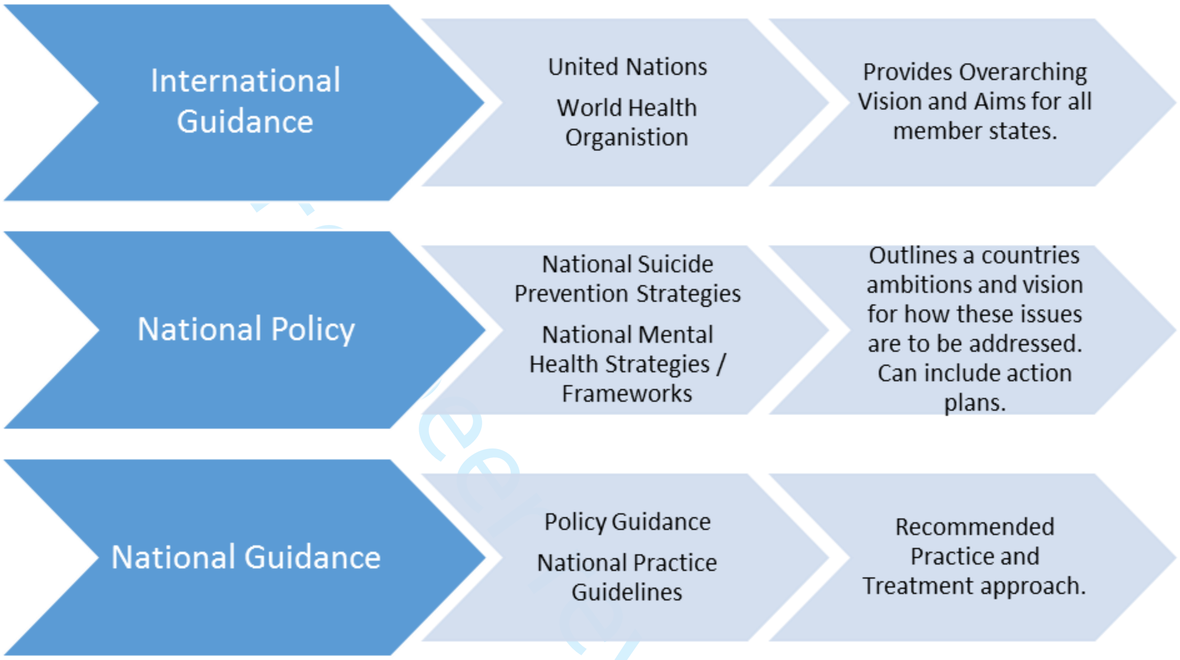

Supplementary table 1: Mesh Terms & database search results.						
Database	SEARCH TERMS	HITS after Duplicates removed	To be Read Full Text	Need to access abstract	Rejected = Wrong Topic / Sample / Published prior to 2000	Included studies after screening in FT & Accessing Abstracts
CINAHL via EBSCO	S3 S1 AND S2 Search modes - Boolean/Phrase<br>S2 (MH "Policy Studies+") OR (MH "Public Policy+") OR (MH "Health Policy Studies") OR (MH "Policy Making") OR (MH "Health Policy+") OR "Policy" OR (MH "Hospital Policies+") OR (MH "Guideline Adherence") Limiters - English Language; Language: English; Age Groups: Child: 6-12 years, Adolescent: 13-18 years Expanders - Apply equivalent subjects; Apply related words Search modes -	193	7	9	177	2

For peer review only

**Supplementary table 1: Mesh Terms & database search results.**						
Database	SEARCH TERMS	HITS after Duplicates removed	To be Read Full Text	Need to access abstract	Rejected = Wrong Topic / Sample / Published prior to 2000	Included studies after screening in FT & Accessing Abstracts
	☐ S1 (MH "Suicide+") OR (MH "Suicide, Attempted") OR (MH "Suicidal Ideation") Boolean/Phrase<br><br>Limiters - English Language; Language: English; Age Groups: Child: 6-12 years, Adolescent: 13-18 years<br><br>Expanders - Apply equivalent subjects; Apply related words<br><br>Search modes - Boolean/Phrase					

For peer review only

For peer review only - http://bmjopen.bmj.com/site/about/guidelines.xhtml

Supplementary table 1: Mesh Terms & database search results.						
Database	SEARCH TERMS	HITS after Duplicates removed	To be Read Full Text	Need to access abstract	Rejected = Wrong Topic / Sample / Published prior to 2000	Included studies after screening in FT & Accessing Abstracts
COCHRANE	1. #1<br>"suicide" or Suicidal:ti,ab,kw (Word variations have been searched)<br>2. #2<br>child* or teen* or adolesc* or young: ti,ab,kw (Word variations have been searched)<br><br>3. #3<br>Policy or Guidance or Strategy or Procedure: ti,ab,kw (Word variations have been searched)<br>4. #4<br>#1 AND #2 AND #3	49 Reviews	0	0	49	0
Medline	S4 = S1 AND S2 Limiters: English<br><br>S3 = S1 AND S2<br><br>S2 = DE "Government Policy Making" OR DE "Education Policy" OR DE "Foreign Policy	12	0	0	12	0

For peer review only - http://bmjopen.bmj.com/site/about/guidelines.xhtml

Supplementary table 1: Mesh Terms & database search results.						
Database	SEARCH TERMS	HITS after Duplicates removed	To be Read Full Text	Need to access abstract	Rejected = Wrong Topic / Sample / Published prior to 2000	Included studies after screening in FT & Accessing Abstracts
	Making" OR DE "Laws" OR DE "Legislative Processes" OR DE "Older Americans Act" OR DE "Welfare Reform" OR DE "Policy Making" OR DE "Environmental Policy" OR DE "Government Policy Making" OR DE "Health Care Policy" OR DE "Health Care Policy" OR DE "Health Care Reform<br><br>S1= DE "Suicide" OR DE "Assisted Suicide" OR DE "Suicidology					
PsychInfo	TOTAL SUM of S6 and S5 after duplicates removed<br><br>S6= S1 AND S2 Narrowed by: Age (6-12yrs); Age Adolescence (13-17); Language – English<br><br>S5 = S1 AND s2 Narrowed by Age Adolescence (13-17); Language – English.<br><br>S4 = S1 AND s2<br>S3=S1 AND S2<br><br>S2 = DE "Government Policy Making" OR DE "Education Policy" OR DE "Foreign Policy Making" OR DE "Laws" OR DE "Legislative Processes" OR DE "Older Americans Act" OR	43<br><br>(13)<br><br>(44)	2 But duplicates of studies found on CINAHL.	0	41	0

For peer review only - http://bmjopen.bmj.com/site/about/guidelines.xhtml

Supplementary table 1: Mesh Terms & database search results.						
Database	SEARCH TERMS	HITS after Duplicates removed	To be Read Full Text	Need to access abstract	Rejected = Wrong Topic / Sample / Published prior to 2000	Included studies after screening in FT & Accessing Abstracts
	DE "Welfare Reform" OR DE "Policy Making" OR DE "Environmental Policy" OR DE "Government Policy Making" OR DE "Health Care Policy" OR DE "Health Care Policy" OR DE "Health Care Reform"<br><br>**S1** = DE "Suicide" OR DE "Assisted Suicide" OR DE "Suicidology"					

For peer review only - http://bmjopen.bmj.com/site/about/guidelines.xhtml

Supplementary Table 2: Reasons for Exclusion			
Source	Total number excluded	Reason for Exclusions	Number
International Policy Documents	4	Not Transferable	2
		Not about suicidal CYP	1
		A review of reviews of interventions	1
UK Policy Documents	3	Not about suicidal CYP	2
		Newer version of report available	1
Journal Articles from Databases	6	Not a policy document or review of policy	5
		Not about suicidal CYP	1
From Experts	2	Not about suicidal CYP	2

For peer review only

Supplementary Table 3: Data Extraction Template.		
	Info to be extracted	Data
Document Classification	Title of Document	
	Authors	
	Year of Publication	
	Years policy covers	
	Country	
	Is this a new strategy / document or an update of a previous version?	
	Type of Policy Document. (Is it a Policy / Strategy / Guidelines / Review?	
Policy Drivers	Policy or document purpose and Aims. (Does it include any reference to CYP suicide?)	
	Who is subject to the policy, and is adherence compulsory? (Is it aimed at clinicians or services?)	
	Does the policy cover the life-span or is it targeted at a specific age-range?	
	Is this policy universal, selective, or indicated? (WHO, 2010) Or does it include all aspects of prevention activity.	
	Does the policy relate to or feed into any other policies or programs?	
OUTCOMES	Does the policy / strategy / guidance have evaluation measures built in?	
	Who is accountable for ensuring that the policy / strategy is implemented?	
Key concepts for Review Questions	How does the policy / document relate to suicidal CYP? e.g. It is about mental health and well-being for CYP OR It is about CAMHS services OR It is about Suicide	

For peer review only

	What does the policy / document say specifically about the treatment and care of CYP who are suicidal?	
Definitions	Are key-words clearly defined and what definitions are used for self-harm for example?	

*WHO, (2010), Towards Evidence Based Practice, World Health Organisation, Western Pacific Region.

For peer review only

For peer review only - http://bmjopen.bmj.com/site/about/guidelines.xhtml

Supplementary Table 4: Mapping the policy documents

Policy Classification	Country	Author	Document Name	Year	Summary of Document Purpose	How does the policy relate to CYP who are suicidal?	How does the policy address the treatment and care needs of CYP who are suicidal?
International Guidance.	Worldwide	United Nations	Transforming our world: The 2030 Agenda for sustainable development	2015	Sustainable development goals and action plan that all countries who are members of the united nations have agreed to implement. These goals and actions support working towards a world free from poverty, hunger and inequality, where everyone has equal human rights.	Goal 3.4: to reduce premature mortality from non-communicable diseases by one third.	It does not specifically mention children and young people who are suicidal.
	Worldwide	WHO	Mental Health Action Plan 2013-20	2013	An action plan to provide leadership and direction in the prevention, treatment and care of mental disorders, It aims to promote the human rights of	It recognises young people as being a priority group in relation to suicide prevention and intervention.	It states that countries should develop suicide prevention strategies, that give particular consideration to at risk groups – and specifies "youth".<br>It speaks to children with mental disorders having

For peer review only - http://bmjopen.bmj.com/site/about/guidelines.xhtml

					persons suffering mental ill health, and mental well-being generally.		access to psycho-social, non-medicalised treatments, but does not provide detail as to whether children who are suicidal are to be considered within this group.
	Worldwide	WHO	Preventing Suicide a Global Imperative	2014	Resource to encourage member states to develop suicide prevention strategies.	It identifies that suicide is major health issue for young people.	Provides a description of generic treatment options that may be considered helpful for all persons across the lifespan who have attempted or are thinking about suicide. (These are taken from the WHO's mhGAP Intervention Guide (2010)) It notes in relation to assessment of individuals who present as suicidal that this should be conducted in all person aged 10 years and above, but does not state why this age was selected. Otherwise there is no specific direction regarding the care and treatment of

For peer review only - http://bmjopen.bmj.com/site/about/guidelines.xhtml

							children and young people who are suicidal.
	Worldwide	WHO	mhGAP Intervention Guide for mental, neurological and substance use disorders in non-specialized health settings V2	2016	A model guide of effective and evidence based interventions to be adapted for local context.	It identifies children and adolescents as being a "special population". It does not differentiate between treatment and interventions for children and young people and adults who are suicidal. It offers assessment and intervention guidance which are the same for every-one over ten years of age. It does not state why age ten is used as a marker.	There is a module dedicated to Child & Adolescent Mental & Behavioural Disorders. Within this module there are two mentions of suicide:<br>1) Under the heading "2.5 Psychoeducation for emotional problems/disorders including depression in adolescents" (WHO, 2016, p 88) it states that if the YP notices thoughts of suicide then they should talk to someone they trust, and re-attend for mental health support.<br>2) Within a list a symptoms to assess for, when assessing for moderate to severe depression in adolescents.<br>There is a module detailing the assessment, management and follow up for people who might

For peer review only - http://bmjopen.bmj.com/site/about/guidelines.xhtml

							be at high risk of self-harming and or suicide.
National Suicide Prevention Strategies	Australia	Department of Health and Ageing – Australian Government.	Life: A Framework for prevention of Suicide in Australia	2007	National suicide prevention strategy covering the life-span.	This is an over-arching suicide prevention strategy and it does not mention CYP who are suicidal specifically. References to young people include providing support for children who are survivors of childhood abuse, and taking a whole school approach to children's mental health and wellbeing (prevention).	Not specific to children and young people who are suicidal.
	England	Department of Health	Preventing suicide in England: A cross-government outcomes strategy to save lives	2012	Sets out the government's objectives and action plan to reduce suicide rates.	Identifies CYP as a priority group. Within a section dedicated to CYP the policy gives demographic and background information about higher at-risk groups of CYP; those who have or are being bullied, children who	Mostly refers to universal and targeted prevention approaches; whole school approaches etc. and generic mental health provision. It details government approaches to tackling underlying issues such as bullying and child abuse. It does not directly address interventions and

For peer review only

						are looked after and accommodated etc.	treatments specifically for CYP who are actively suicidal other than stressing that early identification and assessment of at risk children vital.
	Ireland	National Office for Suicide Prevention	Connecting For Life: Ireland's National Strategy to Reduce Suicide 2015-2020	2015	National strategy to prevent suicide. Sets out national vision and action plan.	This policy outlines an action plan for delivering universal, targeted and integrated services for individuals who are suicidal and specifies youth services as being a priority area.	Specifically in relation to interventions for CYP who are suicidal the policy states that there should be "enhanced supports" and "enhanced availability". It recommends early intervention and psychological supports being provided and available to CYP who are at risk of suicide at both the primary care level, and secondary level including CAMHS.
	Northern Ireland	Department of Health	Protect Life 2	2016	This document is an update on the previous national suicide prevention strategy and covers the period 2016-2021. Its sets out five key aims: understanding suicidal behaviour	This strategy covers the lifespan, and includes CYP. It provides demographic information on CYP and suicide and self-harm in NI.	It notes that CYP are not a priority group for suicide prevention as the overall numbers of suicide amongst this population are low. It recognises however, that there has been an increase in the numbers of CYP who die by

For peer review only - http://bmjopen.bmj.com/site/about/guidelines.xhtml

					in NI better; improve responses to people who are suicidal; prevent suicide; support recovery; support those bereaved by suicide.		suicide and the high numbers of CYP who self-harm. In relation to treatments and care of CYP who are suicidal the strategy states that if those working with CYP are able to better identify risk factors and there is easier access to CAMHS services this would support prevention. It also outlines the role of CHILDLINE in providing support and counselling to CYP, and Crisis Intervention and Support Teams to assess children who present at hospital. It does not provide clear direction in the treatment and care of CYP who are suicidal.
	New Zealand	Associate Minister of Health	New Zealand Suicide Prevention Strategy	2006	National Strategy for suicide prevention.	This document replaces the New Zealand Youth Suicide Prevention Strategy, and moves to a generic life-span approach, although acknowledges that there may be priority	This document does not specifically address the care and treatment needs of children and young people who are suicidal. It provides a review of the literature, the context for suicide prevention, and sets

For peer review only

						groups. In a section headed "Issues relating to Age" p19, it states that young people remain a focus as they still have high rates of suicide amongst this population.	seven goals which are applicable across the life span. These goals include improved care for people who are suicidal.<br><br>Within a section headed "context" it states that although moving to a universal approach, targeted interventions for youth will still be needed.
	New Zealand	Associate Minister of Health	A Strategy to Prevent Suicide in New Zealand: A draft public consultation document.	2017	The draft framework for a new National Suicide Prevention Strategy that has three main elements. It outlines a vision (everyone will have a life worth living); a purpose (to reduce the suicide rate); to provide pathways (to increase protective factors and reduce risk).	Young People aged 15-24 are recognised as a target group.	One of the key objectives of the strategy is to recognize and support people in distress and to support people after a suicide attempt or self-harm. Of the prevention activities that are described that may be helpful that pertain specifically to CYP the focus is on improving support in the school setting: supporting teachers to respond to students who are distressed or following a suicide attempt; training

For peer review only - http://bmjopen.bmj.com/site/about/guidelines.xhtml

							teachers to talk to students who are distressed or been affected by suicidal behaviour; partnering psychologists and counsellors with schools to improve access to psychological support for CYP.
	USA	U.S. Surgeon General and the National Action Alliance for Suicide Prevention	2012 National Strategy for Suicide Prevention: GOALS AND OBJECTIVES FOR ACTION	2012	National Suicide Prevention Strategy which aims to guide suicide prevention activity.	The policy document includes suicide prevention guidance for youth, but is generic in its approach.	The policy outlines demographic information that highlights that suicidal behaviours are more common amongst young people aged 15-24yrs than adult populations.<br>Goal 1 – includes references to school based prevention programs, but not interventions.<br>Goal 5 - refers to the need to develop interventions for all groups of people at risk, and that these should be sensitive to and designed around the different needs of these groups. This is not

For peer review only - http://bmjopen.bmj.com/site/about/guidelines.xhtml

							specific to children and young people.
	Scotland	Scottish Government	Suicide Prevention Strategy	2013-2016	An update on previous national suicide prevention strategy (The first of which was 2003). A list of commitments and recommendations.	This document sets out the government is committed to reducing suicide and sets out a target of improving responses to people in distress.	It doesn't mention children and young people specifically.
	Sweden	Public Health Agency of Sweden	NATIONAL ACTION PROGRAMME FOR Suicide prevention	2008	This is taken form a brochure of the National Strategy, as the actual strategy is not available in English.	The national strategy has nine overall objectives, and states that their overall aim is that no-one should feel suicide is the only option they have. It recognizes young people as being a high risk group".	The brochure states that the suicide rate amongst 15-24year olds has remain unchanged, despite a reduction in the overall suicide rate over the last 15years. It does not differentiate between the treatment and care that should be given to CYP who are suicidal, although does mention under the heading "Improve medical, psychological and psychosocial initiatives" amongst a list of example initiatives some with a particular youth / student focus.
	Wales	Welsh Government	Talk to Me 2: Suicide and Self	2015	National Strategy to prevent suicide.	The strategy covers the life span, but	Demographic background information

For peer review only - http://bmjopen.bmj.com/site/about/guidelines.xhtml

			Harm Prevention Strategy for Wales 2015-2020		An update on a previous policy.	identifies that certain groups of CYP are priority: Looked after children, Care leavers, Children and young people in the Youth Justice System Bullied or victimised children and young people, Survivors of abuse or violence including sexual abuse and domestic violence and children and young people with a background in vulnerability.	is provided. Strategy focuses upon universal school based prevention programmes and targeted prevention activities such as school based counselling services. In relation to CYP who are suicidal it states that services should be accessible for those in crisis.
National Mental Health Strategies.	CANADA	Mental Health Commission	Changing Directions Changing Lives: The Mental Health Strategy for Canada.	2012	National Mental Health Strategy covering the lifespan.	This policy recognises suicide as being an important issue and addresses it directly. It also recommends CYP are a priority group.	Sets out objectives which include improved screening and assessment for suicide, and improved access to services. These are generic recommendations for all age groups. In relation to children and young people specifically it states that governments should address inequality in accessing to services psychotherapies

For peer review only - http://bmjopen.bmj.com/site/about/guidelines.xhtml

							and counselling. The situation is described as urgent, with families who do not have access to financial resources having to wait up to a year for treatment. This is not specific to suicidal children and young people.
	CANADA	Mental Health Commission	The Mental Health Strategy for Canada: A Youth Perspective	2015	National Mental Health Strategy written from the perspective of young people. Translates the original policy document into lay language.	Generic document – addresses mental health generally, but preventing suicide is identified as a priority. Talks about access to services and specialist help being available to people when they need it.	Not specific to the treatment and care of suicidal children and young people but states that mental health services should be more accessible to people of all ages.<br>It highlights examples of good practice in service provision and suicide prevention for children and young people. E.g. The Thunder Bay Youth Suicide Prevention Task Force (30 organisations working collaboratively to prevent suicide and provide an immediate response) & Youth advocates (Young people with lived experience).

For peer review only - http://bmjopen.bmj.com/site/about/guidelines.xhtml

							It addresses race and cultural issues in relation to suicide risk for YP: increased risk for Inuit and Metis young people. It refers to organic culturally sensitive initiatives such as The Inuit Tapirit Kanantami National Youth Council, and community health resources that offer programs of support to children and young people at risk of suicide.
	England	HMG/DH	No health without mental health A cross-government mental health outcomes strategy for people of all ages	2011	National Mental Health Strategy across the life span. Aims to address the disparity between how mental health and physical health are treated. Has six main objectives. Aims to mainstream mental health.	Adopts a lifespan approach but identifies CYP as a group with particular needs, and recommends age appropriate services and interventions.	Gives background demographic information on mental health issues including the numbers of children and young people who have self – harmed. States that all people who come into contact with CYP should be aware of the issues in relation to self-harm, as they do not always attend hospital. States that reducing suicide remains priority government target.

							Reduced numbers of people of all ages harming themselves is set as a measure of success of the strategy.<br><br>No specific references to CYP who are suicidal.
	Ireland	Department of Health	A vision for change	2006	Provides a vision and model for mental health service provision.	This document addresses a wide range of mental health issues and service provision. Within this there is a chapter dedicated to the provision of mental health services for CYP (Ch. 10) and a section dedicated to Suicide Prevention within Ch. 15 on Special Categories. It does not identify CYP as being a priority group.	Within Ch.10 it states that all children who present with deliberate self-harm should be assessed by the child and adolescent mental health team, and if appropriate receive treatment. Within this section it also recognises adolescence as being a period of increased risk of suicide.
	Scotland	Scottish Government	Mental Health Strategy	2017 - 2020	National Mental Health Strategy covering the life span.	The documents provides a focus on prevention and early intervention for children and young people. It includes actions relating to	It does not specifically address the treatment and care needs of CYP who are suicidal. It states that Suicide and Self-Harm continue to be

						support being available in schools, delivery of CAMHS services, and access to support and services when they are needed.	a priority area that will be addressed in a new suicide prevention strategy in 2018.
	Wales	Welsh Government	Together for Mental Health	2016	Ten year National strategy for Mental Health. Aims to improve the mental health and well-being of people of all ages in Wales adopting a life span approach to policy.	The policy addresses generic mental health needs and provision of mental health support services for CYP. It highlights the need for targeted interventions for those most at risk, and early identification and prevention.	It does not specifically address the treatment and care needs of CYP who are suicidal.
National Frameworks for Children & Adolescent Mental Health	CANADA	Mental Health Commission Canada	Evergreen: A Child and Youth Mental Health Framework for Canada	2010	Framework of values and strategic direction for the provision of mental health services for children and young people.	Policy concerned with promoting mental health and well-being amongst young people, and provision of mental health support services for CYP and families across Canada, but does not specify suicidality.	Does not mention the treatment and care of CYP who are suicidal. Lists recommendations about making services accessible (available in the evenings and weekends, accessible to YP directly, and child / youth centred), including creating "one-stop shops", however

For peer review only - http://bmjopen.bmj.com/site/about/guidelines.xhtml

							does not specify children and young people who are suicidal. The only reference to suicide is in a direct quote by a young person talking about how there is a lack of education about mental health issues and that many Aboriginal young people are largely unaware of mental health issues but have high rates of suicide.
	England	Department of Health	National Service Framework for Children, Young People and Maternity Services: The Mental Health and Psychological Wellbeing of Children and Young People, Department of Health and Department for Education and Skills (2004) –	2004	This is part of the national framework for standards for child health services. It is the standard for children who need to access mental health and psychological services,	This standard sets out a vision for all CYP to have access to specialist mental health support services when they need it. It only directly refers to CYP who are suicidal once in relation to out of hours support.	The standard is not specific to CYP who are suicidal, however it does state that CYP should have access to support services when they need them, that they should have time to build a therapeutic relationship, and that non-attendance of clinical appointments should not be a trigger for closing cases. The standard states that services need to be creative and flexible in how they engage with CYP.

For peer review only - http://bmjopen.bmj.com/site/about/guidelines.xhtml

	England	Department of Health & Department of Education	Transforming Children and Young People's Mental Health Provision: A Green Paper	2017	Green paper proposing reform to child and adolescent mental health service provision: the appointment of mental health leads in each school; creation of support teams to asses and act as a bridge between school and CAMHS services; trial a four-week waiting time for CAMHS specialist services.	Policy update to address CYP experiencing distress and or in "crisis" being able to access the right support when they need it but does not directly refer to suicide.	Recommendations about the treatment and care of CYP experiencing distress or being in crisis could be applied to CYP who attempted or are actively thinking about suicide, but it does not specifically address suicidal CYP. Under a section entitled "Improving Crisis Care" there is reference to pilot models of crisis support underway, a new government funded scheme called "Beyond Places of Safety" for people of all ages who are in crisis, and the commissioning of the development of generic and crisis mental health pathways for CYP.
	Scotland	Scottish Government	The Mental Health of Children and Young People: A Framework for Promotion, Prevention and Care	2005 (Due to be updated)	National framework to be used as a guide for local authorities and commissioners of children's services to guide service design and delivery.	Takes a holistic view of children's mental and health and well-being and recognises the role that everyone in contact with CYP have in supporting their mental health.	No specific references to CYP who are suicidal.

For peer review only - http://bmjopen.bmj.com/site/about/guidelines.xhtml

	Wales	Welsh Government	Together for Children and Young People: Framework for Action.	2015	National Framework for child and adolescent mental health support services.	The framework provides multi agency guidance for the provision of children and young people's mental health and well-being.	It does not specifically address the treatment and care of children and young people who are suicidal. It does state that there should be a specialist CAMHS pathway, and this includes out of hours support for CYP in crisis.
Guidance Documents	Ireland	Health Service Executive & Child and Adolescent Mental Health Service.	Child and Adolescent Mental Health Services Standard Operating Procedure	2015	Provide clear direction for the delivery of CAMHS services across Ireland, to ensure consistent and transparent approach.	This document outlines the standard operating procedures for CAMHS services in Ireland, and the treatment and services that CYP and their families should expect. This includes CYP who are suicidal.	Generic statements about CAMHS services being accessible, and response times especially for situations where there may be a risk to the young person and an immediate response is required could be applicable to CYP where there is a risk of suicide but this is not clearly stated. The treatment of CYP who are suicidal is only specifically addressed in reference to the fact that CAMHS will accept referrals for CYP with "suicidal behaviours and ideation where intent is present" p15, and in terms of risk

For peer review only - http://bmjopen.bmj.com/site/about/guidelines.xhtml

							management listing risk of suicide amongst potential risk behaviours within an in-patient unit.
	New Zealand	Ministry of Health & New Zealand Guidelines Group	The Assessment and Management of People at Risk of Suicide	2003	These National Guidelines are a resource and guide for clinical staff in emergency departments, and psychiatric services working with people in mental distress who are at high risk of suicide or have attempted suicide.	This document specifically states how all persons who are suicidal should be treated and has a small section referencing the treatment of children and adolescents within it.	These guidelines provide specific recommendations on how to respond to and treat anyone who is suicidal, including children and young people. It states that anyone talking about suicide should be taken seriously.<br>In particular to CYP it states:<br>“Self-harm among children is rare and should be treated very seriously…The assessment of suicidal young people should be carried out by a clinician who is skilled in interviewing and working with children and adolescents whenever possible….Risk assessments should draw on information from multiple sources, including the young

For peer review only - http://bmjopen.bmj.com/site/about/guidelines.xhtml

							person, their teachers/guidance counsellors, parents etc."<br>In the section on background information following the guidance it notes that New Zealand has one of the highest rates of suicide amongst young people.
	UK	NICE	Self-harm in over 8s: short-term management and prevention of recurrence	2004	Clinical guidelines for the management of self-harm in people over 8yrs in the first 48hrs following the incident of self-harm. These guidelines apply to everyone regardless of whether there is an underlying mental health illness.	The definition of Self-Harm used includes all acts of self-harm regardless of suicidal intent. These guidelines therefore apply to CYP (8-16yrs) who have attempted suicide, although this is not specifically stated.	It recommends that CYP who have self-harmed are admitted overnight to a paediatric ward (potentially adolescent ward if preferred by those aged 14yrs+), and fully assessed before discharge. The guidelines state that they should be assessed by someone trained and with experience in assessing CYP who have self-harmed. It states that assessment should be the same as for adults but also include holistic assessment of family situation etc. The only direct reference to suicide is contained

For peer review only - http://bmjopen.bmj.com/site/about/guidelines.xhtml

							within the generic description of "Assessment"; it is included in a list of factors to be assessed. It goes on to specify that child and adolescent mental health practitioners assessing CYP who have self-harmed should be trained, skilled in risk assessment, supervised regularly, and have access to senior support. Family are to be recommended to remove access to means of self-harm in the immediate aftermath of the incident.
	UK	NICE	Self-harm in over 8s: long term management and prevention of recurrence	2004	Guidelines for the long-term psychological treatment and management of self-harm. It aims to improve the quality of care that they receive, and reduce repetition of self-harm.	The definition of Self-Harm used includes all acts of self-harm regardless of suicidal intent. These guidelines therefore apply to CYP (8-16yrs) who have attempted suicide, although this is not specifically stated.	Under the heading "Risk Assessment" there are two references to suicide, which it states should be assessed in relation to risk (risk of self-harm & past and present suicidal intent). However it warns against using risk assessment tools to assess this risk.

For peer review only - http://bmjopen.bmj.com/site/about/guidelines.xhtml

							Under the heading "Access to Services" it states that children and young people who self-harm should be able to access psychological therapies and treatments from child and adolescent mental health services."<br><br>CAMHS workers who are working with CYP who self-harm should consider safeguarding procedures, and where children are referred to CAMHS under safeguarding procedures then there should be a multi-agency approach and consideration of common sense framework.<br><br>Under the heading "Primary Care" it recommends that consideration be given to referring CYP who present with self-harm to CAMHS for specialist help. Factors to consider

For peer review only - http://bmjopen.bmj.com/site/about/guidelines.xhtml

							are the parent or carers' distress / anxiety.
	USA	Chun et al, American Academy of Paediatrics	Evaluation and Management of Children and Adolescents With Acute Mental Health or Behavioral Problems. Part I: Common Clinical Challenges of Patients With Mental Health and/or Behavioral Emergencies	2016	To provide clinical guidance to staff in the USA primarily working in A & E departments, but for all clinicians who work with children with emotional and behavioural problems on dealing with paediatric mental health emergencies. It is not intended to be used as a standard of care and is a recommended practice as opposed to enforced.	This document has a section dedicated to dealing with children who present to the Emergency Department in hospital following a suicide attempt.	This document provides very specific recommendations on the assessment and care of children where there has been a suicide attempt. This includes medical assessment as well as suggestions for psychological assessment. It states that although there is no recognised effective risk assessment tool for suicide, it lists criteria such as not engaging in safety planning and remaining intent upon dying that if present suggest the child should be admitted to hospital for their own safety. It suggests that otherwise the child might be referred for outpatient treatment and that if this is unavailable or waiting times are long mental home providers (Patient centred care co-ordinated by their own

For peer review only - http://bmjopen.bmj.com/site/about/guidelines.xhtml

							doctor) are encouraged to provide support in the interim.
	USA	Michael L. LeFevre, MD, MSPH, on behalf of the U.S. Preventive Services Task Force*	Screening for Suicide Risk in Adolescents, Adults, and Older Adults in Primary Care: U.S. Preventive Services Task Force Recommendation Statement.	2014	Recommended clinical guidance for primary care settings in relation to the assessment of suicide risk.	Specifically addressed assessing the suicide risk in adolescents.	There is limited evidence for any treatments for adolescents at risk of suicide, and not enough evidence to support the potential benefit or harm of either screening for risk of suicide, or not.
Miscellaneous reports about policy.	Northern Ireland	Dr Jennifer Betts and Dr Janice Thompson	Mental Health in Northern Ireland: Overview, Strategies, Policies, Care Pathways, CAMHS and Barriers to Accessing Services	2017	This paper provides background information on mental health and illness in Northern Ireland (NI) including demographics on Suicide and self-harm. It details the relevant strategies and policies. It describes the care pathway for treating mental health problems, with specific reference to child and adolescent mental health	Reports on recent changes in the delivery of CAMHS services and attempts to make them accessible. Early intervention is highlighted as a priority. Provides an overview of key mental health policies and refers to objectives to reduce the number of people presenting with self-harm, and suicide prevention activity.	Gives demographic information highlighting that suicide and self-harm are issues for young people in Northern Ireland. Not specific to suicide, but it states that a referral care pathway for CAMHS is currently being developed.

For peer review only - http://bmjopen.bmj.com/site/about/guidelines.xhtml

					services (CAMHS); discussing barriers to accessing services.		
	Scotland	Scottish Government	Responding to self-harm in Scotland: Mapping out the next steps	2011	This document is a report from the National Self Harm working group who were tasked with mapping out the next stages following publication of the Truth Hurts in 2006.	This document is a recommendation for policy. It calls for improved responses to people who self-harm across the life span, and recognizes that for most people who self-harm they do not intend to die.	Provides background information and demographic information on self-harm rates in Scotland. It states that young people are more likely to self-harm. Within the proposed objectives for future actions there are recommendations for clear referral pathways for all people who self-harm but is not specific to CYP. The only direct references to the treatment and care of CYP are recommendations on guidance on confidentiality and information sharing where a child is at risk.
	New Zealand	Prime Minister's Chief Science Advisor – Ch. 16: Keren Skegg.	Improving the Transition Reducing Social and Psychological Morbidity During Adolescence	2011	This is a report commissioned by the government to review the evidence on how to improve the	Ch. 16 is devoted to Youth Suicide. It aims to give an overview of the issue and prevention strategies.	Ch.16 of the report considers how the New Zealand Suicide prevention strategy goals apply to young people. It takes each

For peer review only - http://bmjopen.bmj.com/site/about/guidelines.xhtml

					outcomes for adolescents transitioning to adulthood.		goal in turn and cogitates how it focuses on youth. In conclusion it argues that the strategy involves actions that can be applied to the issues of youth suicide. It also argues that any programmes aimed at young people must be evidence based.

For peer review only - http://bmjopen.bmj.com/site/about/guidelines.xhtml

Preferred Reporting Items for Systematic reviews and Meta-Analyses extension for Scoping Reviews (PRISMA-ScR) Checklist

SECTION	ITEM	PRISMA-ScR CHECKLIST ITEM	REPORTED ON PAGE #
**TITLE**			
Title	1	Identify the report as a scoping review.	
**ABSTRACT**			
Structured summary	2	Provide a structured summary that includes (as applicable): background, objectives, eligibility criteria, sources of evidence, charting methods, results, and conclusions that relate to the review questions and objectives.	
**INTRODUCTION**			
Rationale	3	Describe the rationale for the review in the context of what is already known. Explain why the review questions/objectives lend themselves to a scoping review approach.	
Objectives	4	Provide an explicit statement of the questions and objectives being addressed with reference to their key elements (e.g., population or participants, concepts, and context) or other relevant key elements used to conceptualize the review questions and/or objectives.	
**METHODS**			
Protocol and registration	5	Indicate whether a review protocol exists; state if and where it can be accessed (e.g., a Web address); and if available, provide registration information, including the registration number.	
Eligibility criteria	6	Specify characteristics of the sources of evidence used as eligibility criteria (e.g., years considered, language, and publication status), and provide a rationale.	
Information sources*	7	Describe all information sources in the search (e.g., databases with dates of coverage and contact with authors to identify additional sources), as well as the date the most recent search was executed.	
Search	8	Present the full electronic search strategy for at least 1 database, including any limits used, such that it could be repeated.	
Selection of sources of evidence†	9	State the process for selecting sources of evidence (i.e., screening and eligibility) included in the scoping review.	
Data charting process‡	10	Describe the methods of charting data from the included sources of evidence (e.g., calibrated forms or forms that have been tested by the team before their use, and whether data charting was done independently or in duplicate) and any processes for obtaining and confirming data from investigators.	
Data items	11	List and define all variables for which data were sought and any assumptions and simplifications made.	
Critical appraisal of individual sources of evidence§	12	If done, provide a rationale for conducting a critical appraisal of included sources of evidence; describe the methods used and how this information was used in any data synthesis (if appropriate).	
Synthesis of results	13	Describe the methods of handling and summarizing the data that were charted.	

St. Michael's
Inspired Care. Inspiring Science.

For peer review only - http://bmjopen.bmj.com/site/about/guidelines.xhtml

SECTION	ITEM	PRISMA-ScR CHECKLIST ITEM	REPORTED ON PAGE #
**RESULTS**			
Selection of sources of evidence	14	Give numbers of sources of evidence screened, assessed for eligibility, and included in the review, with reasons for exclusions at each stage, ideally using a flow diagram.	
Characteristics of sources of evidence	15	For each source of evidence, present characteristics for which data were charted and provide the citations.	
Critical appraisal within sources of evidence	16	If done, present data on critical appraisal of included sources of evidence (see item 12).	
Results of individual sources of evidence	17	For each included source of evidence, present the relevant data that were charted that relate to the review questions and objectives.	
Synthesis of results	18	Summarize and/or present the charting results as they relate to the review questions and objectives.	
**DISCUSSION**			
Summary of evidence	19	Summarize the main results (including an overview of concepts, themes, and types of evidence available), link to the review questions and objectives, and consider the relevance to key groups.	
Limitations	20	Discuss the limitations of the scoping review process.	
Conclusions	21	Provide a general interpretation of the results with respect to the review questions and objectives, as well as potential implications and/or next steps.	
**FUNDING**			
Funding	22	Describe sources of funding for the included sources of evidence, as well as sources of funding for the scoping review. Describe the role of the funders of the scoping review.	

JBI = Joanna Briggs Institute; PRISMA-ScR = Preferred Reporting Items for Systematic reviews and Meta-Analyses extension for Scoping Reviews.
* Where *sources of evidence* (see second footnote) are compiled from, such as bibliographic databases, social media platforms, and Web sites.
† A more inclusive/heterogeneous term used to account for the different types of evidence or data sources (e.g., quantitative and/or qualitative research, expert opinion, and policy documents) that may be eligible in a scoping review as opposed to only studies. This is not to be confused with *information sources* (see first footnote).
‡ The frameworks by Arksey and O'Malley (6) and Levac and colleagues (7) and the JBI guidance (4, 5) refer to the process of data extraction in a scoping review as data charting.
§ The process of systematically examining research evidence to assess its validity, results, and relevance before using it to inform a decision. This term is used for items 12 and 19 instead of "risk of bias" (which is more applicable to systematic reviews of interventions) to include and acknowledge the various sources of evidence that may be used in a scoping review (e.g., quantitative and/or qualitative research, expert opinion, and policy document).

From: Tricco AC, Lillie E, Zarin W, O'Brien KK, Colquhoun H, Levac D, et al. PRISMA Extension for Scoping Reviews (PRISMA-ScR): Checklist and Explanation. Ann Intern Med. ;169:467–473. doi: 10.7326/M18-0850
